# A model for phase boundary thermodynamics

**DOI:** 10.1039/d6ra07593b

**Published:** 2026-11-06

**Authors:** Jegatheesan Murugan, Theophilus Wallis, Reza Darvishi Kamachali

**Affiliations:** a Federal Institute for Materials Research and Testing (BAM) Unter Den Eichen 87 Berlin 12205 Germany reza.kamachali@bam.de; b Institute of Materials Physics, University of Münster Münster 48149 Germany

## Abstract

Phase boundaries separate thermodynamically distinct phases and can govern microstructural stability, transformation kinetics, and property evolution. Here, we extend the density-based thermodynamic and phase-field (DPF) framework developed for grain boundaries to phase boundaries (PBs). We consider a planar PB in a single-component system and characterize it through the minimum atomic density across the boundary region and phase-specific relative density fields referenced to the homogeneous bulk state of each adjoining phase. The PB Gibbs free energy functional is derived across the boundary width, defined as the region over which the atomic density varies. We present a numerical scheme for determining the equilibrium density profile across the PB and systematically identifying the model parameters. The effects of local stiffness, gradient-energy penalties, bulk-density mismatch, PB plane density, and reduced-traction contrasts on the PB localization lengths and the excess free energy are discussed. The connection between the continuum model and atomistics is demonstrated through Gaussian coarse-graining of an atomistic BCC–FCC Fe PB, from which the bulk densities, PB plane density, and geometric half-widths are extracted. A base-case calibration is used to identify the key coefficients of the PB free energy functional by fitting the model to the coarse-grained atomistic reference profile. The present framework provides an efficient route for studying segregation and interfacial phase behavior in multiphase systems. It enables the construction of phase boundary phase diagrams grounded in bulk reference states and parameterized from atomistic calculations.

## Abstract 2

A density-based, CALPHAD-compatible model for phase boundary thermodynamics.

## Graphical abstract

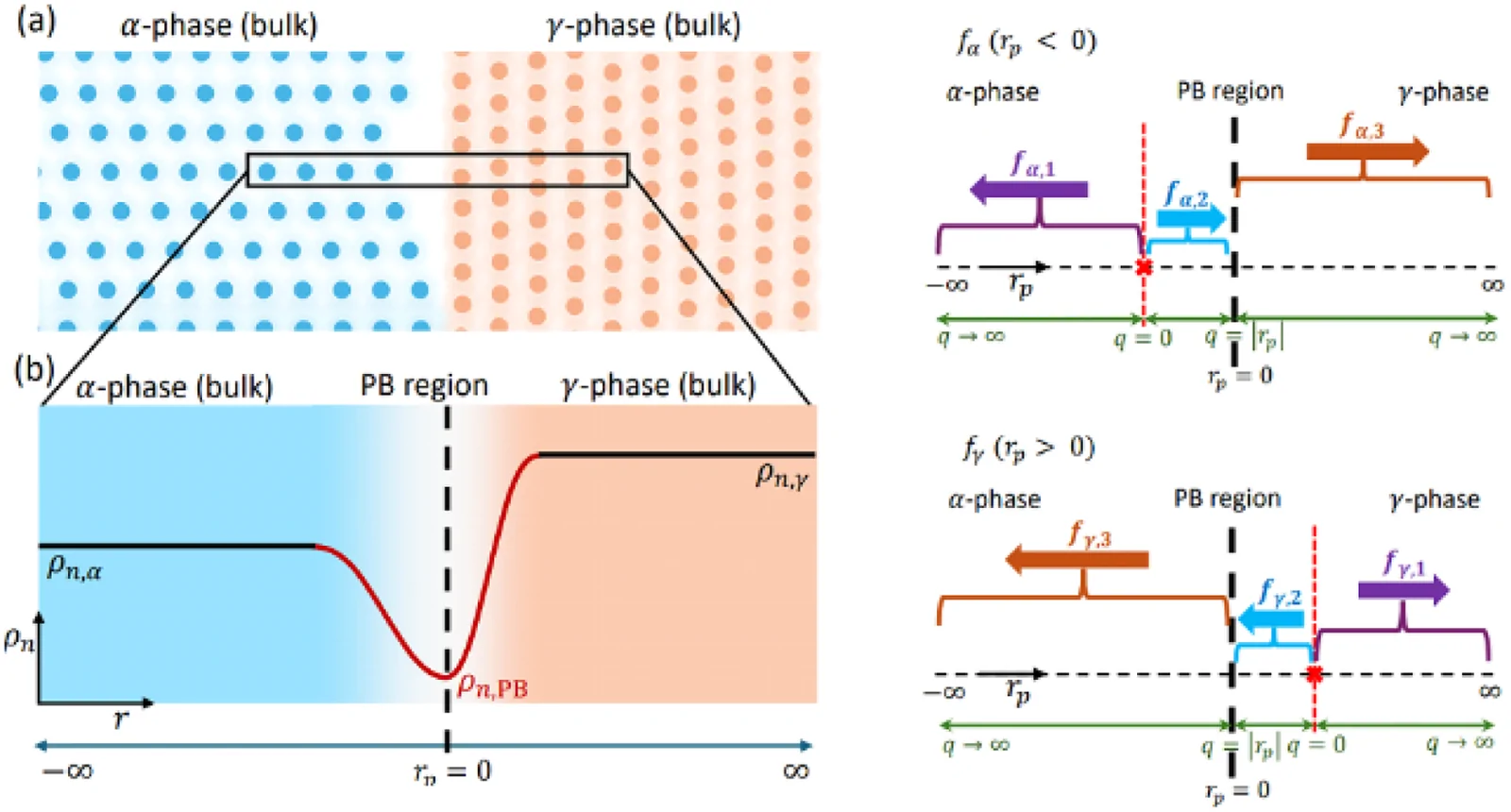

## 1. Introduction

Phase boundaries (PBs), also referred to as interphase boundaries, are ubiquitous internal interfaces in multi-phase alloys and functional materials. Unlike grain boundaries (GBs),^1^ PBs separate thermodynamically distinct phases (often with different equilibrium compositions, crystal structures, and properties) and therefore influence not only interfacial mechanics but also phase transformation pathways and microstructure stability.^2^ Consequently, the character of PBs strongly affects structure–sensitive properties, including strength,^2–4^ creep resistance,^5^ toughness,^6^ and functional performance.^7^ Despite their critical role, general and quantitative thermodynamic descriptions of PBs remain far less mature than bulk thermodynamics, limiting the systematic use of PB effects in alloy and microstructure design. This is especially consequential because PBs can possess structural and chemical states distinct from those of either adjoining bulk phase^8,9^ and can evolve through interactions with dislocations, precipitates, and neighboring interfaces.^10,11^

Recent work on interface complexions and atomically resolved PB structures further shows that PBs can accommodate segregation,^12^ undergo segregation-driven phase transformations,^13^ and promote interfacial precipitation.^14^ These processes can qualitatively alter the microstructure and, consequently, the resulting material properties. Such findings reinforce the need for rigorous, interface-aware thermodynamic models that treat PBs as thermodynamic entities rather than fixed geometric defects.

PB thermodynamics has been included in broader studies of interfaces and interfacial segregation,^15,17–21^ but has rarely been their main focus. Cahn and Hilliard,^22^ Cahn,^23,24^ and van der Waals^25^ developed interfacial free energy descriptions that can apply to PBs. A thermodynamic model was also proposed to predict amorphous-layer thickness at interfaces in binary systems.^26^

Hillert^27^ proposed a thermodynamic model to calculate the diffuse structure of interfaces and associated energy. Meijering^28^ calculated concentration profiles across coherent and incoherent interfaces in binary alloys using the nearest-neighbor model. Guttman^29–31^ developed thermodynamic models to calculate interfacial segregation patterns in multi-component systems. Johnson^32^ solved two coupled segregation equations with respect to the adjoining bulk phases of a PB to calculate the equilibrium segregation at the interfaces. Fecht and Gleiter^33^ proposed a “lock-in model” to correlate the interfacial energy with interface characteristics. Dregia and Wynblatt^34^ developed a gradient thermodynamic framework explicitly for PBs to calculate the PB energies and equilibrium PB compositions. The gradient energy approach was extended^35^ by accounting the atomic bonds inside and outside the interface to include solute drag effects on PB migration. A finite-width thermodynamic model^36^ was developed to compute the equilibrium segregation patterns at coherent PBs.

Most mesoscale phase-field models of interfacial segregation likewise focus on GBs.^37–42^ However, a few phase-field models for PBs have also been developed. Recently, based on Kim *et al.*,^43^ Kadambi *et al.* developed a phase-field model to compute equilibrium segregation at PBs in binary^44^ and ternary^45^ alloy systems.

Although the fundamental aspects of internal interfaces have been studied for decades, translating this knowledge into systematic thermodynamic and kinetic descriptions for materials design remains far less straightforward than for bulk phases. Bulk thermodynamic databases are mature, whereas comparable interface databases remain scarce. This is because interface behavior depends not only on chemistry and temperature, but also on crystallographic character and atomic structure. Since interfaces contain the same constituents as the adjoining phases, they can be treated as localized departures from bulk reference states rather than through independent interfacial free energy functions.

In a recent step toward anchoring interface thermodynamics to bulk reference states, Kamachali^1^ developed such a thermodynamically consistent density-based phase-field (DPF) model for GBs. In this formulation, the GB is represented by a continuous atomic-density field referenced to the homogeneous adjoining bulk phases, and the corresponding Gibbs free energy functional is constructed from bulk thermodynamic information. DPF has been successfully applied to study the GB segregation behavior in binary^1,46^ and multi-component^47–50^ alloy systems. Further, the model is used to study the segregation-assisted phase separation^46^ and the structural transition and associated phase decomposition at GBs^51^ in Fe–Mn alloy system. The model is also used to calculate the GB phase diagrams and segregation maps for ternary,^48,50,52^ Fe–Co–Mn–Cr^49^ and Fe–Mn–Ni–Co–Cr^47^ alloy systems. DPF was also used to study the segregation driven liquid metal embrittlement in Fe–Zn alloys.^53–55^

Extending the same bulk referenced approach to PBs is a natural next step, but it also introduces additional layers of complexity since a PB links two distinct bulk phases, typically with different equilibrium compositions, crystal structures, and reference densities, so the interfacial state must be formulated relative to two bulk reference states and remain consistent with the equilibrium and continuity constraints across the boundary. Developing a CALPHAD-integrated, bulk referenced thermodynamic description for PBs therefore hinges on a formulation that can represent the interfacial region as a localized perturbation of both adjoining phases while remaining consistent with their separate bulk thermodynamic descriptions. In this work, we introduce a continuous atomic density field, along with its spatial variations to characterize the PB region separating two homogeneous bulk phases (that differ in crystallographic character and atomic structure), relative to the adjacent bulk phases, in a pure metal. Based on this idea, we (i) compute the PB's Gibbs free energy, (ii) develop a method to link the model to the atomistic simulation, and (iii) systematically evaluate the model's performance.

## 2. Density-based model for a PB

PBs introduce several complexities beyond those encountered in GB systems. First, the density discontinuity across the interface is typically larger than in GBs, requiring careful treatment of the transition region. Second, the different phases may exhibit distinct crystallographic orientations, lattice parameters, or even crystal structures, leading to significant structural mismatch. Third, the thermodynamic driving forces for phase transformation introduce additional energy contributions that must be incorporated into the density field description.

However, these challenges also present opportunities for a more general and physically meaningful model. The continuous density field approach naturally accommodates the smooth transition between phases while preserving the essential physics of the interface. By treating the PB as a region of continuously varying density, we can capture both the local atomic arrangements and the thermodynamic equilibrium conditions.

In this work, we extend the GB-DPF framework by incorporating the fundamental asymmetry of PBs. We maintain the continuous density field description but account for the different equilibrium densities on either side of the interface. The model captures both the atomistically informed variation of the coarse-grained density in the interfacial core and the smooth recovery to the distinct bulk reference densities on either side. This combination provides a direct bridge between atomistic descriptions of PB structure and a continuum, bulk referenced thermodynamic functional for equilibrium PBs.

### 2.1. A density representation of a PB

We describe a planar PB between two phases *α* and *γ* of a pure substance A, as illustrated in Fig. 1(a), at constant temperature *T* and pressure *p*. The total Gibbs free energy is written as a sum of contributions from the *α* and *γ* regions,2.1
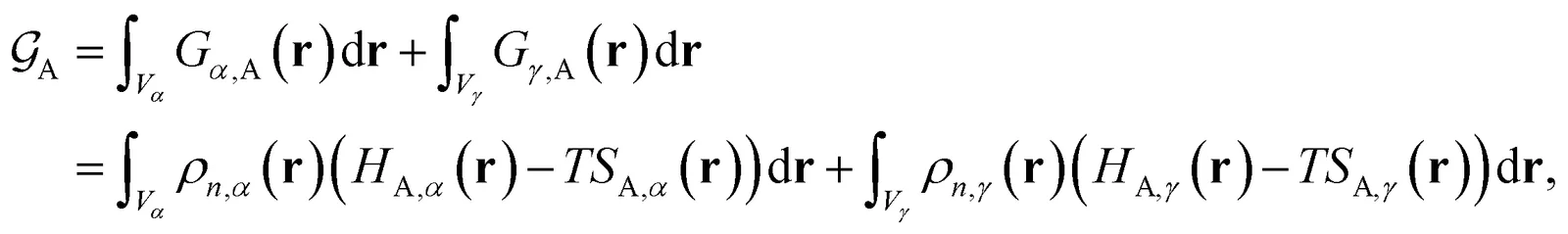
where *H*_A,*α*_ and *S*_A,*α*_ are the molar enthalpy and molar entropy of the *α* phase, with *H*_A,*α*_ = *E*_A,*α*_ + *K*_A,*α*_ + *pV*_A,*α*_ and *E*_A,*α*_, *K*_A,*α*_, *pV*_A,*α*_ is the potential energy, kinetic energy and mechanical energy of the *α* phase. Similarly, *H*_A,*γ*_, *S*_A,*γ*_, *E*_A,*γ*_, *K*_A,*γ*_ and *pV*_A,*γ*_ are the enthalpy per unit mole, entropy per unit mole, potential energy, kinetic energy and mechanical energy of the *γ* phase. Here *V*_*α*_ and *V*_*γ*_ denote the spatial regions occupied by the *α* and *γ* phases, respectively. For convenience we use the generic phase index *i* ∈ {*α*, *γ*} when writing statements that apply to either phase (*e.g. H*_A,*i*_, *S*_A,*i*_, *ρ*_*n*,*i*_). Physically, *G*_*i*,A_(**r**) is the Gibbs free energy density per unit volume. Since *H*_A,*i*_ and *S*_A,*i*_ are expressed per mole, multiplying them by the local density field *ρ*_*n*,*i*_(**r**) converts the expression into a volumetric free energy density. In this work, *ρ*_*n*,*i*_(**r**) is a coarse-grained density field that bridges the molar bulk thermodynamic quantities and a spatially resolved interfacial description.

**Fig. 1:**
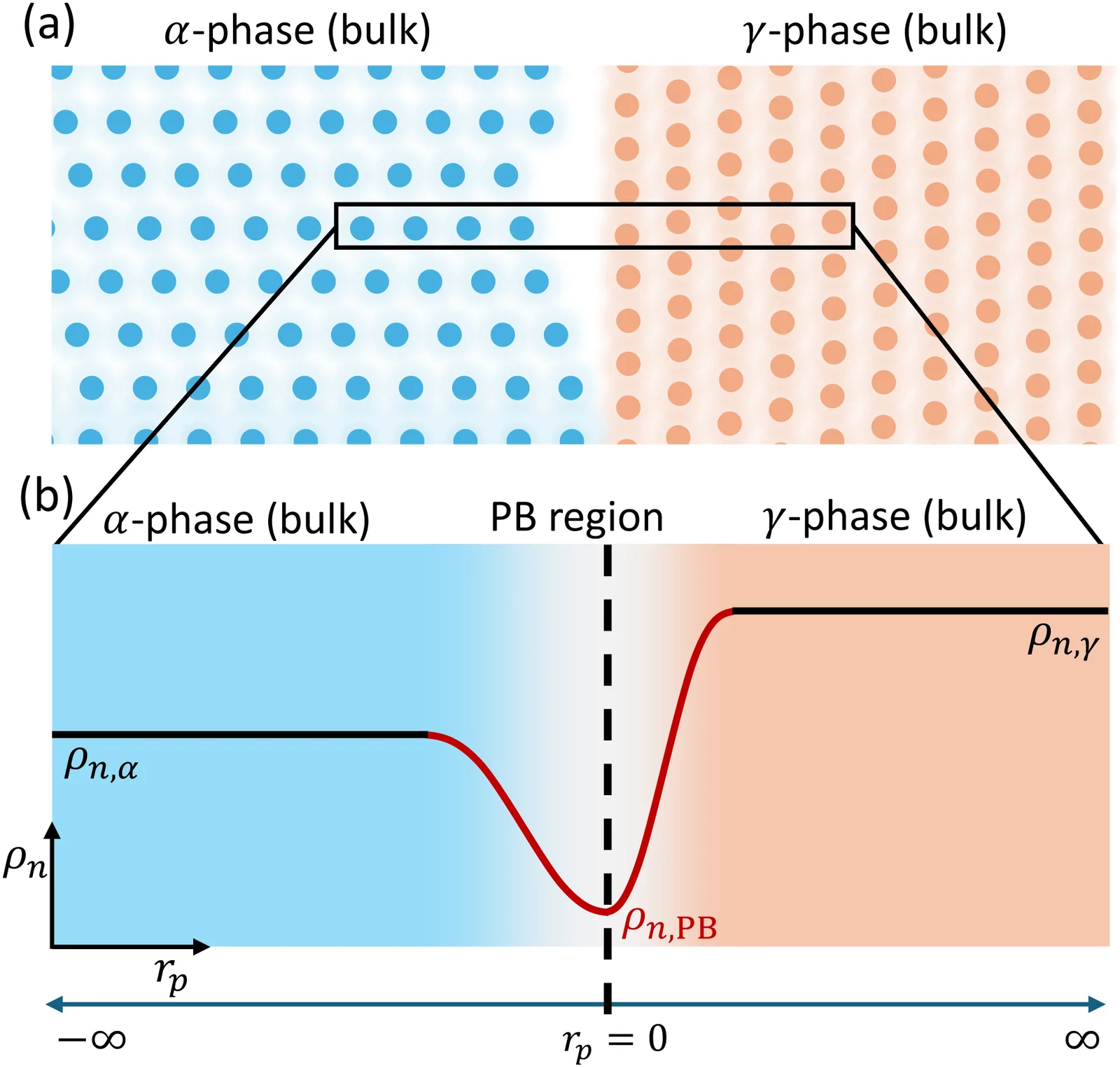
Schematic diagram of the problem. (a) Schematic illustration of the PB between *α* and *γ* bulk phases. (b) Continuous density field description with *ρ*_*n*,PB_ < *ρ*_*n*,*α*_ < *ρ*_*n*,*γ*_, across the *α* bulk phase, *γ* bulk phase and the PB region. *ρ*_*n*,PB_ is the density at the PB. The normal coordinate is *r*_p_, with PB at *r*_p_ = 0, *α*: *r*_p_ < 0 and *γ*: *r*_p_ > 0.

The potential energy can be written as a function of local density and the non-local density gradients,^25^ in the present study. We contemplate a flat PB separating infinitely extended homogeneous phases *α* and *γ*. The symmetry breaks only normal to the PB plane. We therefore introduce a one-dimensional coordinate normal to the PB, *r*_p_: = **r**·**p̂**, where **p̂** is the unit normal to the PB plane pointing from *α* to *γ*. Thus, *r*_p_ < 0 in *α* and *r*_p_ > 0 in *γ* as illustrated in Fig. 1(b). Note that *r*_p_ is a defined coordinate along **p̂** and not a radial distance. In general, with a local orthogonal frame {**p̂**,**t̂**1,**t̂**2} at the PB, *ρ*_*n*,*i*_ = *ρ*_*n*,*i*_(*r*_p_,*r*_t1_,*r*_t2_). For a planar PB, the in-plane dependence vanishes due to symmetry, giving *ρ*_*n*,*i*_ = *ρ*_*n*,*i*_(*r*_p_). We take |*r*_p_| as the geometric distance from a field point at position *r*_p_ to the PB plane at *r*_p_ = 0. The local potential energy density *E*_*i*_(*r*_p_) in each phase is given as2.2
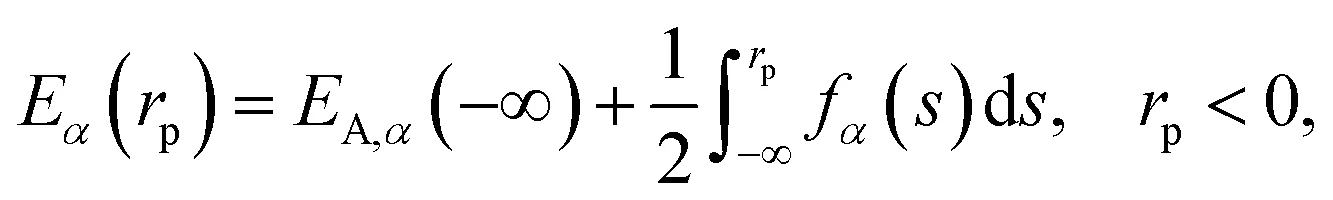
2.3
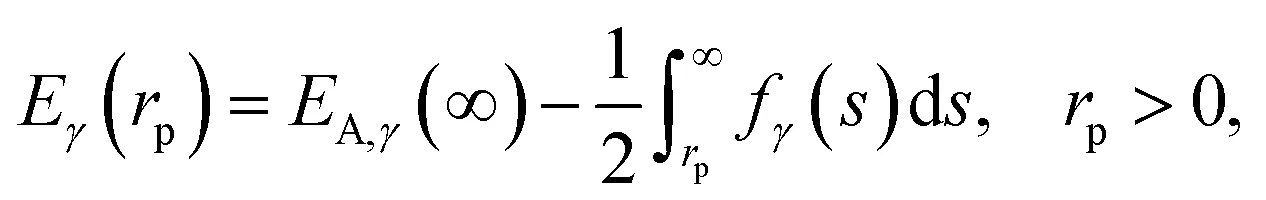
where *E*_A,*α*_(−∞) is the potential energy inside the homogeneous *α* phase at *r*_p_ = −∞ and *E*_A,*γ*_(∞) is the potential energy inside the homogeneous *γ* phase at *r*_p_ = ∞. The force densities *f*_*α*_(*r*_p_) and *f*_*γ*_(*r*_p_) describe a one-dimensional force-to-energy reconstruction: the local potential energy density at *r*_p_ is obtained by accumulating the work associated with the internal force density when moving from the far-bulk toward the interface. The factor 1/2 avoids double counting of pairwise interactions, since each interacting pair contributes symmetrically to the energy balance of the two points involved.

The force density at position *r*_p_ < 0 (on the *α* side) comprises three contributions: (i) *α*–*α* interactions with material points in *α* to the left of *r*_p_ (*f*_*α*,1_), (ii) *α*–*α* interactions with points in *α* between *r*_p_ and the PB plane (*f*_*α*,2_), and (iii) the cross-phase *α*–*γ* interaction (*f*_*α*,3_), *i.e.* the influence of the *γ* side on the field point in *α*, which becomes active only when the interaction range reaches across the PB. This decomposition is illustrated schematically in Fig. 2(a) and the force density at position *r*_p_ < 0 is written as2.4
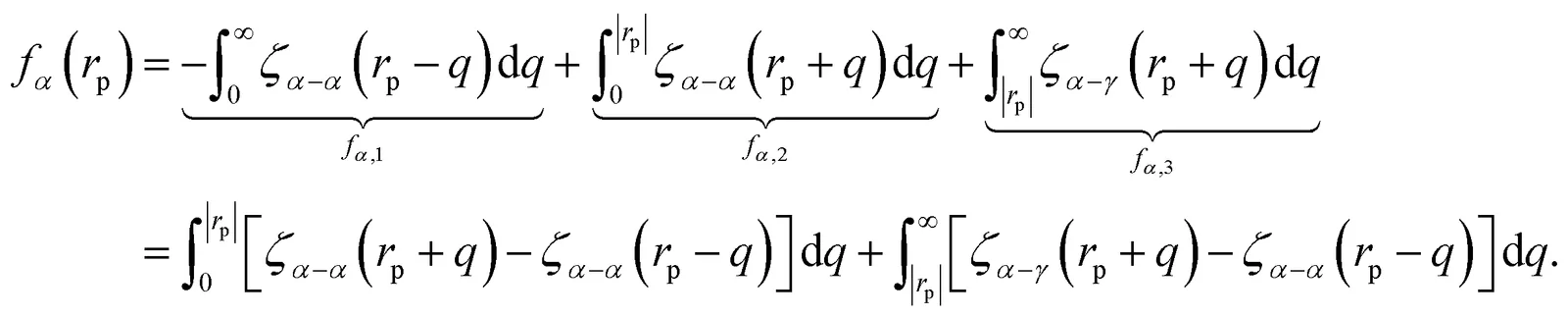
Here *q* ≥ 0 denotes the separation distance from the field point *r*_p_ along the PB normal. The quantities *ζ*_*α*–*α*_(*r*_p_ ± *q*) and *ζ*_*α*−*γ*_(*r*_p_ + *q*) represent the pairwise force density contributions at *r*_p_ due to interactions with points sampled at *r*_p_ ± *q* in the *α* phase and at *r*_p_ + *q* in the *γ* phase, respectively. In the present development, we consider a simple functional form presented in terms of the material constant *λ*_*i*−*j*_, *n*(·) the number of atoms and *z* being typically a large exponent, as shown in eqn (2.5) and (2.6), inspired from interatomic force relations in the absence of long–range interactions. For a planar PB, the force components parallel to it cancel due to symmetry. So, *ψ*_*i*−*j*_(*q*) is the effective kernel that already absorbs the integration over the PB parallel directions.2.5
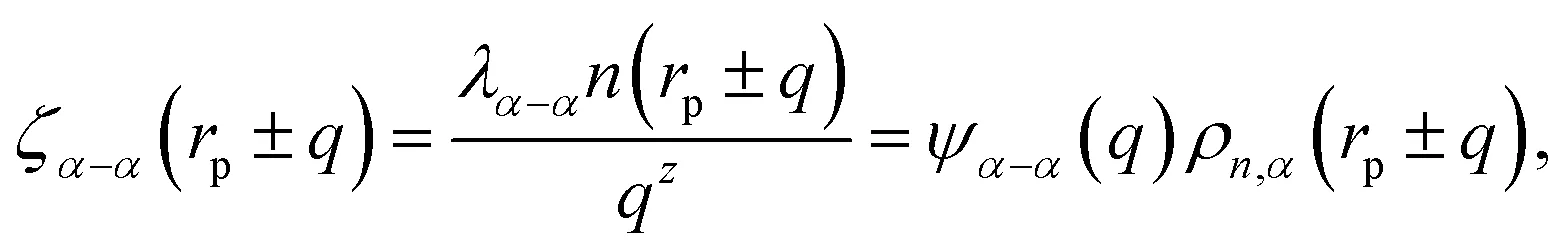
2.6
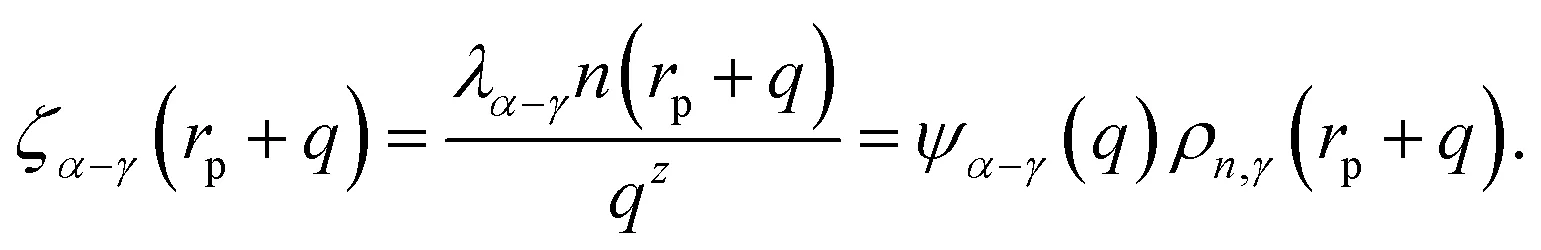
In eqn (2.5) and (2.6), the mesoscale atomic density field over a characteristic coarse-graining volume *V*_L_ is *ρ*_*n*_(·) = *n*(·)/(*N*_A_*V*_L_), with *N*_A_ the Avogadro number. Thus, *ρ*_*n*,*i*_ is a molar density in mol m^−3^. The pairwise force kernels 
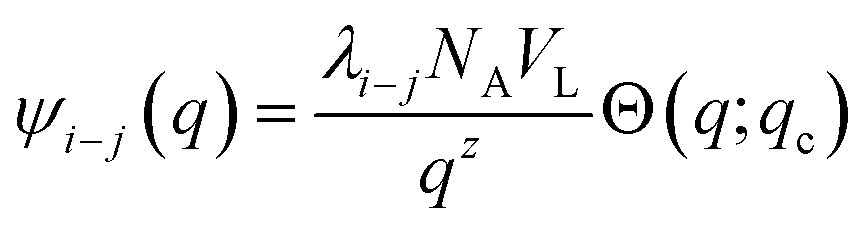
 depend on the separation *q* ≥ 0, where *Θ*(*q*; *q*_c_) denotes a cut-off at the finite interaction range *q*_c_, beyond which the interaction is negligible. These kernels are coarse-grained force kernels and are not interatomic pair potentials. They diverge as *q* → 0^+^, and the *q*_c_ ensures that all kernel moments introduced in the present study exist. This single branch form of the kernels used here is a minimal choice, and adopted for computational efficiency. A kernel consisting of repulsive and attractive branches, like the effective potentials discussed in ref. 16, is equally valid, as *ψ*_*i*−*j*_(*q*) enters the subsequent parts of the derivations only *via* its moments. The incorporation of the full interatomic potentials into the DPF free energy functional is demonstrated in.^56,57^ Also, the integral of *f*_*i*_ in eqn (2.2) and (2.3) reconstructs the local potential energy density and is not a force. Hence, the sign of *λ*_*i*−*j*_ does not follow the standard convention for interatomic potentials, rather it follows the requirements of the model. Higher-order dependencies of the interaction forces on the atomic density are neglected and only the first-order approximation in eqn (2.5) and (2.6) is retained. Thus, substituting eqn (2.5), (2.6) and (2.4) we obtain2.7
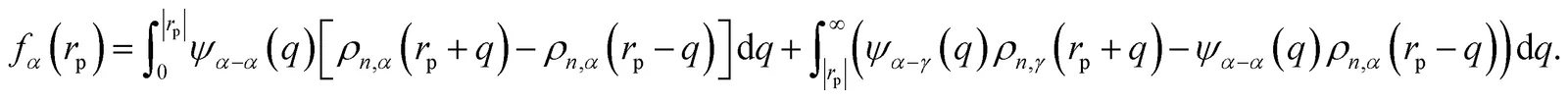


**Fig. 2:**
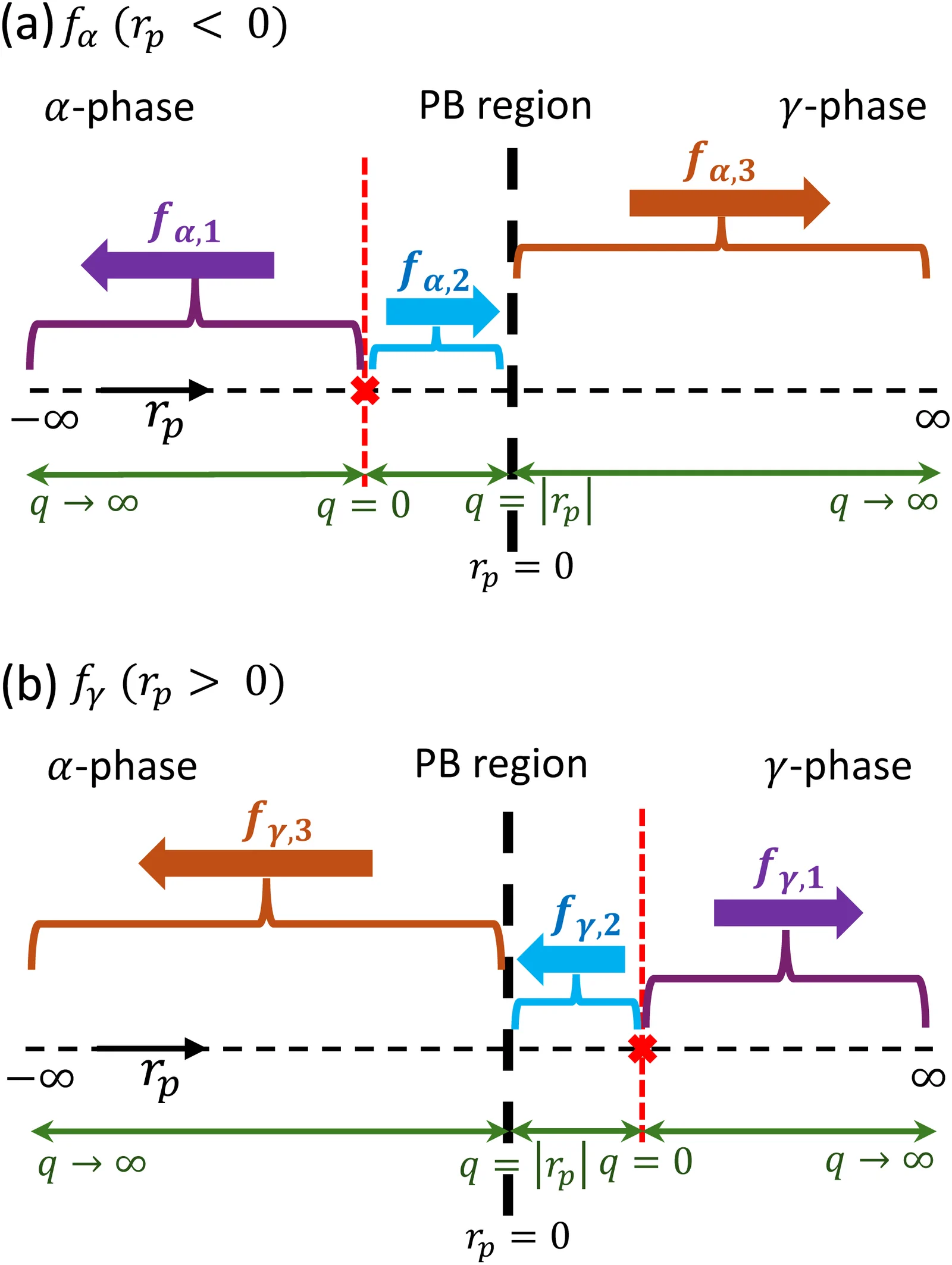
Schematic decomposition of the force density contributions at a field point *r*_p_ (marked by a red ×). For *r*_p_ < 0 (panel a), the net force density *f*_*α*_(*r*_p_) is written as the sum of three contribution blocks labeled *f*_*α*,1_, *f*_*α*,2_, and *f*_*α*,3_, corresponding to (i) same-phase interactions sampling points to the left of *r*_p_, (ii) same-phase interactions sampling points between *r*_p_ and the PB plane, and (iii) cross-PB interactions sampling points from the *γ* side. For *r*_p_ > 0 (panel b), the force density *f*_*γ*_(*r*_p_) is decomposed analogously into *f*_*γ*,1_, *f*_*γ*,2_, and *f*_*γ*,3_, representing (i) same-phase interactions sampling points to the right of *r*_p_, (ii) same-phase interactions sampling points between *r*_p_ and the PB plane, and (iii) cross-PB interactions sampling points from the *α* side. These labels are used only for illustration.

Similarly, the force density at position *r*_p_ > 0 (on the *γ* side) is written as2.8
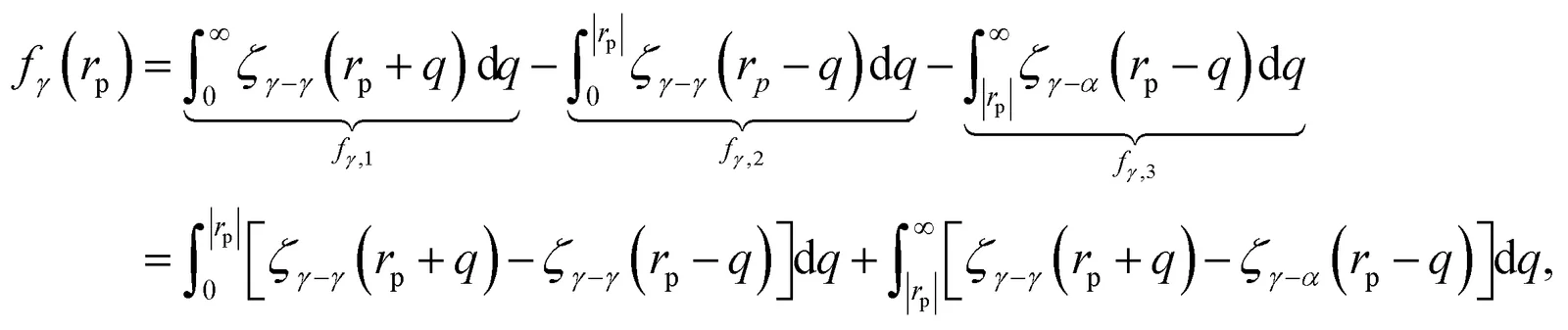
with kernel representations.2.9
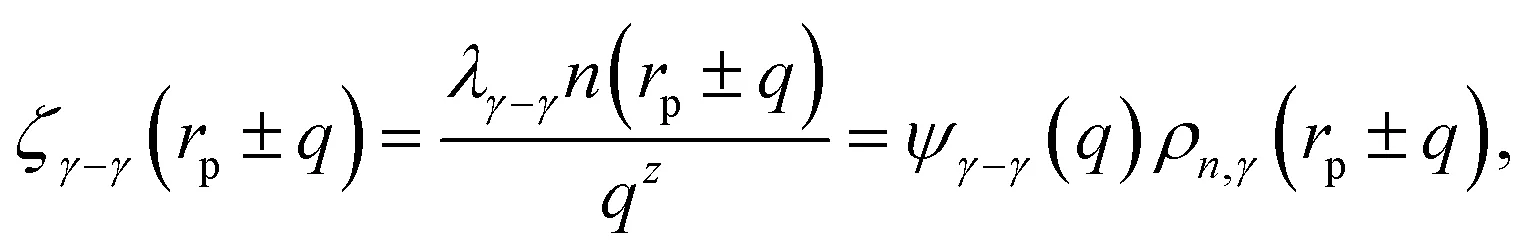
2.10
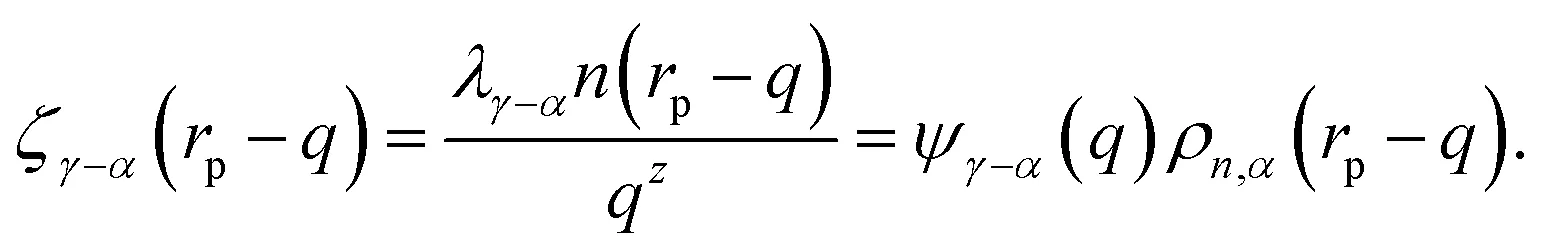


Substituting the above kernel forms gives the force density at position *r*_p_ > 0 as2.11
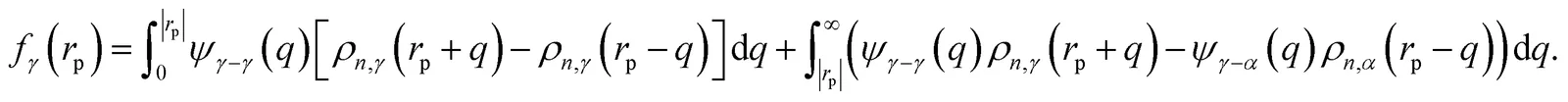


The structure of eqn (2.7) reflects: (i) a symmetric *α*–*α* contribution that would be present even in a homogeneous phase, and (ii) an additional *α*–*γ* contribution that becomes active only when the interaction range reaches across the PB. Similarly, the structure of eqn (2.11) reflects symmetric *γ*–*γ* contribution present in the homogeneous *γ* phase, and an additional *γ*–*α* contribution that becomes active only when the interaction range reaches across the PB.

Written in terms of the geometric distance |*r*_p_|, the force densities eqn (2.7) and (2.11) involve integration limits that depend explicitly on the field point *r*_p_. This is inconvenient because it masks the separation between (i) a bulk response with constant coefficients and (ii) an interface-specific correction that is activated only when the interaction range reaches across the PB. To obtain a form suitable for systematic analysis, we therefore rewrite the integrals using Heaviside functions and insert local Taylor expansions of *ρ*_*n*,*i*_(*r*_p_ ± *q*) about the field point. The complete algebra is provided in Appendix A.1.

The outcome is a structurally simple representation on each side:2.12
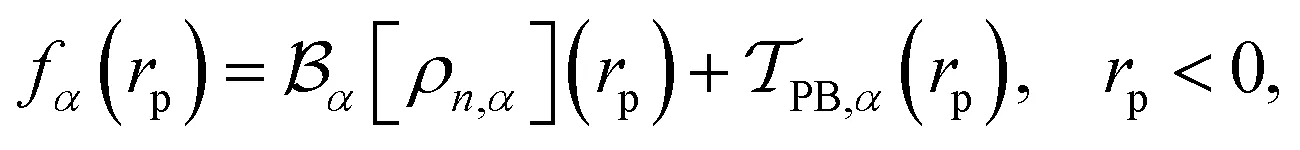
2.13
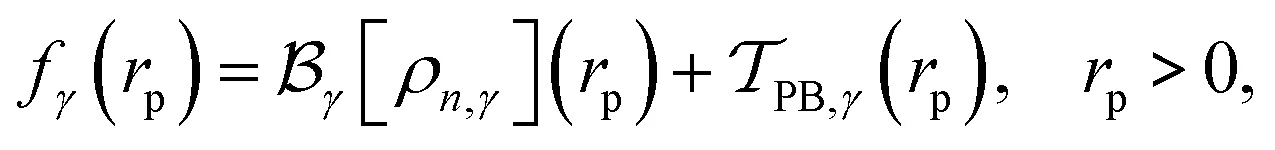


Eqn (2.12) and (2.13) hold for *r*_p_ ≠ 0 and are interpreted in the one-sided sense at the PB plane, *r*_p_ → 0^±^. Here *i*, *j* ∈ {*α*, *γ*} (with *i* and *j* not necessarily distinct). 
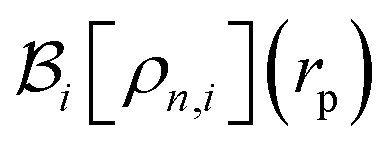
, defined in eqn (A.1.18) and (A.1.30), is the constant-coefficient bulk operator expressed in terms of kernel moments *K*_*n*_^*ij*^, which is defined in eqn (A.1.4). 
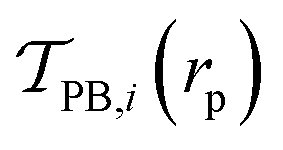
, defined in eqn (A.1.25) and (A.1.44), collects the Heaviside-channel terms associated with cross-boundary interactions. In the sharp-interface limit, 
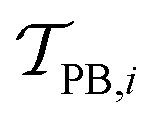
 becomes localized at *r*_p_ = 0 (it can be represented as a distribution supported at the PB and depends only on the one-sided density slopes at 0^±^). No separate force value is assigned at *r*_p_ = 0: the one-sided limits of 
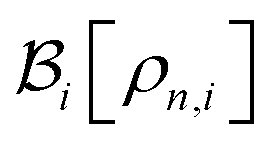
 together with the localized traction 
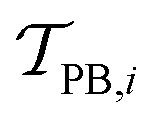
 fully specify the force density about the plane, while the interfacial density itself is set by the continuity condition introduced in Section 2.2.

### 2.2. Finite-width PB approximation

For numerical use and for parameter identification it is convenient to cast this formally sharp traction into a finite-width approximation in which the non-bulk influence of the PB is confined to thin layers of thickness *η*_*α*_ and *η*_*γ*_ on the *α* and *γ* sides, respectively-hereafter referred to as the PB half-widths on the *α* and *γ* sides.

Far from the PB, the force density must reduce to a bulk operator with constant coefficients (set by kernel moments), while close to the PB we retain only the leading cross-boundary correction and localize it inside narrow windows. Within the PB layers we approximate the density by first-order one-sided PB slopes,2.14
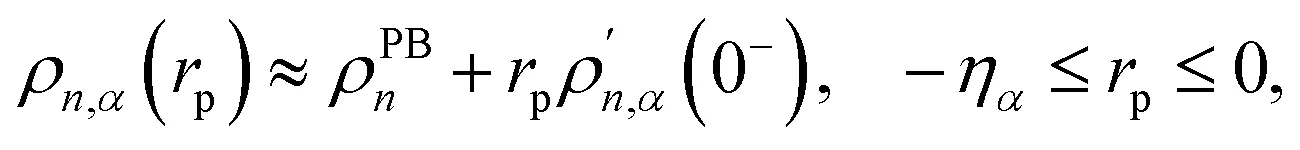
2.15
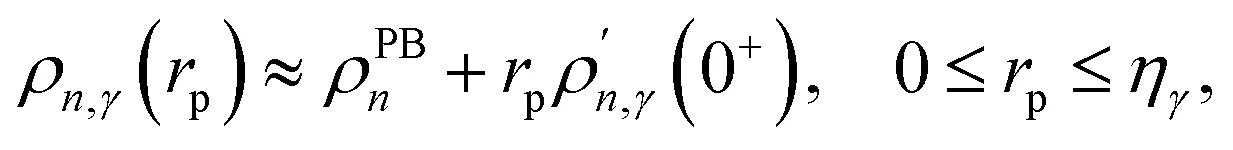
with a common interfacial value *ρ*^PB^_*n*_≔ *ρ*_*n*,*α*_(0^−^) = *ρ*_*n*,*γ*_(0^+^).

We use hat-shaped window functions supported on the PB layers,2.16
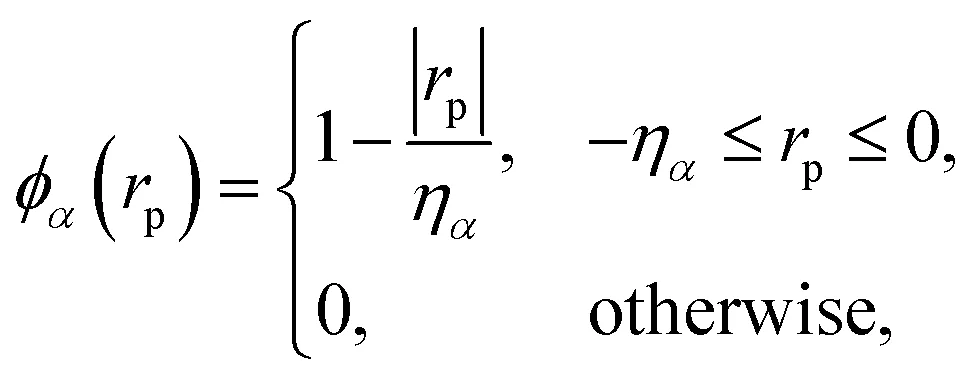
2.17
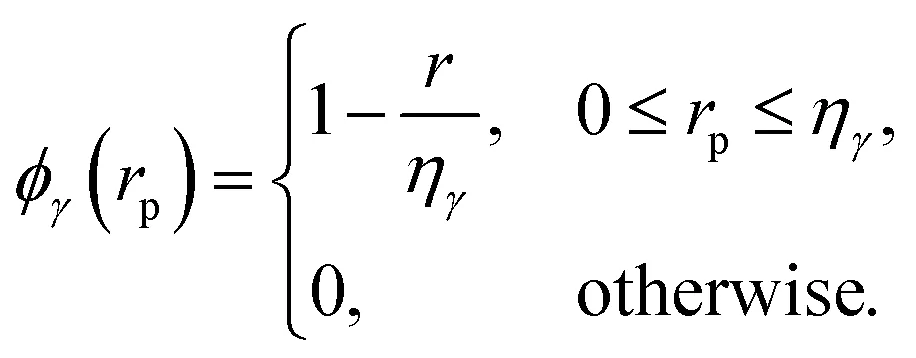


With these windows, we approximate the force densities as2.18
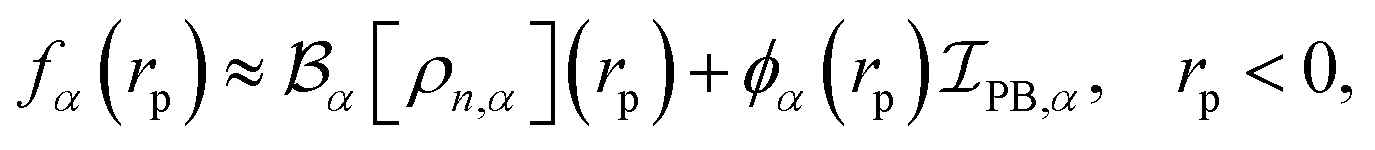
2.19
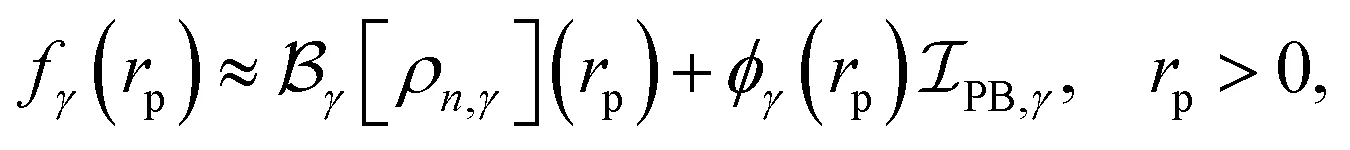
where 
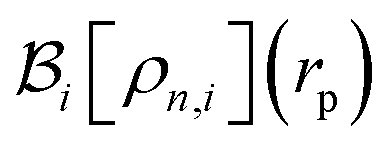
 is the constant-coefficient bulk operator obtained from kernel moments, and 
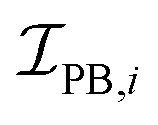
 are window-averaged amplitudes capturing the leading cross-boundary influence.

Retaining only the leading (first-order) dependence gives2.20
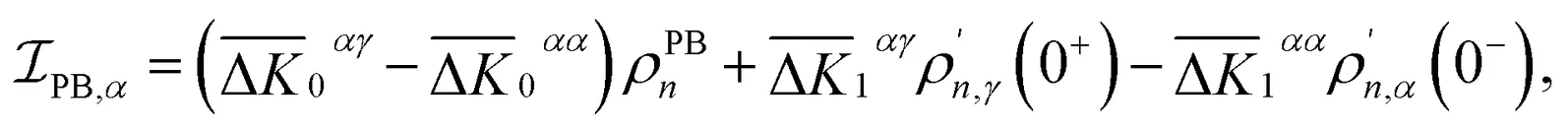
2.21
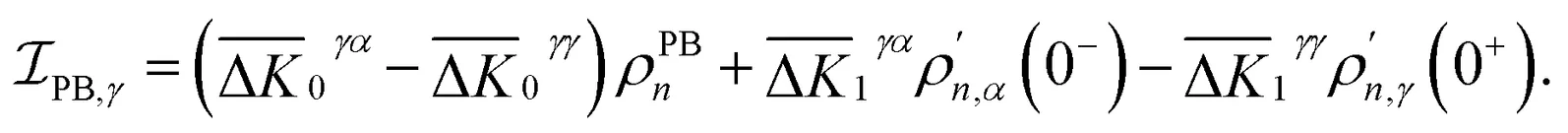
Here 
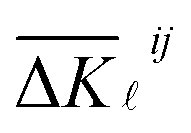
 are window-averaged cap moments defined in Appendix A.2, eqn (A.2.2) and (A.2.4). Retaining only *ℓ* = 0, 1 corresponds to keeping the leading dependence of the PB correction on the interfacial value *ρ*^PB^_*n*_ and the one-sided slopes at *r* = 0^±^, while higher-order terms are neglected.

In the far-bulk regions (|*r*_p_| ≥ *η*^*i*^) the localization functions vanish and 
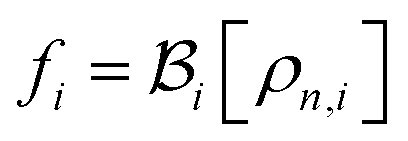
. In the sharp limit *η*_*α*_, *η*_*γ*_ → 0, the windowed correction collapses toward the distributional traction derived in Appendix A.1.

### 2.3. Gibbs free energy functional

The finite-width approximation provides closed expressions for the force densities on each side of the PB in terms of a bulk operator 
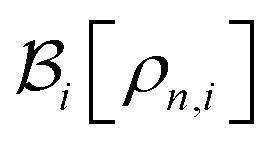
 and a localized PB correction confined to layers of thickness *η*_*α*_ and *η*_*γ*_. We reconstruct a local (gradient-expanded) Gibbs free energy functional from these forces.

We truncate the *α*-side bulk operator at fifth order,2.22
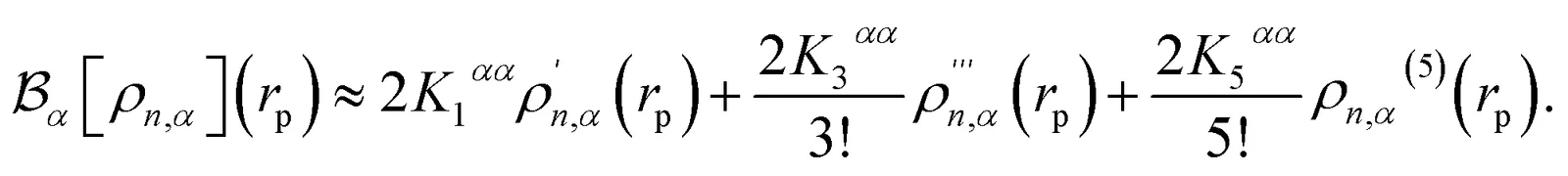


Inserting the eqn (2.18) into eqn (2.2) and using *ϕ*_*α*_(*r*_p_) = 0 for *r*_p_ ≤ −*η*_*α*_ gives,2.23
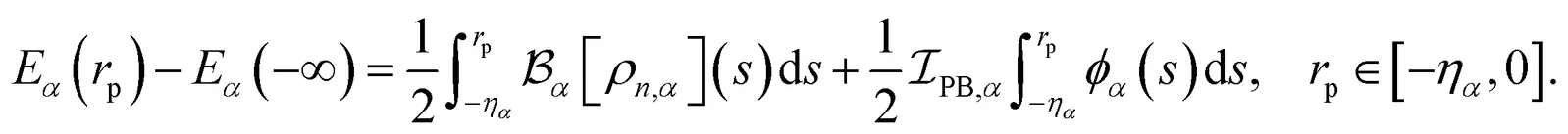
The bulk-operator integral evaluates term-by-term:2.24
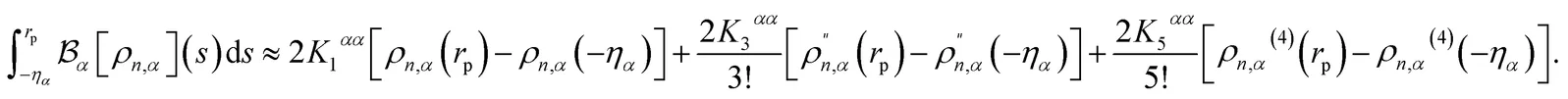


At *r*_p_ = −*η*_*α*_ the profile is assumed close to bulk so that higher derivatives are negligible and remaining reference terms are absorbed into *E*_*α*_(−∞). This gives the local gradient-expanded form2.25
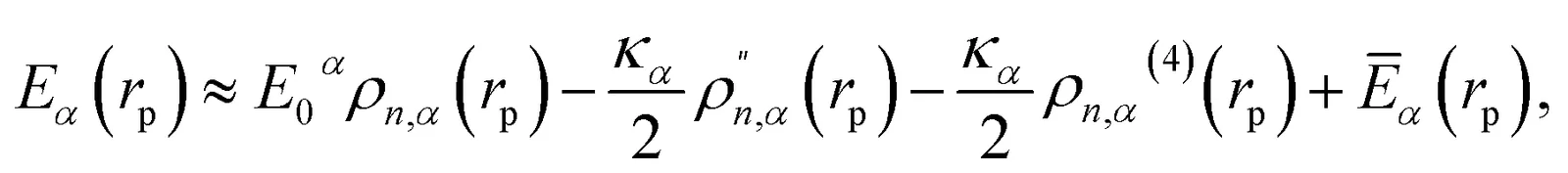
with2.26
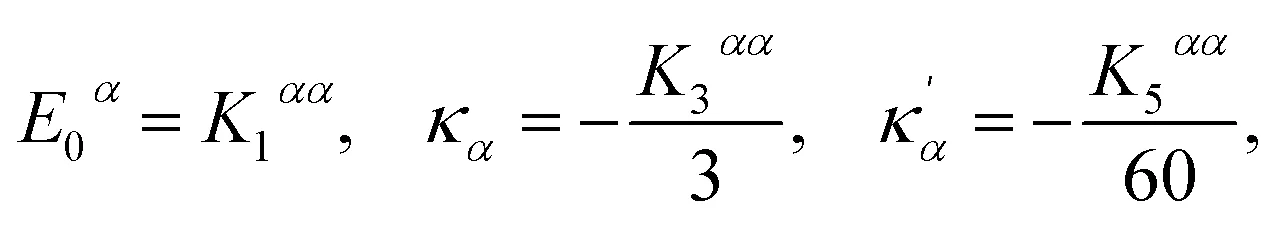
and2.27
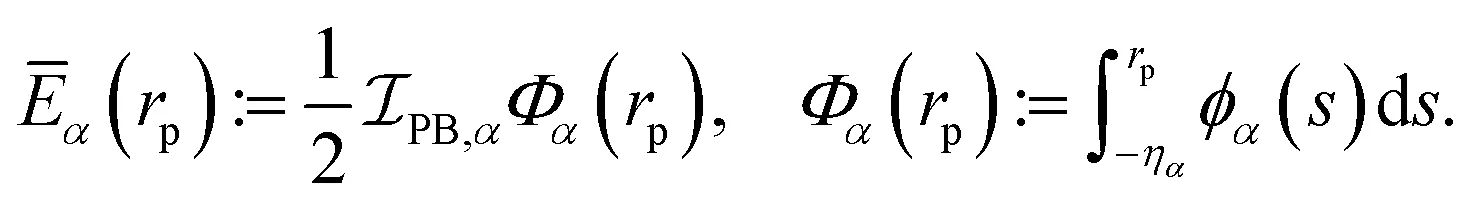
The coefficients *κ*_*i*_ and 
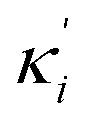
 are set by the kernel moments *K*_3_^*ii*^ and *K*_5_^*ii*^ up to the sign of the underlying force kernel *ψ*_*i*−*i*_(*q*). As *q* ≥ 0, *q*^*n*^ is positive for all *n* and the sign of each moment *K*_*n*_^*ij*^ comes directly from that of *ψ*_*i*−*j*_(*q*). The DPF model requires *E*^B^_*A*,*i*_ < 0, *κ*_*A*,*i*_ > 0 and 
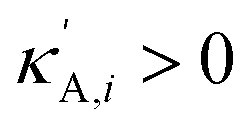
, thus results in a condition for same phase kernels *ψ*_*i*−*i*_(*q*) < 0 and hence *λ*_*i*−*i*_ < 0. The cross phase kernels *ψ*_*i*−*j*_ enter only through the traction contribution, hence their sign and magnitude are calibrated independently against the coarse-grained density profile, presented in Section 3. In practice, *κ*_*A*,*i*_ and 
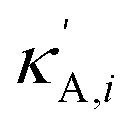
 are treated as calibratable coefficients. For the hat-shaped window *ϕ*_*α*_(*r*_p_) = 1 − |*r*_p_|/*η*_*α*_ on [−*η*_*α*_, 0), *Φ*_*α*_(*r*_p_) is a quadratic shape function non-zero only inside the PB region.

The local Gibbs free energy density on the *α* side is2.28
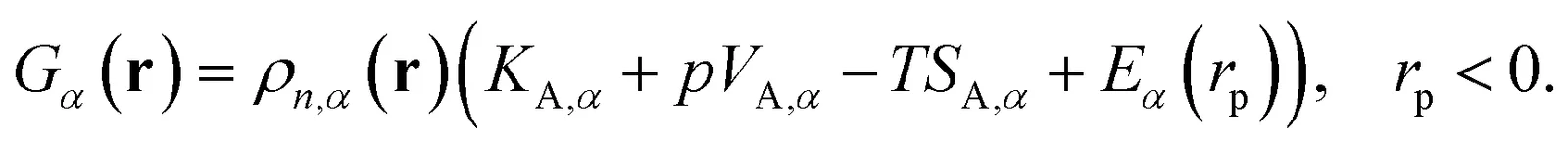
Substituting eqn (2.25) in eqn (2.28) and using the following integration by parts identities. For a planar PB, 
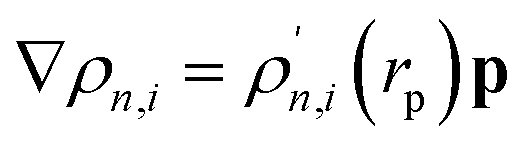
 and 
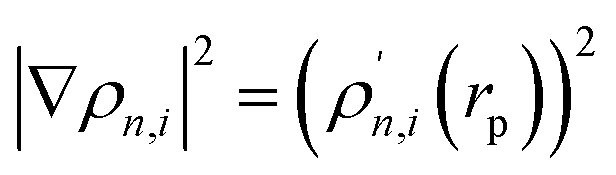
,
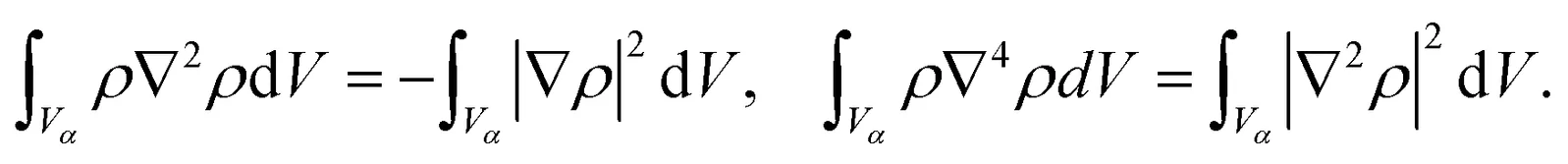
The boundary terms vanish due to the imposed far-field bulk conditions and the PB-node condition. The Gibbs free energy functional is written as
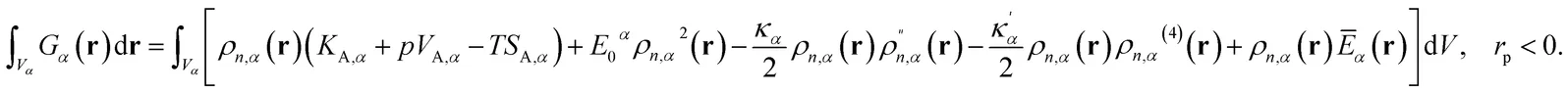
From here, we obtain the Gibbs free energy density in the local + gradient + traction form2.29
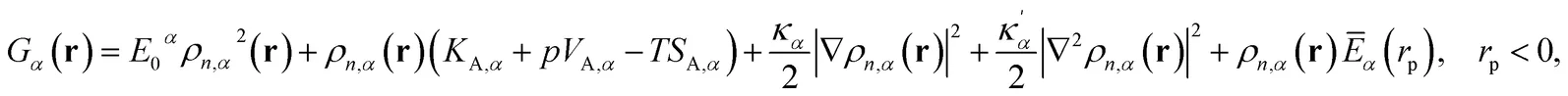
with *Ē*_*α*_(*r*_p_) given as follows from eqn (2.20), (A.2.2) and (A.2.4),
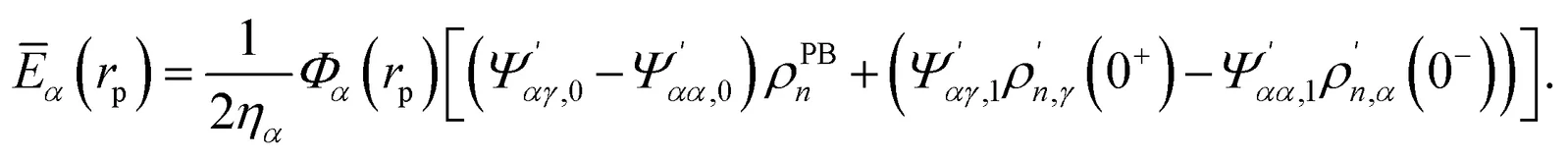


Similarly, on the *γ* side, inserting eqn (2.19) into eqn (2.3) and repeating the steps above yields
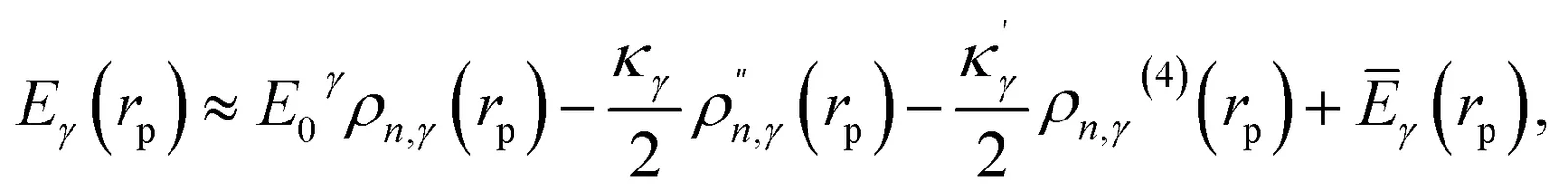
with
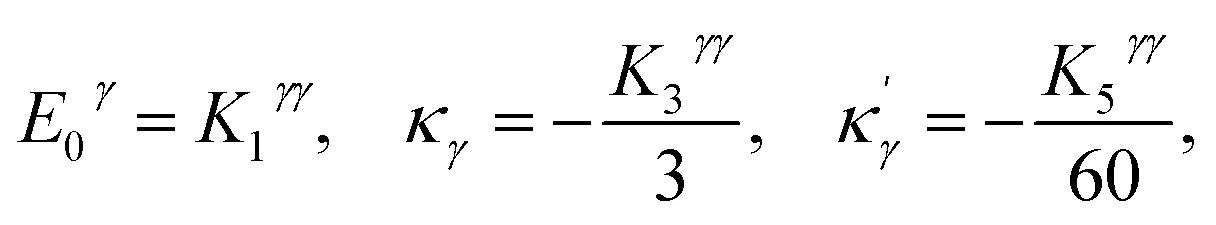
and the localized PB contribution
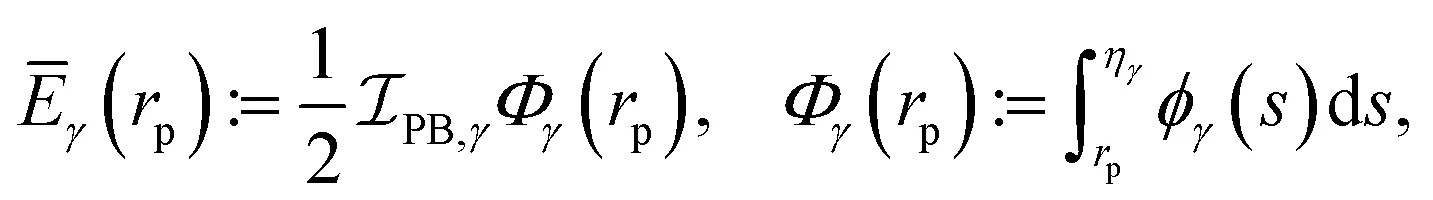
which is supported only on (0, *η*_*γ*_].

The *γ*-side Gibbs free energy density is2.30
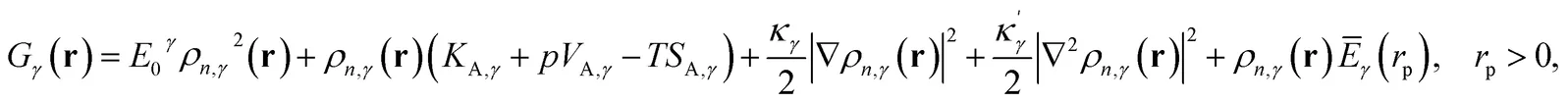
with *Ē*_*γ*_(*r*_p_) given from eqn (2.21), (A.2.2) and (A.2.4),
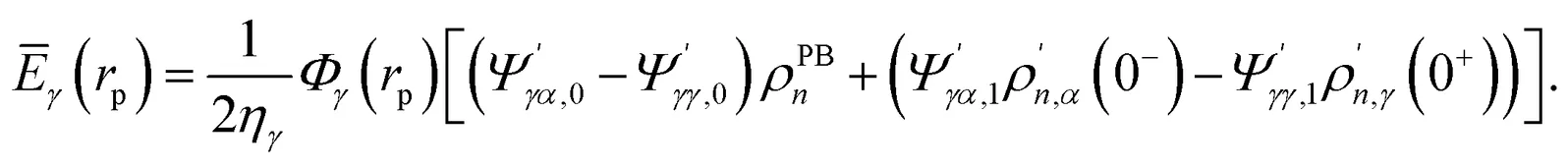
For numerical implementation and direct comparison of PB profiles across phases, we introduce a dimensionless relative density field defined as *ρ*(**r**)**≔***ρ*_*n*,*i*_(**r**)/*ρ*_ref_, similar to the density-based GB formulation.^1^ This normalization removes the dependence on the absolute density scale, allowing the bulk and interfacial contributions in the functional to be compared on a consistent dimensionless scale.

In the present work, we choose the reference density as the largest bulk density among the coexisting phases *ρ*_ref_≔ max(*ρ*^B^_*n*,*α*_,*ρ*^B^_*n*,*γ*_) with superscript B standing for bulk phase (so that *ρ* ≤ 1), we rewrite the eqn (2.29) and (2.30) in terms of relative density *ρ* as follows2.31
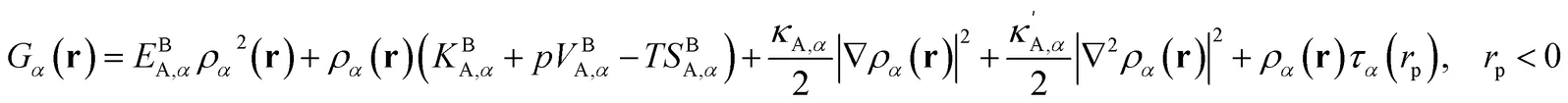
with
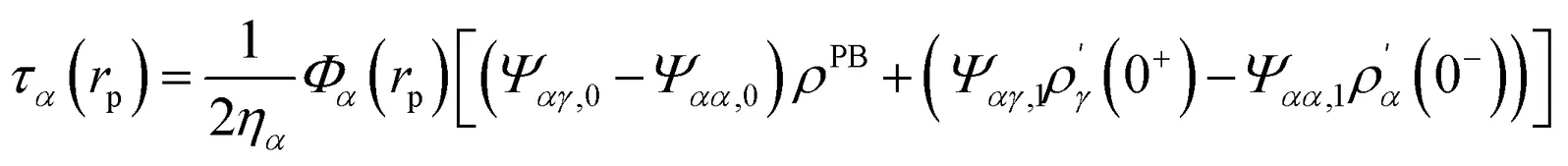
and2.32
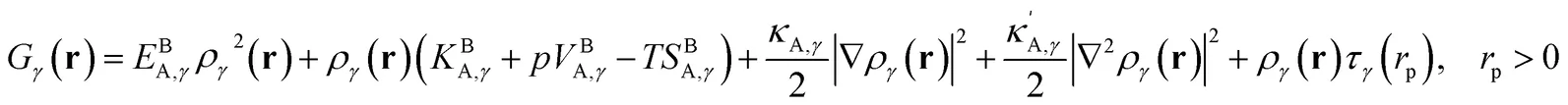
with
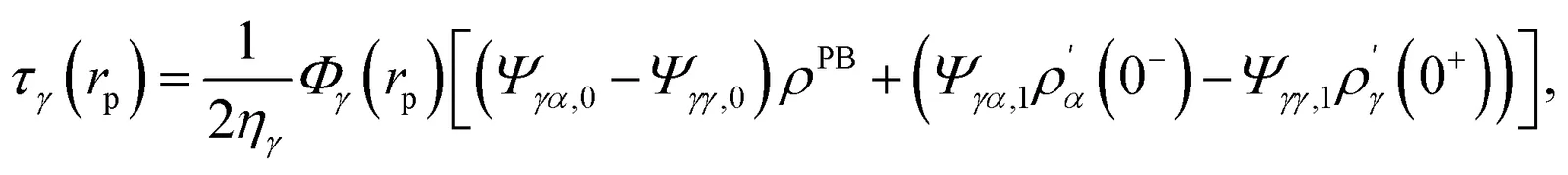
where *ρ*_*i*_(*r*_p_) = *ρ*_*n*,*i*_(*r*_p_)/*ρ*_ref_, *E*^B^_A,*i*_ = *E*^*i*^_0_(*ρ*_ref_)^2^*v*_m_^*i*^, *K*^B^_A,*i*_ = *K*_*i*_*ρ*_ref_*v*_m_^*i*^, *V*^B^_A,*i*_ = *V*_*i*_*ρ*_ref_*v*_m_^*i*^, *S*^B^_A,*i*_ = *S*_*i*_*ρ*_ref_*v*_m_^*i*^, *κ*_A,*i*_ = *κ*_*i*_(*ρ*_ref_)^2^*v*_m_^*i*^, 
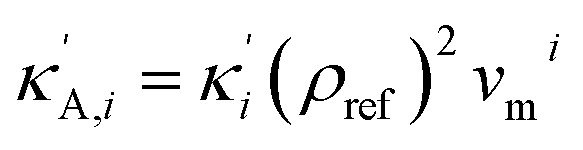
, *ρ*^PB^ = *ρ*^PB^_*n*_/*ρ*_ref_, 
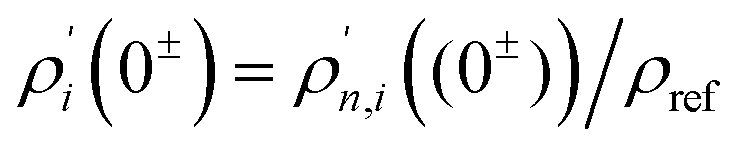
, 
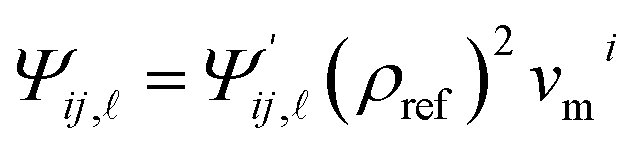
 for *ℓ* = 0, 1 and *i*, *j* ∈ {*α*, *γ*}. With these definitions, the coefficients are expressed per mole. Hence, eqn (2.31) and (2.32) are molar free energies.

The total Gibbs free energy is obtained by substituting eqn (2.31) and (2.32) in eqn (2.1) and performing integration over the respective regions. The PB energy (excess free energy) density is the sum of the excess free energy densities of both *α*- and *γ*-sides. The PB energy density is given as2.33
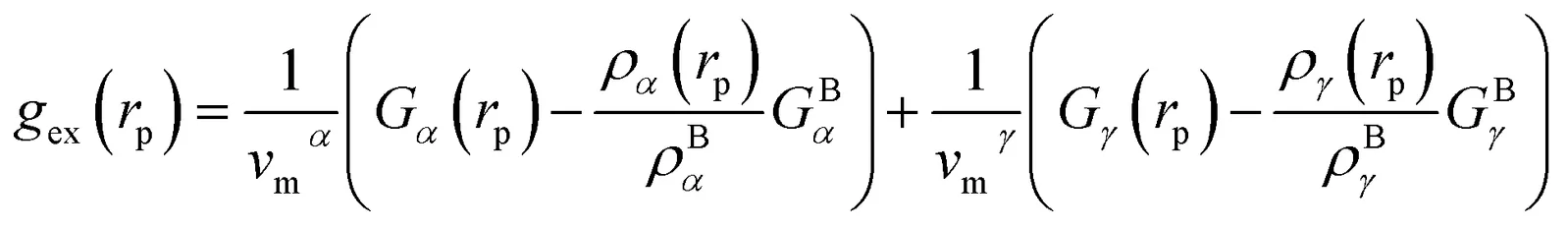
and expanded as2.34
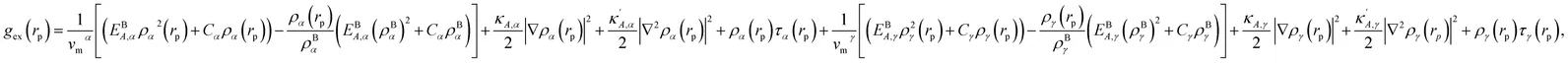
where *C*_*i*_≔*K*^B^_*A*,*i*_ + *pV*^B^_*A*,*i*_ − *TS*^B^_*A*,*i*_ (*i* ∈ {*α*, *γ*}). Note that although the density has already been normalized as *ρ*_*i*_ = *ρ*_*n*,*i*_/*ρ*_ref_ in eqn (2.31) and (2.32), the excess free energy compares the local free energy density against the bulk value for the same amount of material, and since *G*^B^_*i*_ is defined at *ρ*_*i*_ = *ρ*^B^_*i*_, the factor *ρ*_*i*_/*ρ*^B^_*i*_ rescales it to the local density.

We obtain the excess free energy *Γ*_ex_ in the considered 1D domain as2.35
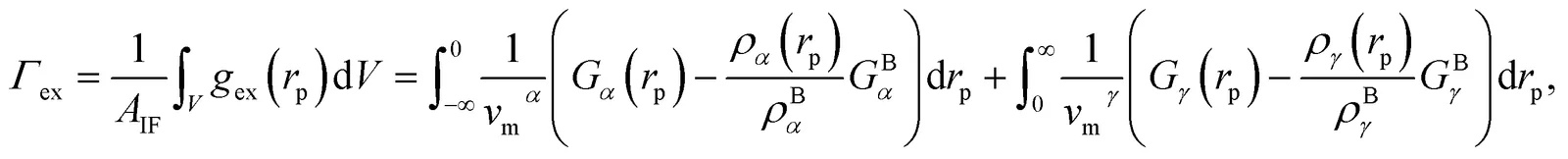
where *A*_IF_ is the interfacial area.

### 2.4. Numerical implementation and equilibrium solution

Equilibrium PB density profiles are obtained through iterative relaxation of the density fields according to the density potentials, given in eqn (2.36), derived from the one-dimensional excess free energy functional along the normal direction *r*_p_ with respect to the phase-specific relative density fields *ρ*_*α*_(*r*_p_) for *r*_p_ < 0 and *ρ*_*γ*_(*r*_p_) for *r*_p_ > 0, subject to fixed far-field bulk conditions and interfacial condition *ρ* = *ρ*^PB^ at *r*_p_ = 0. The *ρ*^PB^ enters the model as an input PB structural descriptor and is not computed. It is the quantity through which the atomistic structure of a specific PB, is carried into the continuum DPF PB functional. The density profile, the half-widths and the excess free energy are then obtained for that PB.

The computational domain is decomposed into two domains, one for the *α* phase with *r*_p_ ∈ [−*L*_*α*_, 0]. The other domain for the *γ* phase with *r*_p_ ∈ [0, *L*_*γ*_]. The interface is represented by a shared PB node at *r*_p_ = 0, for which the density is described as *ρ*(0) = *ρ*^PB^. One-sided density gradients are evaluated on the *α* and *γ* sides of this node. Here *L*_*α*_ and *L*_*γ*_ are the far-field extents of the *α*- and *γ*-side half-domains and are chosen large enough such that homogeneous bulk conditions are reached at *r*_p_ = −*L*_*α*_ and *r*_p_ = *L*_*γ*_. We discretize the *α*- and *γ*-side domains on uniform grids *N*_*α*_ and *N*_*γ*_, respectively, of equal spacing Δ*r* on both sides. The PB node is represented by a shared interfacial node, and density continuity is enforced by prescribing *ρ*_*α*_(0^−^) = *ρ*_*γ*_(0^+^) = *ρ*^PB^. The far-field bulk densities are imposed as fixed boundary conditions on the *α*- and *γ*-side domains. These boundary values remain fixed throughout the calculations.

Within each phase *i* ∈ {*α*, *γ*}, the local contribution is evaluated relative to the prescribed bulk density of each phase. The resulting local term of the density potential is, *µ*_loc,*i*_(*r*_p_) = 2 *E*^B^_*A*,*i*_(*ρ*(*r*_p_) − *ρ*^B^_*i*_), where *ρ*^B^_*i*_ denotes the prescribed bulk density of phase *i*. The local excess contribution is evaluated in the bulk referenced form and thus in the numerical implementation, *C*_*i*_ cancels when the bulk reference state is subtracted. This formulation ensures that the local contribution vanishes in the homogeneous far-field bulk region, *µ*_loc,i_ = 0 when *ρ*_*i*_ = *ρ*^B^_*i*_. It comes from the variation of the *Γ*_ex_ together with the subtraction of the bulk potential *µ*^B^_*i*_ = *E*^B^_A,*i*_*ρ*^B^_*i*_, which is a constant offset fixed by the requirement that the prescribed far-field density be a solution of the equation solved in the interior nodes. The complete derivation, together with the stability of the homogeneous bulk state, is given in Appendix A.5. The density potentials for both sides are then given as2.36
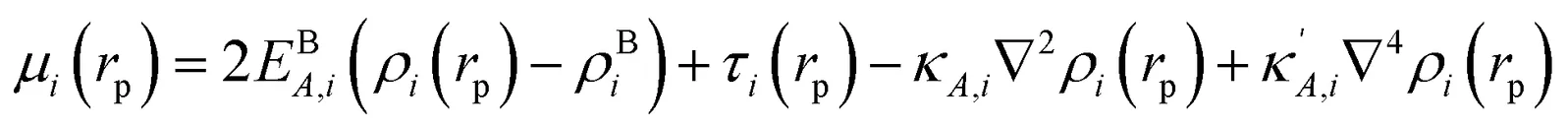
where *κ*_*A*,*i*_ and 
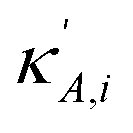
 are the second-order and fourth-order gradient regularization coefficients, respectively, and *τ*_*i*_(*r*_p_) denotes the traction contribution. The traction contribution *τ*_*i*_(*r*_p_) appearing in eqn (2.36) is the same term that appears in the free energy functional through *ρ*_*i*_*τ*_*i*_(*r*_p_) in eqn (2.31) and (2.32). It depends on the interfacial density *ρ*^PB^, the one-sided PB slopes 
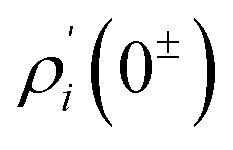
 and the PB half-widths *η*_*i*_, all of which evolve with the density profile. Accordingly, *τ*_*i*_ is treated a frozen field within each iteration of the employed relaxation scheme. At the beginning of each iteration, the current density profile is used to calculate the slopes and half-widths, from which the *τ*_*i*_(*r*_p_) is reconstructed and held fixed while the densities are updated. The terms arising from the dependence of *τ*_*i*_ on the density profile are thus ignored from the variation. To establish a fully variational form, first a smooth measure of the half-widths have to be established, unlike the threshold based extraction of *η*_*i*_.

The derivatives are discretized separately on each half-domain. In each half-domain for the interior nodes, centered finite difference scheme is used for derivative evaluations at interior nodes, while one-sided second-order accurate finite differences are employed at the PB and boundaries. At the PB, one-sided second-order accurate discretization is employed to calculate the density gradients 
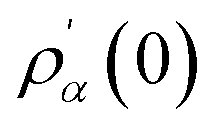
 and 
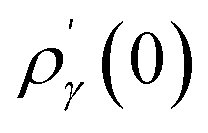
 which enter the traction term. The second-order and fourth-order derivatives at the PB are evaluated using a mirror-like curvature approximation so that the curvature conditions depend only on the density field of the respective phase side. The resulting finite-difference expressions used at the PB node are given in Appendix A.3. At the far-bulk boundaries, flat curvature is applied.

The equilibrium solution is obtained through an iterative gradient flow relaxation. At each iteration, the density profiles on the *α* and *γ* sides are updated according to2.37
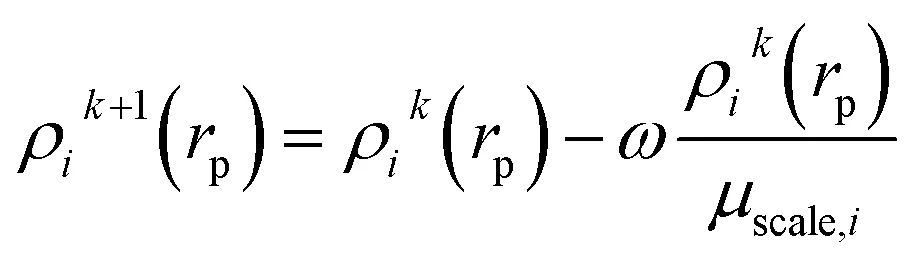
where *k* denotes the iteration count, *ω* = 10^−4^ is the relaxation factor and *µ*_scale,*i*_ is the normalization factor accounting for the relative magnitudes of the local, gradient and traction contributions. The normalization factor is chosen from the characteristic magnitudes of the individual contributions entering the density potential as 
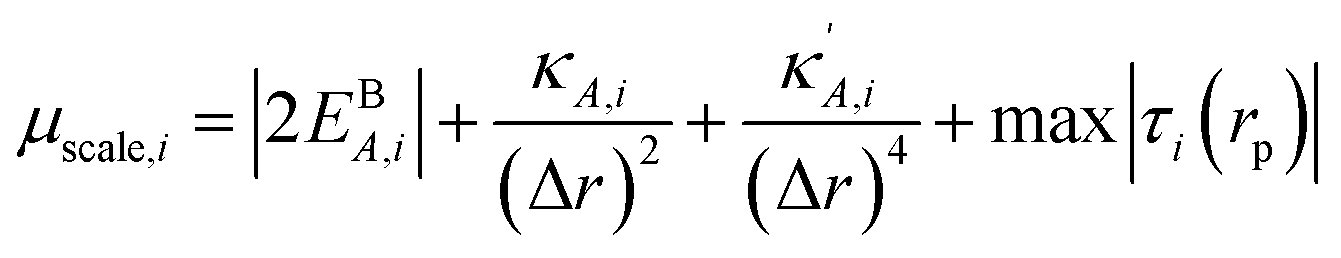
. This scaling balances the relative contributions of the local, gradient and traction terms and improves numerical stability during the relaxation process. The PB half-width *η*_*i*_ is extracted directly from the density profile as the distance from the PB node to the location at which the density reaches the corresponding bulk value within a prescribed tolerance, |*ρ*_*i*_(*r*_p_) − *ρ*^B^_*i*_| ≤ 10^−4^. The exact location is determined from the neighboring grid points for which |*ρ*_*i*_ − *ρ*^B^_*i*_| > 10^−4^ and |*ρ*_*i*_ − *ρ*^B^_*i*_| ≤ 10^−4^ and the corresponding value of *η*_*i*_ is obtained by linear interpolation between these points. During this update the boundary bulk nodes and the PB node are held fixed. The two nodes adjacent to each of the far-field boundaries and to the PB node are excluded from the relaxation, as they are required by the finite difference stencils, while the remaining interior nodes are updated. The complete scheme, including the stencils used at each node type, is given in Appendix A.3. At each iteration, the current density profile is used to determine the PB slopes and half-widths, from which the traction fields are constructed before updating the density fields. The iterative procedure is repeated until the maximum change in density between two successive iterations, evaluated over all free nodes, falls below the prescribed convergence criteria, max|*ρ*_*i*_^*k*+1^(*r*_p_) − *ρ*_*i*_^*k*^(*r*_p_)| < 10^−9^.

After convergence of the density profiles, the excess free energy density is evaluated point-wise from the converged solution as2.38
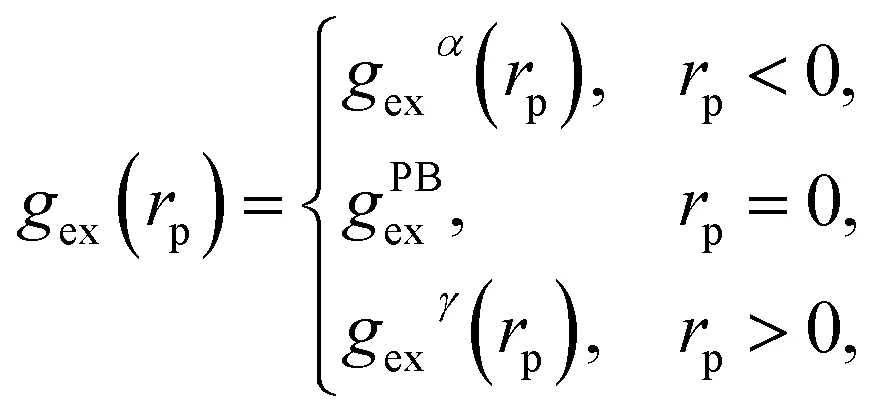
where the PB node at *r*_p_ = 0 is treated separately by averaging the limiting one-sided contributions from the two phases.

For *r*_p_ < 0 and *r*_p_ > 0, the phase-wise excess free energy densities are defined as2.39
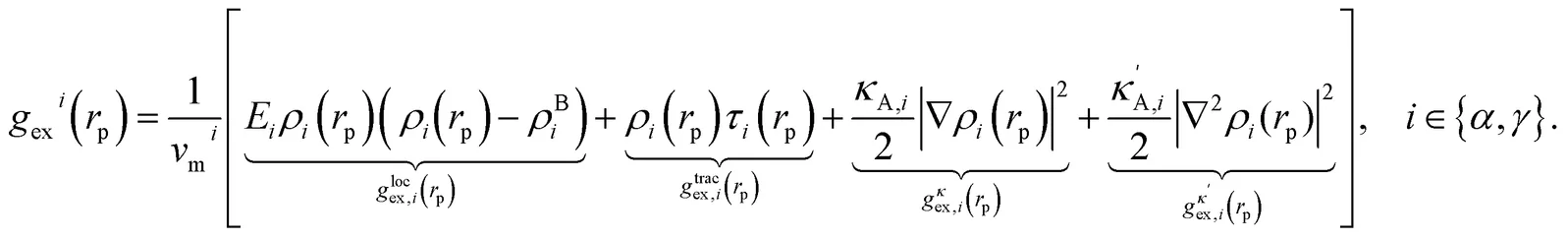
Here *v*_m_^*i*^ is the molar volume of phase *i*, and the excess is evaluated relative to the corresponding bulk reference state on each side of the PB.

At the PB node (*r*_p_ = 0), the excess free energy density is evaluated as a post-processing quantity by averaging the limiting one-sided contributions from the *α* and *γ* sides,2.40
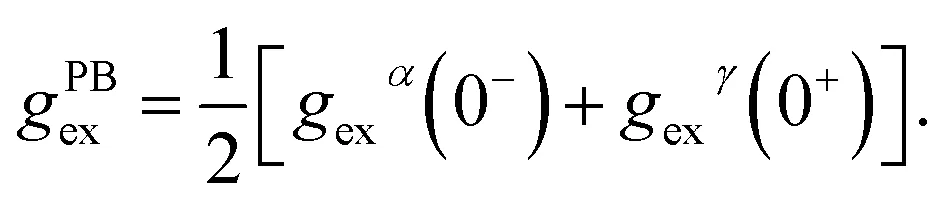


The excess free energy, the numerical realization of eqn (2.35), is then obtained by integrating the excess free energy density over the full one-dimensional domain,2.41
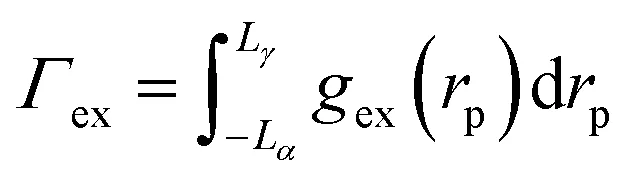
Note that since *g*_ex_ has units of energy density (J m^−3^), integration along the normal coordinate *r*_p_ yields the excess free energy per unit interfacial area (J m^−2^).

## 3. Linking the model with atomistic simulations

A key strength of the DPF framework lies in its ability to parameterize the mesoscale free energy functional for microstructural defects based on atomistic simulations. Such bridging between DPF and atomistic simulations has already been explored in earlier studies on GBs.^56,57^ In the current developments, a realistic knowledge of the atomic structural features of PBs significantly enhances the predictive capacity of the model. To this end, the atomic density at the PB plane *ρ*^PB^, as well as the full density field across the PB, are determined by establishing a connection between the discrete atomic structure and the continuous atomic density field parameter *ρ*. Following our approach previously applied to GBs,^56^ the discrete atomic density expressed as the sum of Dirac delta functions
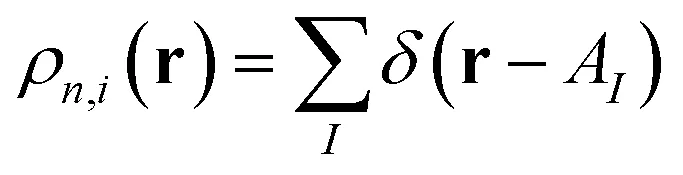
is replaced by a continuous representation using a three-dimensional Gaussian distribution. The coarse-grained atomic density is then given by3.1
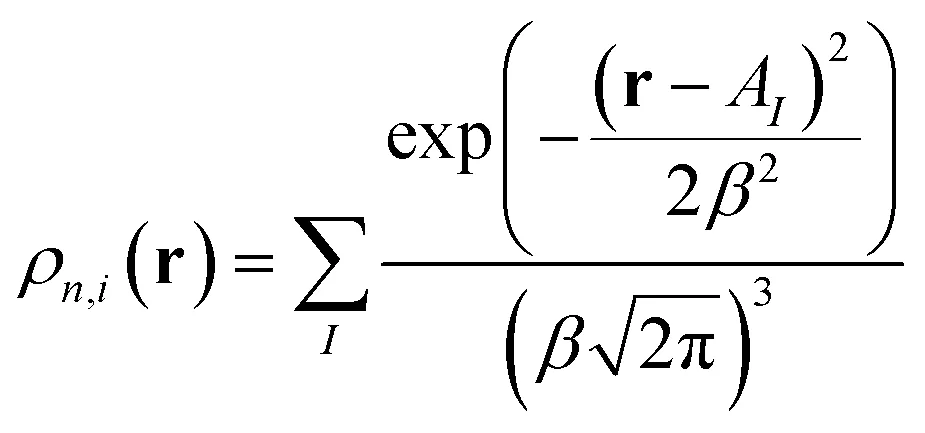
where **r** denotes the spatial coordinate in the simulation domain and *A*_*I*_ the position of atom *I*, with corresponding dimensionless atomic density field 
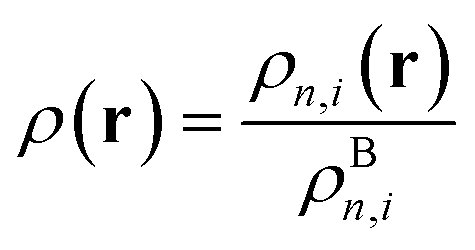
 where the atomic density of the defect-free bulk reference system is given as3.2
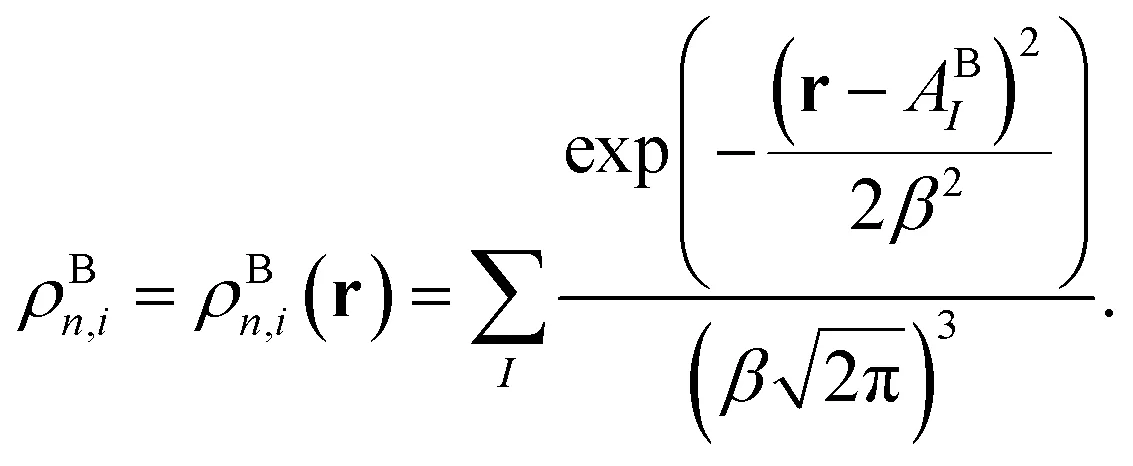
With a prescribed coarse-graining length *β*, a continuous atomic density profile across the PB can be obtained which is usually spatially smooth. The parameter *β* plays a central role, as it controls both the smoothness of the density field and the extent to which atomic-scale structural features are retained in the mesoscale free energy functional.

We consider a BCC–FCC Fe boundary. The atomistic configuration, obtained using the embedded atom method potential, is taken from Song and Hoyt.^58,59^ The orientation relationship is (110)_bcc_//(776)_fcc_ and [001]_bcc_//[1̄10]_fcc_, which is the Nishiyama–Wasserman orientation relationship tilted by 4.04° about an axis lying in the PB plane. The interface planes are thus (110)_bcc_ and (776)_fcc_, with the interface normal along [110]_bcc_. The PB is semi-coherent. The tilt introduces a regularly spaced array of structural disconnections, three per simulation cell, and misfit dislocations are present with Burgers vectors lying in the PB plane. The lattice parameters of the two phases differ by ≈22%, and the cell dimensions parallel to the PB were chosen such that the residual interface area mismatch is <0.4%.

In pure Fe the *α* ↔ *γ* transformation occurs at 1185 K, so that at 1000 K the phases are metastable. The potential used does not capture this phase transformation, instead captures a migrating PB. Eventually, the PB attains a steady velocity, with its structure becoming stationary in the frame of the moving boundary, and the density profile is extracted from this regime for our study. Since each side of the PB is referenced to its own bulk state, as given in eqn (2.33), the difference in free energy between the phases does not enter the excess free energy. Only the structural descriptors *ρ*^B^_*i*_, *ρ*^PB^ and *η*^at^_*i*_ are taken from the atomistic results. This PB density profile discussed in the present study refers to the stationary solution at 1000 K and not to the two-phase equilibrium coexistence.

Physically, the coarse-graining length can be related to the cut-off radius used in atomistic simulations, beyond which interatomic interactions are negligible.^1^ Since the cut-off radius represents the maximum interaction distance, a physically meaningful choice for *β* is on the order of the lattice constant (*a*_Fe_ ≈ 2.855 Å) in the given material. Increasing *β* results in a smoother density field but reduces the resolution of atomic-scale features. To evaluate the density field numerically, the simulation domain is discretized into spatial bins of size *d*_*x*_. This bin size determines the trade-off between computational accuracy and efficiency. Smaller values of *d*_*x*_ improve accuracy but increase computational cost, whereas larger values reduce computational effort at the expense of resolution. In this study, a bin size of *d*_*x*_ = 1.0 Å was selected based on previous investigations.^56,57^ Using eqn (3.1) and (3.2) and a prescribed *β*, an OpenMP parallel C++ code is developed to compute the density values. Subsequently, a Python post-processing script is used to extract the mean, minimum, and maximum densities within each *y* − *z* planes for bins along the *x*-direction. To obtain the dimensionless atomic density field, the average density of atomic planes located far from the PB in the bulk phase with the highest density was used as a normalization reference. The average, minimum, and maximum density profiles were then normalized by this bulk value, yielding the relative density profiles *ρ*, *ρ*_min_ and *ρ*_max_, respectively. With this normalization, *ρ* = 1 corresponds to the reference bulk phase, whereas *ρ*^PB^ < *ρ* < 1 in the second bulk phase. The minimum of the average density profile (see red curves in Fig. 3. It should be noted that Fig. 3 contains two PBs) corresponds to the average atomic density within the PB plane *ρ*^PB^, *i.e.*, *ρ*^PB^ = min_*x*_〈*ρ*(**r**)〉_at PB plane_. The parameter *ρ*^PB^ reflects the crystallographic character and nature of the PB. Density fluctuations within each plane are represented by *ρ*_min_ and *ρ*_max_, shown as error bars in Fig. 3. Further details are provided in.^56,57^

**Fig. 3:**
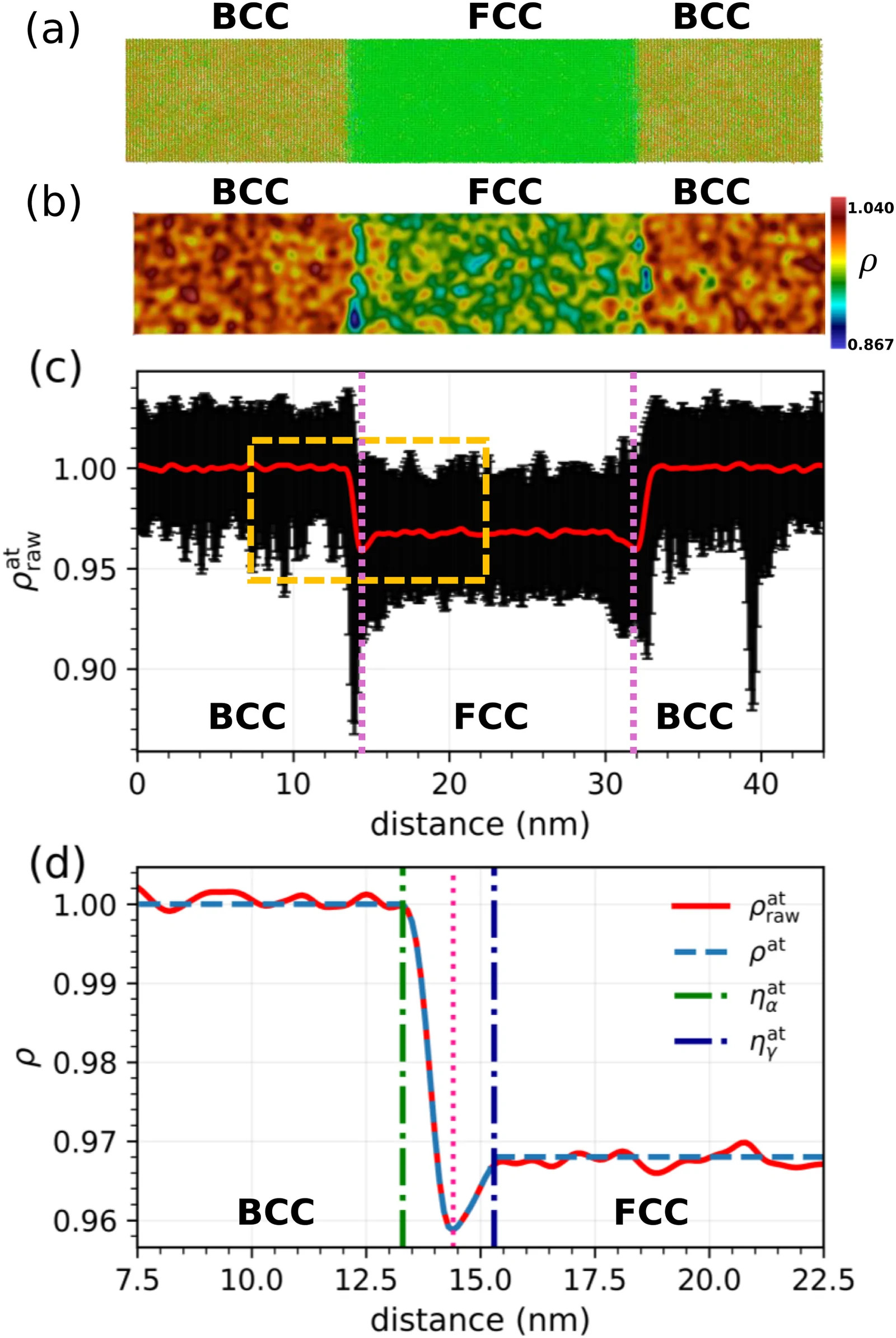
Coarse-graining and density extraction workflow for a BCC–FCC Fe PB. (a) Atomistic configuration based on molecular dynamics simulation at 1000 K obtained from.^58,59^ (b) Construction of the continuous atomic density field by Gaussian coarse-graining with characteristic length *β* = 2.0, using eqn (3.2). (c) Normalized density with respect to the BCC Fe bulk phase. Extracted one-dimensional density profile *ρ*^at^_raw_(*r*_p_) (red line) obtained by averaging over *y* − *z* planes (showing in-plane fluctuations *via ρ*_min_ and *ρ*_max_). (d) Magnified portion (marked by yellow box in (c)) of the processed density profile *ρ*^at^ (dashed blue line) along with the *ρ*^at^_raw_(*r*_p_) (red line), with the retained densities in the perturbed zone and the averaged densities in the bulk phases, and the half-widths *η*^at^_*α*_ and *η*^at^_*γ*_ are extracted from the BCC and FCC sides, respectively.

Fig. 3(a–c) illustrates the concept of coarse-graining using BCC–FCC atomistic Fe PB structure at 1000 K. For a prescribed value of *β*, the atomistic PB structure,^58,59^ whose cross-sectional view is shown in Fig. 3(a), is coarse-grained using eqn (3.1) and (3.2) to obtain the atomic density field across the respective PBs (Fig. 3(b)). By averaging the atomic densities within each plane parallel to the PB plane, a continuous atomic density profile (red curve) is obtained, as shown in Fig. 3(c). The range of density fluctuations within each plane is indicated by the error bars. To illustrate the influence of the coarse-graining length on the PB density profile, four different values of *β* were examined: two close to the cut-off radius (2.0*r*^Fe^_atom_ and 2.4*r*^Fe^_atom_) and two significantly larger (3.0*r*^Fe^_atom_ and 5.0*r*^Fe^_atom_), where *r*^Fe^_atom_ is the atomic radius of Fe. For clarity, the coarse-graining concept is illustrated here for *β* = 2.0*r*^Fe^_atom_ (Fig. 3), while results for other values are provided in Appendix A.4 Fig. 11. In all cases, the density field is normalized with respect to the BCC bulk phase, such that in the BCC bulk *ρ* = *ρ*^at^_BCC_ ≈ 1, in the FCC bulk phase *ρ* = *ρ*^at^_FCC_ < 1, and at the PB plane *ρ*^PB^ < *ρ*^at^_FCC_ < *ρ*^at^_BCC_. However, in Fig. 3(c) and Appendix A.4 Fig. 11, the atomic density inside the BCC and FCC bulk is slightly non-smooth. The non-smoothness originates from thermal fluctuations and local lattice distortions within the bulk BCC and FCC, which lead to occasional misclassification of atoms (*e.g.*, as HCP or unknown) in the original structural analysis of the atomistic PB structure. With increasing *β*, the detailed features of the PB atomic packing become indistinguishable and the boundary region widens, while the *ρ*^PB^ value increases towards 1. This is accompanied by a smoother atomic density profile and reduced in-plane density fluctuations across and within the PB (Fig. 3 and Appendix A.4 Fig. 11). These observations demonstrate that if *β* is too small, the density profile becomes discontinuous. Conversely, large values lead to the loss of important structural information. Consistent with previous coarse-graining studies on GBs, a physically meaningful choice for *β* is at least of the order of the material's lattice constant, which also corresponds closely to the cut-off radius used in atomistic simulations. Accordingly, all subsequent analyses are performed using *β* = 2.0*r*^Fe^_atom_.

For the incorporation of the atomistic information into the developed PB model we consider only the left part of the domain from distance 0 nm to 28.8 nm with the *ρ*^PB^ ≈ 0.959 at ≈14.4 nm. The simulation cell is periodic and contains two PBs (as seen in Fig. 3(a–c), which are crystallographically equivalent by construction. The density descriptors extracted from the two boundaries agree closely, with *ρ*^PB^ = 0.9587 (left PB) and 0.9594 (right PB). The half-widths differ, being *η*_*α*_ ≈ 1.1 nm and *η*_*γ*_ ≈ 0.9 nm at the left PB against ≈1.2 nm and ≈1.6 nm at the right PB, reflecting the different configurations of the two PBs. Only the left boundary is used for the calibration in the present study, as the goal of this section is to establish how atomistic PB configuration can be transformed into an input to the DPF PB model developed. The simulation cell coordinate *x* is measured from the left edge of the atomistic domain, whereas the coordinate *r*_p_ in Section 2 is centered at the PB. The two coordinates are related as *r*_p_ = *x* − *x*_PB_, with *x*_PB_ ≈ 14.4 nm for the PB considered. The PB perturbed zone is identified to range from ≈13.3 nm to ≈15.3 nm and the densities within correspond to the perturbed zone. The rest of the considered range and the associated densities correspond to the bulk phase and the bulk densities. The left side to the perturbed zone is the BCC (*α*) side and their densities are averaged to obtain the *ρ*^at^_*α*_. The right side to the perturbed zone is the FCC (*γ*) side and their densities are averaged to obtain the *ρ*^at^_*γ*_. The obtained density profile with averaged bulk densities and with the retained densities within the perturbed region is shown in Fig. 3(d).

### 3.1. Calibration of the model to the BCC–FCC Fe boundary from atomistic results

In the present study, the BCC Fe phase is referred to as *α* phase and the FCC Fe phase is referred to as *γ* phase. The atomistic profile *ρ*^at^(*r*_p_), given in Fig. 3(d), provides information on the far-field bulk densities on the two sides (*ρ*^at^_*α*_ and *ρ*^at^_*γ*_), the PB plane density *ρ*^PB,at^ (taken as the minimum of the coarse-grained profile), and the geometric half-widths *η*^at^_*α*_ and *η*^at^_*γ*_. For the base case calibration, the *ρ*^PB^ = *ρ*^PB,at^, *ρ*_*α*_ = *ρ*^at^_*α*_ and *ρ*_*γ*_ = *ρ*^at^_*γ*_ are prescribed with the values extracted from the atomistic profile and the continuum parameters are chosen to reproduce the extracted half-widths, while numerically implementing the PB free energy functional at its minimal order (fourth-order gradient regularization and linear traction approximation).

The bulk densities *ρ*^B^_*i*_ are taken from the coarse-grained density profile, whereas the molar volumes *v*_m_^*i*^ are obtained from Thermo-Calc.^62^ Thus, the their values are obtained independently. No quantity other than the two half-widths is targeted for the calibration, and with the values listed in Table 1 the computed half-widths agree with the coarse-grained density profile half-widths. Six coefficients are adjusted against the two half-width targets, so the resulting parameter set is not necessarily unique. For instance, as discussed in Section. 4, the half-widths are governed mainly by *κ*_A,*i*_, whereas the traction contrasts affect *Γ*_ex_ with little influence on the half-widths and are therefore not constrained by the present calibration. All numerical calculations are performed on C++ platform and the figures are produced with Python using.^61^

**Table 1:** Base-case parameters used for the BCC–FCC Fe PB calculations (*α* = BCC, *γ* = FCC). Prescribed quantities are taken directly from the coarse-grained atomistic profile (bulk densities and PB plane density), from the numerical discretization and from the literature. Calibrated quantities are tuned in the base case to reproduce the atomistic-derived half-widths. The molar volumes of the bulk phases *v*_*m*_^*i*^ are obtained from Thermo-Calc Software (version 2023a) TCFE10 Steels/Fe-alloys Database^62^ at 1000 K. The values of *E*^B^_A,*α*_ and *E*^B^_A,*γ*_ are taken from^48^ and,^50^ respectively

Quantity	Value	Unit
**Prescribed (atomistics/discretization/literature)**		
*ρ* ^B^_*α*_	1.0	—
*ρ* ^B^_*γ*_	0.968	—
*ρ* ^PB^	0.959	—
Δ*r*	1.0 × 10^−11^	m
*N*_*α*_, *N*_*γ*_	250, 250	—
*v*_m_^α^	7.314 × 10^−6^	m^3^ mol^−1^
*v*_m_^γ^	7.207 × 10^−6^	m^3^ mol^−1^
*E* ^B^_A,*α*_	−7.72 × 10^4^	J mol^−1^
*E* ^B^_A,*γ*_	−3.97 × 10^4^	J mol^−1^

**Calibrated (base-case fit)**		
*κ*_A,*α*_	1.5 × 10^−13^	J m^2^ mol^−1^
*κ*_A,*γ*_	5.35 × 10^−14^	J m^2^ mol^−1^
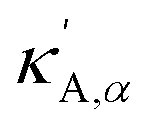	1.0 × 10^−35^	J m^4^ mol^−1^
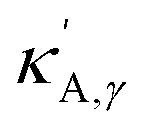	4.0 × 10^−36^	J m^4^ mol^−1^
*ϕ*_*i*_	Hat function^a^	—
*Ψ*_*αα*,0_	1.0 × 10^2^	J mol^−1^
*Ψ*_*αγ*,0_	9.0 × 10^2^	J mol^−1^
*Ψ*_*γγ*,0_	1.0 × 10^2^	J mol^−1^
*Ψ*_*γα*,0_	9.0 × 10^2^	J mol^−1^
*Ψ*_*αα*,1_	1.0 × 10^−5^	J mmol^−1^
*Ψ*_*αγ*,1_	8.0 × 10^−5^	J mmol^−1^
*Ψ*_*γγ*,1_	1.0 × 10^−5^	J mmol^−1^
*Ψ*_*γα*,1_	8.0 × 10^−5^	J mmol^−1^

a Hat functions used for fitting: *ϕ*_*α*_(*r*_p_) = 1 − |*r*_p_|/*η*_*α*_ for −*η*_*α*_ ≤ *r*_p_ ≤ 0, and *ϕ*_*α*_(*r*_p_) = 0 otherwise; *ϕ*_*γ*_(*r*_p_) = 1 − *r*_p_/*η*_*γ*_ for 0 ≤ *r*_p_ ≤ *η*_*γ*_, and *ϕ*_*γ*_(*r*_p_) = 0 otherwise.

Fig. 4(a) compares the coarse-grained atomistic density profile *ρ*_at_(*r*_p_) with the equilibrium density profile *ρ*(*r*_p_) (solution for the derived free energy functional). In addition, the figure shows the smooth initial guess *ρ*_init_(*r*_p_) used for relaxation calculation. The half-widths obtained from the coarse-grained atomistic density profile *η*^at^_*α*_ (≈1.1 nm) and *η*^at^_*γ*_ (≈0.9 nm) and the calculated profiles *η*_*α*_ and *η*_*γ*_ are indicated by vertical lines. With the calibrated parameter set, given in Table 1, the equilibrium profile reproduces the atomistic half-widths on both sides *i.e.*, *η*^at^_*α*_ ≈ *η*_*α*_ and *η*^at^_*γ*_ ≈ *η*_*γ*_, while the remaining difference in the detailed shape close to the PB plane is due to the truncation/approximation of the PB free energy functional in the present implementation. In particular, the atomistic profile exhibits a broader and more gradual variation than the profile obtained by minimizing our free energy functional, suggesting that the higher-order gradient regularization and/or higher-order traction terms omitted here quantitatively affect the full profile shape.

**Fig. 4:**
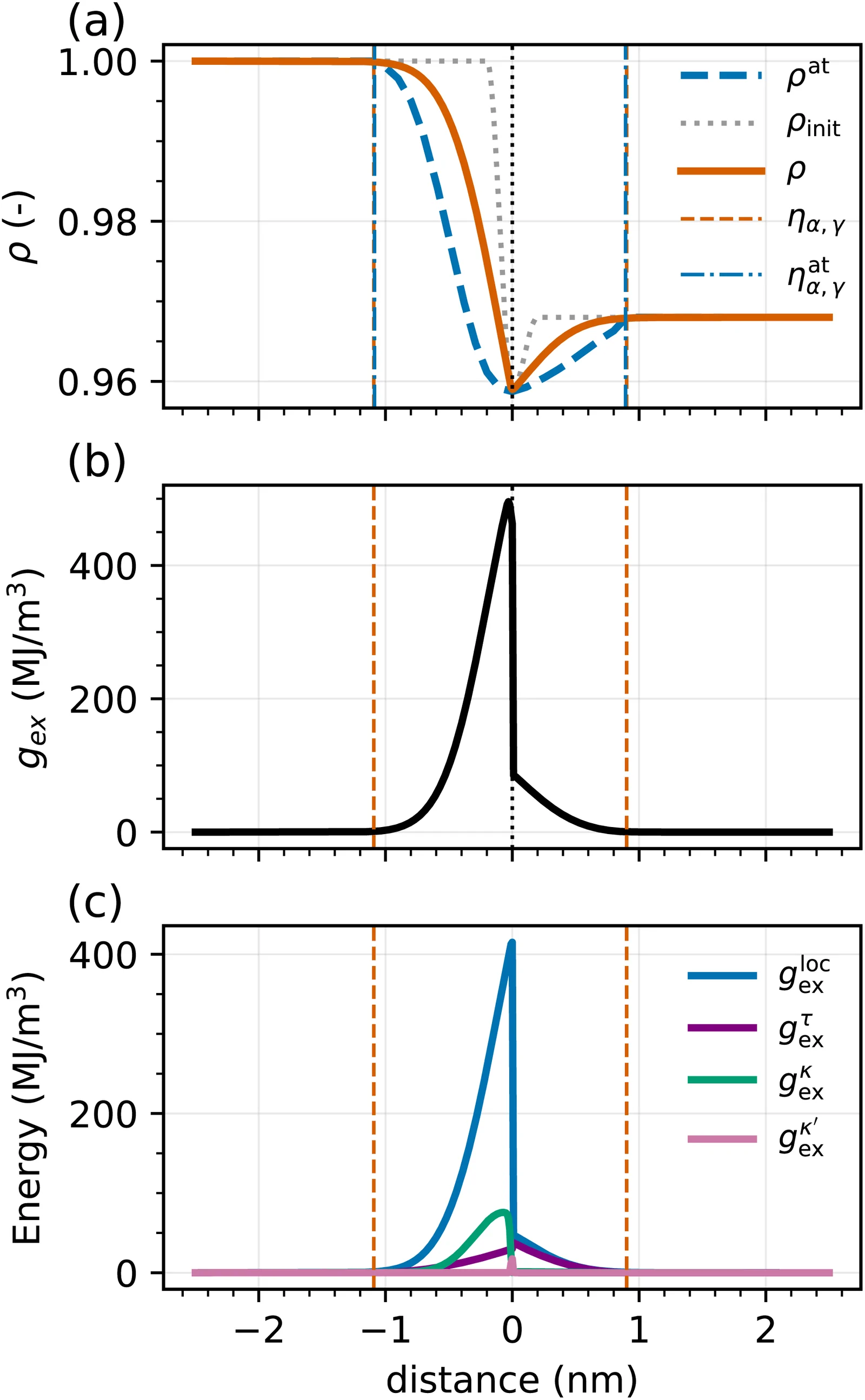
Baseline asymmetric PB solution for BCC–FCC Fe and calibration to atomistics. (a) Coarse-grained atomistic density profile *ρ*^at^(*r*_p_) compared with the continuum initialization *ρ*_init_(*r*_p_) (dashed blue line carried forward from Fig. 3d) and the converged equilibrium profile *ρ*(*r*_p_). Vertical lines indicate the half-widths extracted from atomistics and from the converged continuum solution of PB model. (b) Excess free energy density *g*_ex_(*r*_p_) (J m^−3^) for the calibrated base case, with the equilibrium half-width markers overlaid. (c) Decomposition of the excess density into local, traction, and gradient contributions, *g*^loc^_ex_, *g*_ex_^*τ*^, *g*_ex_^*κ*^, and 
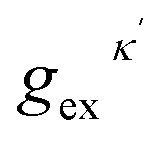
.

The excess free energy density *g*_ex_(*r*_p_) corresponding to the equilibrium density profile *ρ*(*r*_p_) is evaluated on the numerical grid using eqn (2.38) and is shown in Fig. 4(b). The *g*_ex_(*r*_p_) is significant only in the vicinity of the PB plane and decays rapidly into both bulks, where the densities approach their homogeneous reference states. The decay is generally asymmetric across the PB plane, consistent with the side-dependent half-widths extracted from *ρ*(*r*_p_) in Fig. 4(a).

Fig. 4(c) further decomposes the excess density into local, traction, and gradient contributions, *g*^loc^_ex_, *g*_ex_^*τ*^, *g*_ex_^*κ*^, and 
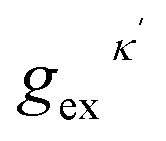
. The *g*^loc^_ex_ is proportional to the deviation of the density from the bulk reference through the local quadratic term. The gradient contribution, *g*_ex_^*κ*^ ∝ *κ*_*i*_|∇*ρ*_*i*_|^2^, suppresses sharp spatial variations of *ρ*(*r*_p_), while the fourth-order term
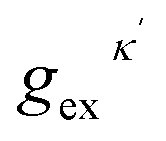
, further regulates the spatial variations. The traction term, *g*_ex_^*τ*^ = *ρτ*_*i*_(*r*_p_), couples the density field to the PB-localized traction field. In this calibration, the local contribution *g*^loc^_ex_ rises sharply in the immediate neighborhood of the PB plane and reaches its largest values on the *α* side. This reflects the strong local energetic cost associated with the PB plane density deficit relative to the bulk reference states, which is most pronounced on the *α*-side of the profile in the present case. The gradient contributions, *g*_ex_^*κ*^ and 
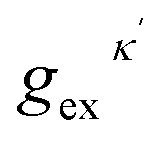
, are comparatively small in magnitude and remain confined near the PB plane. While they act to smooth the density profile, their quantitative contribution to *g*_ex_(*r*_p_) is minor in the present calibration. The traction contribution *g*_ex_^*τ*^ exhibits a broader spatial extent and contributes significantly across both sides of the interface. Thus, the excess free energy density is primarily governed by the local term, with the traction term providing a secondary contribution, while gradient effects remain weak. In computing the energy density at the PB plane, we apply a symmetric PB-node averaging as discussed in detail in Section 2.4. With this convention the maximum of *g*_ex_(*r*_p_) is expected to occur in the immediate neighborhood of the PB plane (rather than necessarily exactly at *r*_p_ = 0). Together, these contributions explain the asymmetric *g*_ex_(*r*_p_) profile in Fig. 4(b).

The computed excess free energy densities are integrated across the domain and the energy per interfacial area (*A*_I_) *Γ*_ex_ is calculated to be ≈0.1931 J m^−2^. The corresponding decomposed contributions *Γ*^loc^_ex_, *Γ*_ex_^*τ*^, *Γ*_ex_^*κ*^ and 
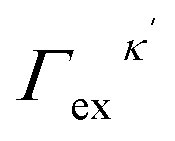
 are calculated to be ≈0.1435 J m^−2^, ≈0.0227 J m^−2^, ≈0.0266 J m^−2^, and ≈0.0003 J m^−2^, respectively. This atomistic-informed base case is used as the reference state for all subsequent parameter studies, and the observed sensitivities are reported relative to this calibrated baseline.

Reproducing the atomistic half-widths is a calibration strategy, since the coarse-grained density profile supplies both the density descriptors and the fitting targets (half-widths), and is not an independent validation of the developed model. An interfacial energy for the considered PB is not reported in.^58,59^ However, Nagano and Enomoto^60^ report *α* − *γ* interfacial energies in pure Fe for the Nishiyama–Wasserman orientation relationship, varying the interface orientation by rotation from the close-packed plane. They report the interfacial energy to be small, with a minimum of 0.32 J m^−2^, for rotations of less than about 15°, which includes the considered PB. The value computed in the present study is of the same order but lower, since different interatomic potential were used in ^58,59^ and ^60^

## 4. Systematic evaluation of the model's performance

In order to explore the performance of our model, we systematically investigate the case of BCC–FCC Fe PB calibrated above. We focus on the effect of various model parameters on two key quantities. First, the PB half-widths *η*_*α*_ and *η*_*γ*_ which quantify how far the interfacial effects extend into the *α* and *γ* bulk phases, respectively. Second, the excess free energy *Γ*_ex_ which represents the thermodynamic cost of forming the PB relative to the corresponding bulk reference states.

### Effect of bulk gradient-penalty coefficients *κ*_A,*i*_ and 
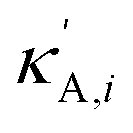


4.1.

The gradient coefficients regulate the energetic cost of spatial density variations and therefore play an important role in the spatial extent and energetics of the PB. In the present formulation, two distinct regularization mechanisms are used for each phase. The second-order gradient penalty, governed by *κ*_A,*α*_ and *κ*_A,*γ*_, and the fourth-order gradient penalty, governed by 
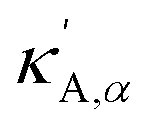
 and 
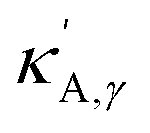
. Although both terms suppress sharp density variations, they act in quantitatively different ways. The second-order terms primarily influence the spatial extent of the density transition, as measured by the PB half-widths, whereas the fourth-order terms mainly affect the curvature and smoothness of the density profile.

In this investigation, one gradient coefficient is varied while all the other model parameters are kept fixed at the calibrated base case values listed in Table 1. The black-edged markers in Fig. 5 and 6 indicate the calibrated base case. Fig. 5 shows the effect of varying *κ*_A,*α*_ and *κ*_A,*γ*_ on half-widths and excess free energy, while holding the other parameters fixed at the calibrated base case. With increasing *κ*_A,*α*_, the *η*_*α*_ increases significantly, whereas the *η*_*γ*_ exhibits only a slight increase as shown in Fig. 5(a). On the other hand, with increasing *κ*_A,*γ*_, the *η*_*γ*_ increases significantly, but the effect on *η*_*α*_ is small as shown in Fig. 5(b). Although, the *α*- and *γ*-phase domains are solved separately, the effect of *κ*_A,*α*_ propagates to the *γ*-side *via* the one-sided slopes used in the PB traction term. However, within the considered parameter range, the effect of *κ*_A,*α*_ on *η*_*γ*_ is stronger than the effect of *κ*_A,*γ*_ on *η*_*α*_. The cross-phase influence is asymmetric. Variations in *κ*_A,*α*_ produce a noticeable change in *η*_*γ*_, whereas variations in *κ*_A,*γ*_ produce nearly no response in *η*_*α*_. This asymmetry indicates that modifications introduced on the *α*-side are transmitted more effectively through the PB traction coupling than changes introduced on the *γ*-side.

**Fig. 5:**
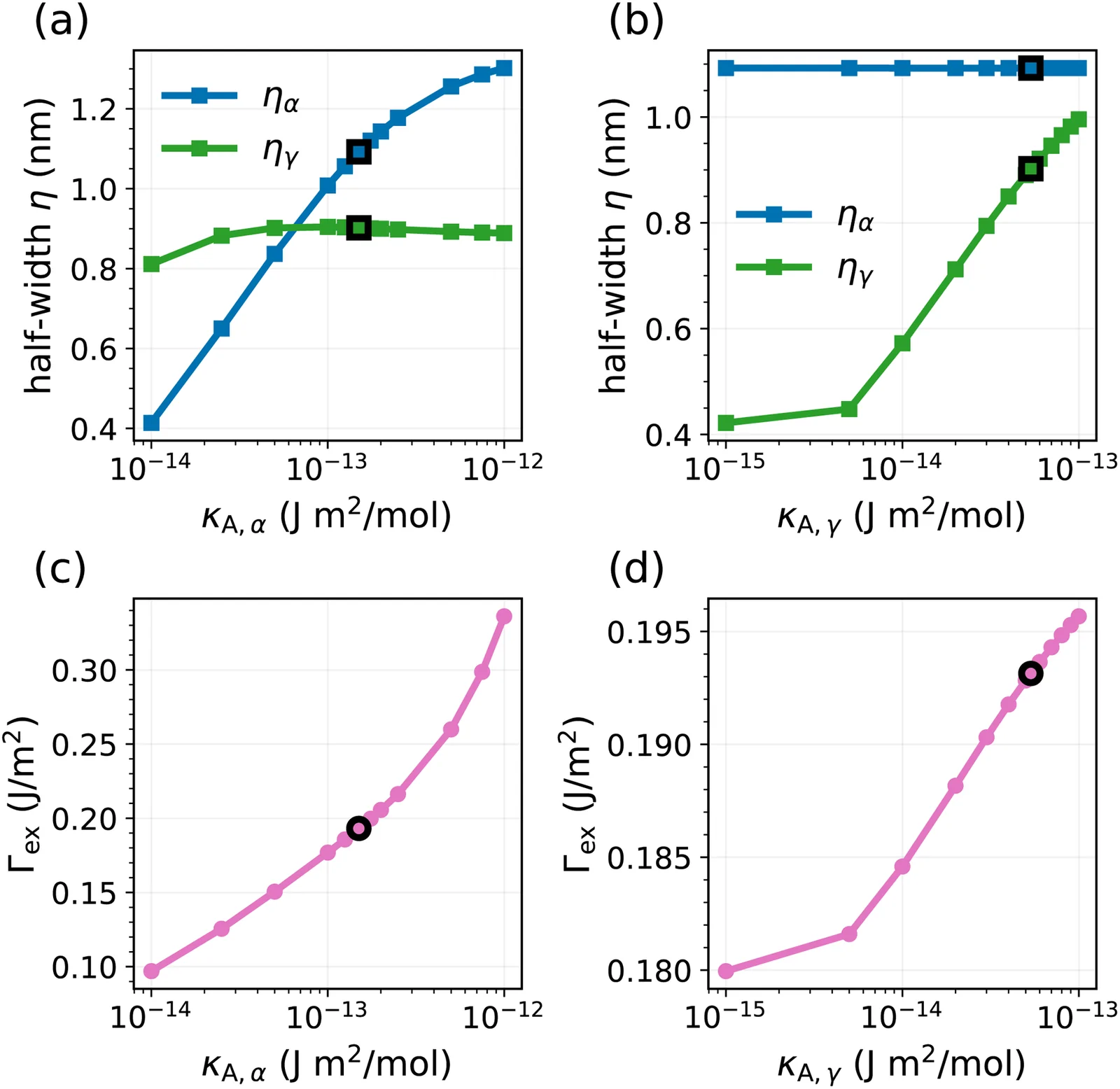
Effect of second-order gradient penalty coefficients on PB half-widths and excess free energy. (a) Variation of the equilibrium PB half-widths *η*_*α*_ and *η*_*γ*_ with *κ*_A,*α*_ keeping all other parameters fixed to the calibrated base case. (b) Variation of *η*_*α*_ and *η*_*γ*_ with *κ*_A,*γ*_. (c) Variation of the excess free energy *Γ*_ex_ with *κ*_A,*α*_. (d) Variation of *Γ*_ex_ with *κ*_A,*γ*_. The calibrated base case is indicated by markers with black edges.

**Fig. 6:**
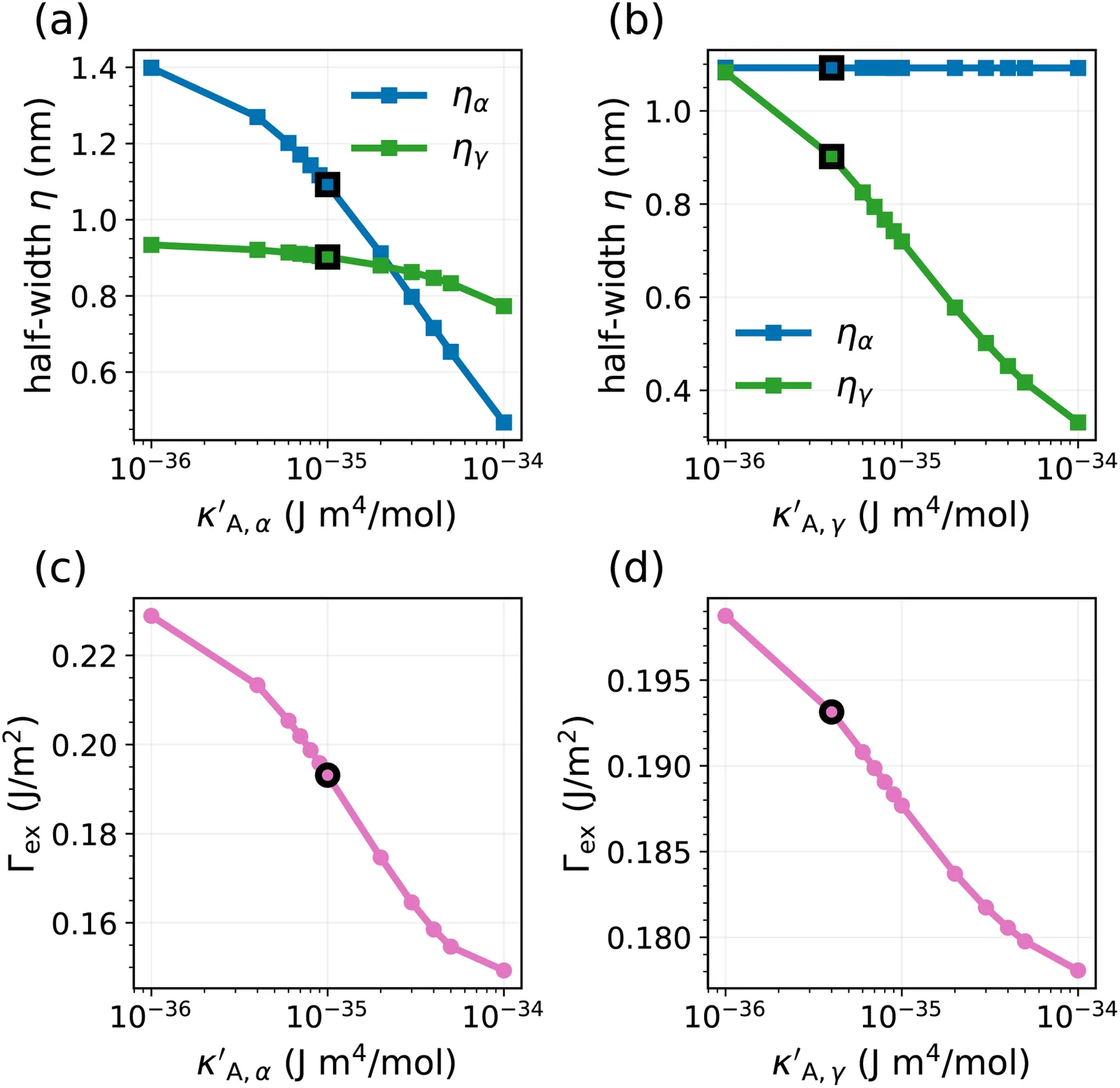
Effect of fourth-order gradient penalty coefficients on PB half-widths and excess free energy. (a) Variation of the equilibrium PB half-widths *η*_*α*_ and *η*_*γ*_ with 
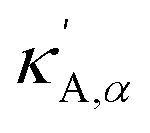
 keeping all other parameters fixed to the calibrated base case. (b) Variation of *η*_*α*_ and *η*_*γ*_ with 
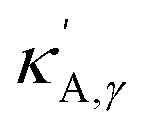
. (c) Variation of the excess free energy *Γ*_ex_ with 
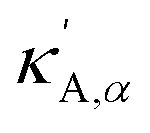
. (d) Variation of *Γ*_ex_ with 
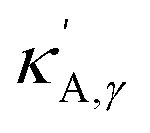
. The calibrated base case is indicated by markers with black edges.

The effect of *κ*_A,*α*_ and *κ*_A,*γ*_ on the excess free energy is shown in Fig. 5(c and d). As expected, the excess free energy *Γ*_ex_ increases with the increasing both *κ*_A,*α*_ and *κ*_A,*γ*_. These second-order gradient penalty coefficients disfavor sharp density changes and promote broader interfacial zones. However, the effect of changes in *κ*_A,*α*_ on *Γ*_ex_ is much larger than the effect of variations in *κ*_A,*γ*_. The strong effect of *κ*_A,*α*_ on *Γ*_ex_ is consistent with the significant changes observed in *η*_*α*_ over the considered parameter range.

Fig. 6 shows the effect of varying the 
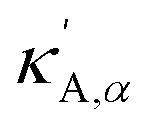
 and 
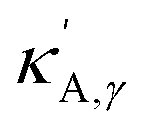
 on half-widths *η*_*α*_ and *η*_*γ*_, in panels (a) and (b) respectively. In contrast to the second-order penalty coefficients, increasing 
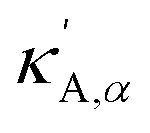
 leads to a reduction in the corresponding *η*_*α*_, while *η*_*γ*_ exhibits slight decrease. On the other hand, with increasing 
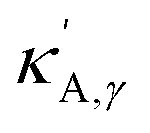
, *η*_*γ*_ decreases and *η*_*α*_ remains nearly constant. This behavior demonstrates the different role of the fourth-order contribution. These terms 
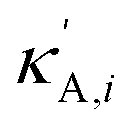
 rather than promoting broader density transitions, suppresses high-curvature variations and is accompanied by a reduction in the computed PB half-widths within the considered parameter range. The cross-phase effect of 
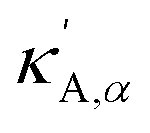
 on *η*_*γ*_ is significant as compared to the effect of 
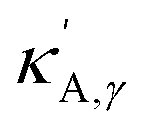
 on *η*_*α*_. This is similar to the cross-phase effects of second-order penalty coefficients *κ*_A,*i*_. Similar to the second-order coefficients, the cross-phase effect of 
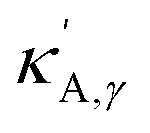
 on the *α*-side remains weak throughout the investigated parameter range. The *Γ*_ex_ decreases with increasing 
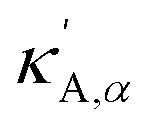
 and 
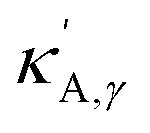
 as shown in Fig. 6(c and d). This decrease in excess free energy indicates that increasing 
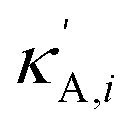
 has a stabilizing effect on the PB structure over the considered parameter range. Thus, the fourth-order gradient term acts as a stabilizer that distributes the excess free energy away from sharp curvature without enforcing large interfacial widths.

Overall, the results show that the second-order and fourth-order gradient coefficients affect the PB in quantitatively different ways. Increasing *κ*_A,*i*_ tends to increase the half-widths and the excess free energy, whereas increasing 
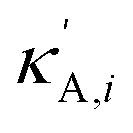
 reduces both the PB half-width and the excess free energy over the considered parameter range.

### 4.2. Effect of local bulk stiffness *E*^B^_A,*α*_ and *E*^B^_A,*γ*_

The quadratic local term controls the energetic penalty for deviations of the density from its bulk value in each phase. As the local term favors the corresponding bulk density, the balance between this term and the gradient energy contributions determines the density profile and the interface width. Since *E*^B^_A,*i*_ < 0, this penalty becomes stronger as *E*^B^_A,*i*_ becomes more negative and weaker as *E*^B^_A,*i*_ becomes less negative. For this study, we vary *E*^B^_A,*α*_ and *E*^B^_A,*γ*_ independently around the calibrated base case while keeping all other parameters fixed.

As *E*^B^_A,*α*_ becomes less negative, both *η*_*α*_ and *η*_*γ*_ decrease, as shown in Fig. 7(a). The reduction in *η*_*γ*_ is smaller than that in *η*_*α*_, but it remains noticeable, indicating a cross-phase effect through the PB traction interaction. A complementary trend is observed on the *γ* side. As *E*^B^_A,*γ*_ becomes less negative, the *γ*-side half-width *η*_*γ*_ decreases appreciably, while *η*_*α*_ remains nearly constant, as shown in Fig. 7(b). The response to variations in *E*^B^_A,*γ*_ is more localized to the *γ*-side, whereas variations in *E*^B^_A,*α*_ influence both sides of the PB.

**Fig. 7:**
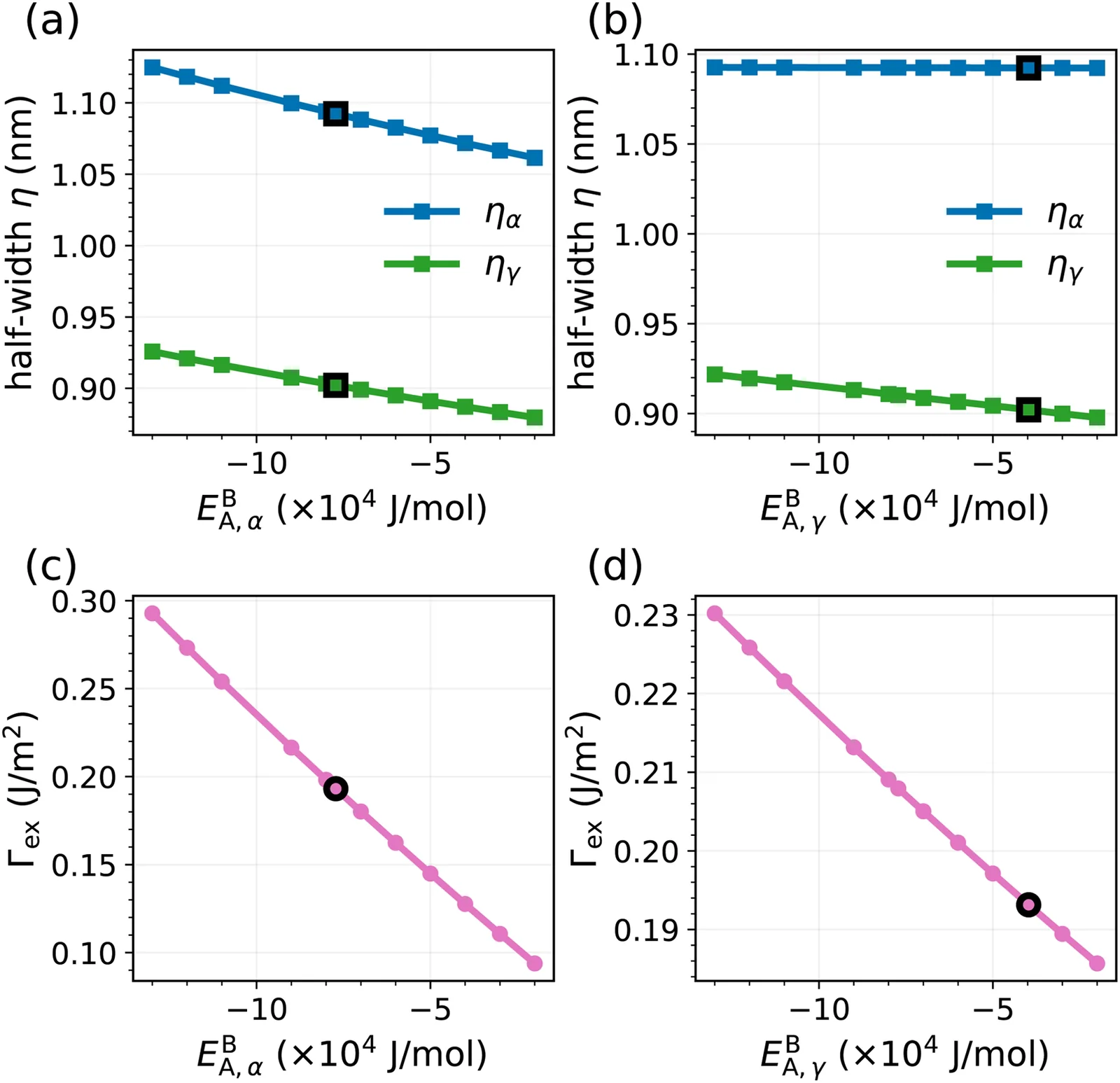
Effect of phase-specific local bulk stiffness on PB half-widths and excess free energy. (a) Variation of the equilibrium PB half-widths *η*_*α*_ and *η*_*γ*_ with *E*^B^_A,*α*_, keeping all other parameters fixed to the calibrated base case. (b) Variation of *η*_*α*_ and *η*_*γ*_ with *E*^B^_A,*γ*_. (c) Corresponding variation of the excess free energy *Γ*_ex_ with *E*^B^_A,*α*_. (d) Corresponding variation of *Γ*_ex_ with *E*^B^_A,*γ*_. The calibrated base case is indicated by markers with black edges.

The corresponding variation of the excess free energy is shown in Fig. 7(c and d). It is observed that as *E*_A,*i*_ becomes less negative, *Γ*_ex_ decreases over the considered parameter range. Since *g*^loc,*i*^_ex_ is proportional to *E*_A,*i*_, weakening the local penalty (less negative *E*_A,*i*_) reduces the energetic cost, and hence reduces *Γ*_ex_. Over the considered parameter range, the reduction in *Γ*_ex_ is approximately linear with *E*_A,*i*_. The sensitivity of *Γ*_ex_ to *E*^B^_A,*α*_ is considerably larger than the sensitivity to *E*^B^_A,*γ*_, as shown in Fig. 7(c) and (d). Moreover, the same-phase half-width decreases more strongly on the *γ* side (on varying *E*^B^_A,*γ*_) than on the *α* side (on varying *E*^B^_A,*α*_), whereas the drop in *Γ*_ex_ is more pronounced for the *E*^B^_A,*α*_ sweep. These trends are consistent with the strong influence of *E*_A,*i*_ on the excess free energy and of *κ*_A,*i*_ on the half-width *η*_*i*_.

### 4.3. Effect of bulk-density mismatch *via ρ*^B^_*γ*_

To study how sensitive the PB density profile and the associated energetics are to a bulk-density mismatch (across the PB), we vary the *γ*-phase bulk density *ρ*^B^_*γ*_ while keeping all other parameters fixed to the calibrated base case, including the PB plane density *ρ*^PB^ and the *α*-side bulk density. This also allows us to assess the applicability of the developed PB thermodynamic model to a PB between any two phases and not limited to *α* (BCC) and *γ* (FCC) phases.

Fig. 8(a) shows the equilibrium density profiles for selected *ρ*^B^_*γ*_ values. Since *ρ*^PB^ is held fixed, changing *ρ*^B^_*γ*_ primarily affects the recovery of the density profile on the *γ*-side. In addition, the density recovery on the *γ*-side becomes steeper as the mismatch between *ρ*^B^_*γ*_ and *ρ*^PB^ increases, leading to larger PB-adjacent density gradients. For *ρ*^B^_*γ*_ > *ρ*^PB^, the density increases away from the PB plane to reach the higher bulk value. In contrast, the *α*-side density remains nearly unchanged over the *ρ*^B^_*γ*_ variation, since *ρ*^B^_*α*_ is unchanged.

**Fig. 8:**
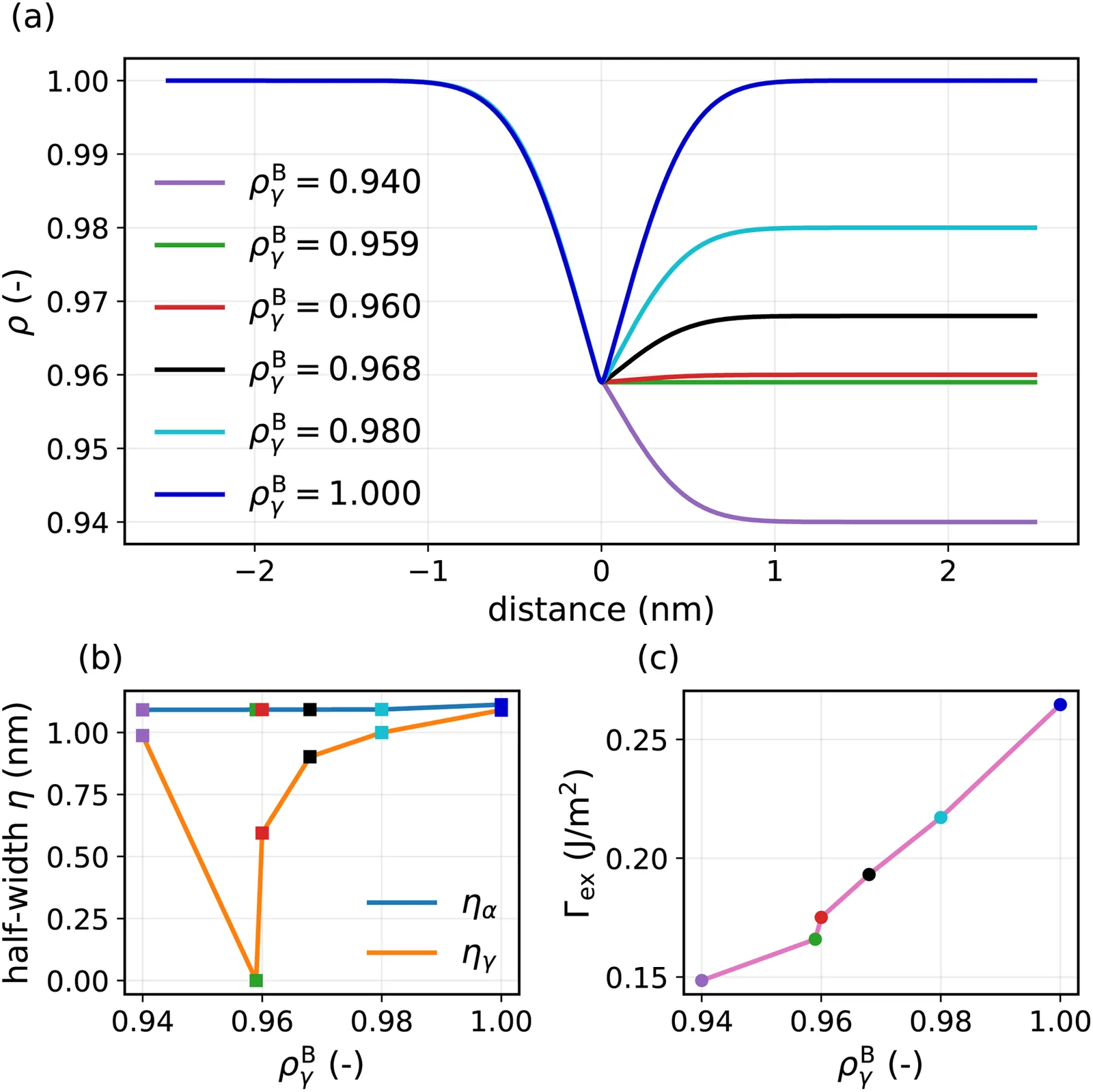
Sensitivity of PB structure and energetics to the *γ*-phase bulk density *ρ*^B^_*γ*_. (a) Equilibrium density profiles for selected *ρ*^B^_*γ*_ values with all other parameters fixed to the calibrated base case (including pinned *ρ*^PB^). (b) Corresponding half-widths *η*_*α*_ and *η*_*γ*_*versus ρ*^B^_*γ*_. (c) Excess-energy partition *Γ*_ex_*versus ρ*^B^_*γ*_. The calibrated base case is highlighted by black markers.

The corresponding PB half-widths and excess energies are plotted in Fig. 8(b) and (c), respectively, with colors matching those of the corresponding density profiles in Fig. 8(a). Fig. 8(b) shows that *η*_*α*_ remains nearly constant over the investigated range of *ρ*^B^_*γ*_. In contrast, *η*_*γ*_ is sensitive to the bulk-density mismatch and increases as *ρ*^B^_*γ*_ moves away from *ρ*^PB^. For values of *ρ*^B^_*γ*_ very close to *ρ*^PB^, the *η*_*γ*_ is very small and reaches a value of zero when *ρ*^B^_*γ*_ = *ρ*^PB^. The effect on energy is shown in Fig. 8(c). The excess free energy *Γ*_ex_ increases with increase in *ρ*^B^_*γ*_. For values of *ρ*^B^_*γ*_ close to *ρ*^PB^, the computed *η*_*γ*_ becomes very small and approaches zero when *ρ*^B^_*γ*_ = *ρ*^PB^. In this case, no recovery region is required on the *γ*-side as the PB density matches the bulk density. Consequently, the excess free energy contribution from the *γ*-side is small, resulting in a lower value of *Γ*_ex_. As *ρ*^B^_*γ*_ moves away from *ρ*^PB^, the recovery region on the *γ*-side becomes more pronounced due to the increasing density deviation from the bulk state and the gradient contributions. Unlike the variations in *E*^B^_A,*i*_, the effect of *ρ*^B^_*γ*_ remains largely confined to the *γ* side, with only negligible influence on the *α*-side profile.

For *ρ*^B^_*γ*_ = 0.94, where *ρ*^B^_*γ*_ < *ρ*^PB^, the density decreases away from the PB plane towards the *γ*-bulk value. Thus, a finite recovery region is required on the *γ*-side and *η*_*γ*_ increases after passing through the minimum at *ρ*^B^_*γ*_ = *ρ*^PB^. However, the *g*^loc,*γ*^_ex_ = *E*_*γ*_*ρ*_*γ*_(*ρ*_*γ*_ − *ρ*^B^_*γ*_) with *E*_*γ*_ < 0 and *ρ*^B^_*γ*_ < *ρ*^PB^ on the *γ* side becomes negative. This negative *g*^loc,*γ*^_ex_ reduces *Γ*_ex_, contributing to the lower values of *Γ*_e*x*_ than observed near *ρ*^B^_*γ*_ = *ρ*^PB^.

### 4.4. Effect of interfacial structural state

Physically, *ρ*^PB^ represents the local packing state at the PB plane and therefore serves as a descriptor of the interfacial structural state. Varying *ρ*^PB^ provides a direct way to study how the PB density profile and the associated thermodynamics evolve as the interface core is made progressively more open (small *ρ*^PB^) or more bulk-like (large *ρ*^PB^), while keeping the bulk densities and other parameters fixed.

Fig. 9(a) shows equilibrium density profiles for selected *ρ*^PB^ values. Decreasing *ρ*^PB^ deepens the density deficit at the PB plane, resulting in steeper near-PB density gradients and wider recovery regions on both sides of the interface. As *ρ*^PB^ is increased towards the bulk densities, the density profiles approach the bulk values more rapidly and the contrast between the PB plane and the adjacent bulk regions diminishes.

**Fig. 9:**
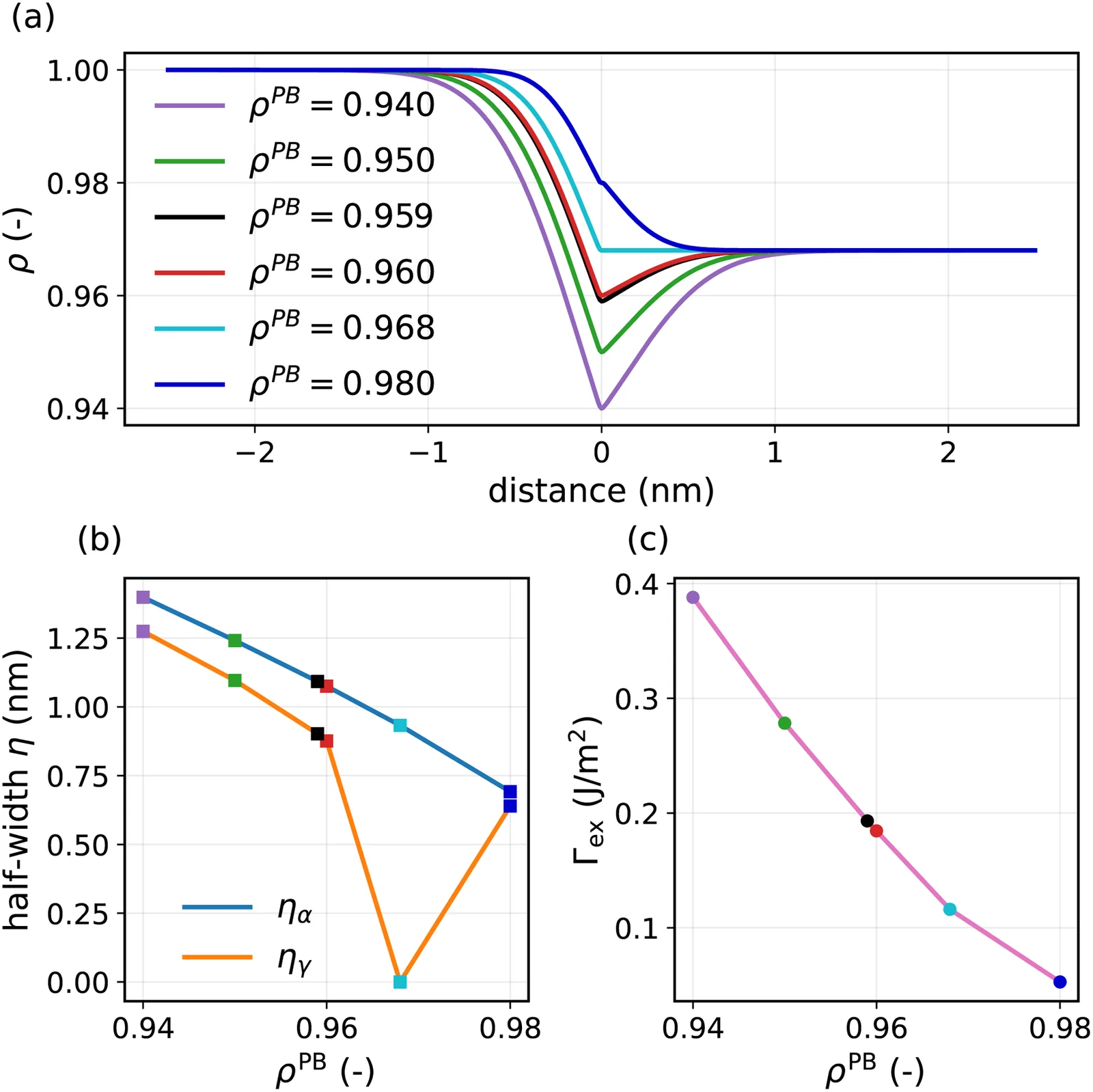
Influence of prescribed PB plane density *ρ*^PB^ on PB structure and excess free energy. (a) Equilibrium density profiles for selected values of *ρ*^PB^, showing progressive weakening of the interfacial perturbation as *ρ*^PB^ approaches the bulk densities. (b) Corresponding equilibrium half-widths *η*_*α*_ and *η*_*γ*_ as functions of *ρ*^PB^. (c) Excess-energy partition *Γ*_ex_*versus ρ*^PB^.The calibrated base case is highlighted by black markers.

The corresponding PB half-widths and excess energies are plotted in Fig. 9(b) and (c), respectively, with colors matching those of the corresponding density profiles in Fig. 9(a). Over most of the considered range, both *η*_*α*_ and *η*_*γ*_ decrease as *ρ*^PB^ increases, as shown in Fig. 9(b). As the PB plane density becomes more bulk-like, the density profile requires a shorter recovery length to reach the corresponding far-field bulk state. For values of *ρ*^PB^ close to *ρ*^B^_*γ*_, the computed *η*_*γ*_ decreases significantly and approaches zero when *ρ*^PB^ = *ρ*^B^_*γ*_. In this limit, no recovery region is required on the *γ* side because the PB density already matches the *γ*-phase bulk density. For *ρ*^PB^ > *ρ*^B^_*γ*_, the density mismatch reappears and *η*_*γ*_ increases again. The excess free energy *Γ*_ex_ decreases with increasing *ρ*^PB^, indicating that the density mismatch between the PB plane and the surrounding bulk states plays an important role in the overall PB excess free energy. For *ρ*^PB^ = 0.98, where *ρ*^PB^ > *ρ*^B^_*γ*_, the density decreases from the PB plane towards the *γ*-bulk value. In this regime, a finite recovery region is required and *η*_*γ*_ increases. However, the *Γ*_ex_ continues to decrease because the *g*^loc,*γ*^_ex_ on the *γ* side changes sign. Since *g*^loc,*γ*^_ex_ = *E*_*γ*_*ρ*_*γ*_(*ρ*_*γ*_ − *ρ*^B^_*γ*_) with *E*_*γ*_ < 0, the local contribution becomes negative when *ρ*_*γ*_ > *ρ*^B^_*γ*_. This negative *g*^loc,*γ*^_ex_ contribution reduces the *Γ*_ex_, despite the increase in the *γ*-side half-width.

In general, the *ρ*^PB^ variation study highlights *ρ*^PB^ as an effective control parameter for the interfacial structural state. Lower *ρ*^PB^ produces a deeper, high energy interfacial region with stronger gradients and wider half-widths, while higher *ρ*^PB^ leads to weaker gradients, reduced half-widths, and lower interfacial excess free energy.

### 4.5. Traction sensitivity for different cap-moment contrasts

The traction term represents the energetic interaction between the *α*- and *γ*-sides across the PB. It provides the coupling between the two phases through the PB density, the one-sided PB slopes and the PB half-widths. In the most general form, the first-order traction amplitudes depend on four cap moments on each side (cross and self channels for *ℓ* = 0, 1), which would lead to a large parameter space. To reduce the parameters, we impose reciprocity for cross interactions and a common-self assumption, *Ψ*_*αγ*,*ℓ*_ = *Ψ*_*γα*,*ℓ*_ and *Ψ*_*αα*,*ℓ*_ = *Ψ*_*γγ*,*ℓ*_ for *ℓ* = 0, 1, and characterize traction by two contrasts
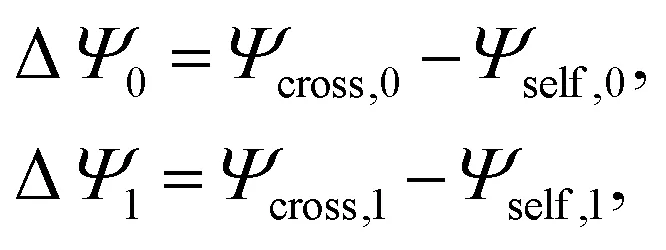
where *Ψ*_cross,*ℓ*_ denotes the common cross-channel cap moment and *Ψ*_self,*ℓ*_ the common self-channel cap moment. In this way, Δ*Ψ*_0_ takes into account the part of the traction amplitude proportional to the PB plane density *ρ*^PB^, whereas Δ*Ψ*_1_ takes into account the slope-weighted contribution associated with the first cap moment.

Fig. 10 summarizes the effect of both traction contrasts on half-widths and excess energies. First, Δ*Ψ*_0_ is varied while Δ*Ψ*_1_ and all other parameters are fixed. The equilibrium half-widths show only a very weak dependence on Δ*Ψ*_0_. Both *η*_*α*_ and *η*_*γ*_ remain nearly unchanged over the investigated range, as shown in Fig. 10(a). In contrast, the excess free energy increases noticeably with increasing Δ*Ψ*_0_, as shown in Fig. 10(c).

**Fig. 10:**
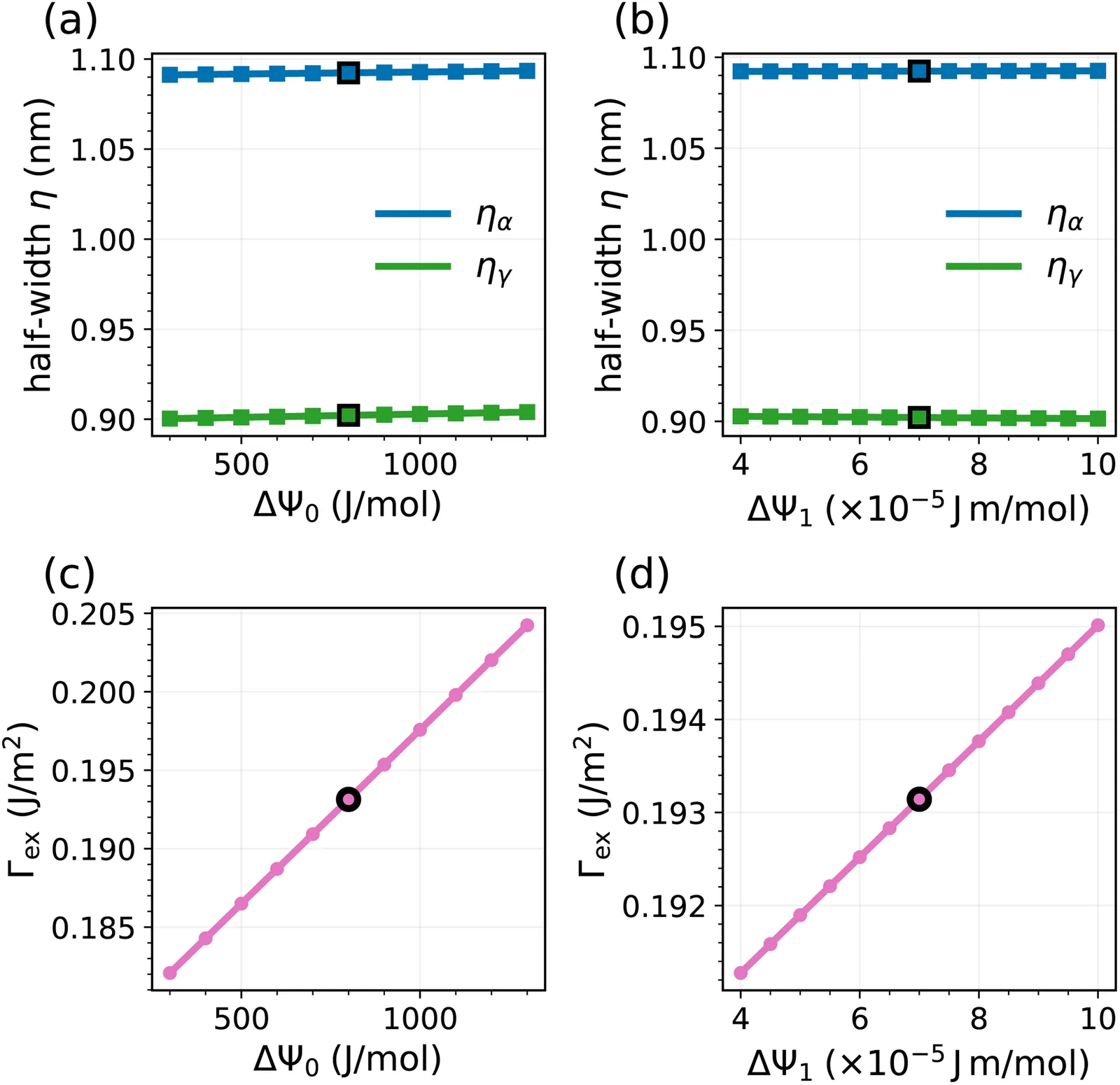
Traction sensitivity to the cap-moment contrasts Δ*Ψ*_0_ and Δ*Ψ*_1_. Reciprocity and common-self assumptions are imposed, *Ψ*_*αγ*,*ℓ*_ = *Ψ*_*γα*,*ℓ*_ and *Ψ*_*αα*,*ℓ*_ = *Ψ*_*γγ*,*ℓ*_ for *ℓ* = 0, 1, so that the traction is characterized by the cross-self contrasts Δ*Ψ*_0_ = *Ψ*_cross,0_ − *Ψ*_self,0_ and Δ*Ψ*_1_ = *Ψ*_cross,1_ − *Ψ*_self,1_. The base case (black markers) corresponds to Δ*Ψ*_0_ = 800 J mol^−1^ and Δ*Ψ*_1_ = 7 × 10^−5^ J m mol^−1^. (a and c) Variation with Δ*Ψ*_0_ at fixed Δ*Ψ*_1_: (a) equilibrium half-widths *η*_*α*_ and *η*_*γ*_ and (c) excess free energy *Γ*_ex_. (b and d) Variation with Δ*Ψ*_1_ at fixed Δ*Ψ*_0_: (b) half-widths *η*_*α*_ and *η*_*γ*_ and (d) excess free energy *Γ*_ex_.

Secondly, Δ*Ψ*_1_ is varied while keeping Δ*Ψ*_0_ and all other parameters fixed. The influence of Δ*Ψ*_1_ on the half-widths is also weak, as shown in Fig. 10(b), with no significant change in either *η*_*α*_ or *η*_*γ*_ over the investigated range. The excess free energy increases with increasing Δ*Ψ*_1_, approximately following a linear trend, as shown in Fig. 10(d). However, the magnitude of this increase is considerably smaller than that observed for variations in Δ*Ψ*_0_.

Overall, both traction contrasts have only a weak influence on the equilibrium PB half-widths. Their primary effect is reflected in the excess free energy, with Δ*Ψ*_0_ producing a considerably larger energetic response than Δ*Ψ*_1_ over the considered parameter range.

## 5. Discussion

Several perspectives of the present study are worth further discussing. A first notable outcome of the present density-based PB formulation is the emergence of asymmetric half-widths on the two sides of the PB plane, with *η*_*γ*_ < *η*_*α*_, although the model imposes only density continuity at the PB plane and fixed far-field bulk conditions. This asymmetry is not prescribed. It arises because the two phases generally possess different local stiffness, different gradient regularization, and different bulk densities, so that each half-width is governed mainly by its own-phase coefficients *E*_A,*i*_, *κ*_A,*i*_, 
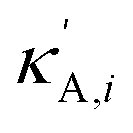
 and *ρ*^B^_*i*_, as observed from Fig. (5–8). The traction acts as a shared coupling between the two sides. The resulting asymmetry is consistent with the coarse-grained atomistic profile to which the present PB model is calibrated, discussed in Section 3.1, and is expected to be generic for PBs, since the two bulk phases meeting at a PB rarely share identical energetic and structural states.

Beyond the asymmetry, the formulation offers a key practical advantage. The PB is captured in a continuum framework through only a small set of density descriptors which are physically interpretable. The density (structural) descriptors, namely the far-field bulk densities *ρ*^B^_*α*_ and *ρ*^B^_*γ*_ and the PB plane density *ρ*^PB^, are extracted directly from atomistic simulations using the coarse-graining approach discussed in Section 3. Here, *ρ*^PB^ quantifies how open or compact the interface core is, while *ρ*^B^_*α*_ and *ρ*^B^_*γ*_ fix the far-field bulk phase densities that the continuum profile must reach. Moreover, the spatial extent over which the profile recovers to the bulk on each side is the PB half-widths *η*_*α*_ and *η*_*γ*_, which the continuum model reproduces and which therefore complete the geometric description of the interface.

The remaining parameters *E*_A,*i*_, *κ*_A,*i*_ and 
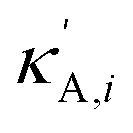
 then control how strongly the deviations from these reference states are corrected. The contrast Δ*Ψ*_0_ multiplies *ρ*^PB^ and acts as a density-weighted offset, while Δ*Ψ*_1_ multiplies the one-sided PB slopes and controls the slope-coupled response. Overall, this gives a clear and transparent way to apply the developed PB model to other PBs, and not only to the present *α*(BCC)–*γ*(FCC) Fe case. For a different PB, the density descriptors and the PB half-widths are taken directly from the coarse-grained atomistic profile, the local stiffness *E*_A,*i*_ is taken from the available literature, and the remaining parameters *κ*_A,*i*_, 
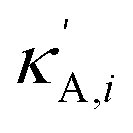
, Δ*Ψ*_0_ and Δ*Ψ*_1_ are re-calibrated to match the atomistic profile and its energetics. Since the PB plane density and the half-widths are extracted from a specific PB configuration, the coefficients obtained here apply to that specific PB and are not representative of BCC–FCC PBs in general.

The free energy densities, given in eqn (2.29), (2.30), (2.31) and (2.32), and density potential, given in eqn (2.36), are frame invariant and not direction specific. The reduction of the functional to a single coordinate *r*_p_ is due to the assumption of a planar PB in the present work. For a curved PB, **p̂** is the local unit normal and *r*_p_ is the distance along it. Then the Laplacian term takes the form,5.1
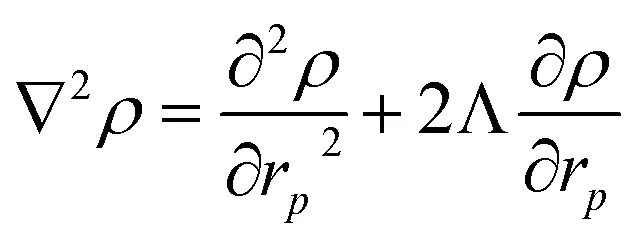
and5.2
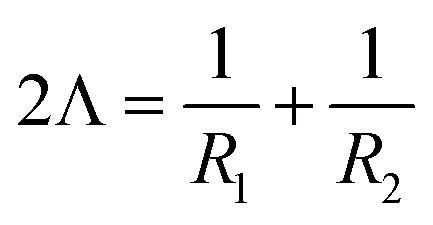
where *Λ* is the local mean curvature and *R*_1_, *R*_2_ are the radii of curvature. Thus the curvature enters through the *κ*_A,*i*_∇^2^*ρ*_*i*_ term already present in the described planar PB case. However, the traction term and cap moments construction discussed in Appendix-A.1 and Appendix-A.2, respectively, are derived for a planar PB. Thus, the present formulation requires *R* ≫ *η*_*i*_ to be applied to a curved PB. It is also to note that addressing nucleation and associated phenomena requires further expansion of the current model to obtain the driving forces explicitly along with the interfacial excess free energy description. Such driving force is accessible from the CALPHAD assessments integrated into the DPF framework,^48–50^ but these aspects are outside the scope of the present work.

Furthermore, it is to note that the present model development uses a minimal, yet consistent implementation of the derived PB functional, with gradient regularization up to fourth order and a first-order (linear) traction approximation. Even with these approximations, the model captures side-dependent half-widths and the associated excess free energy. At the same time, the comparison with coarse-grained atomistics (Fig. 4) shows the limits of this minimal description. The model does not reproduce the detailed density profile shape near the PB plane quantitatively, particularly where the atomistic profile shows broader shoulders than those of the model-calculated profile. Thus, the following two extensions are identified as scope for future work. Gradient regularization terms beyond fourth order would give additional control over the curvature and the shoulder formation, and extending the traction term beyond the first-order approximation would introduce further interfacial shape descriptors in addition to the one-sided slopes. Once such higher-order terms are included, the model should be able to capture the PB density profile more quantitatively and in turn improve the accuracy of the computed PB excess energies.

## 6. Summary

In this work, we build a density-based thermodynamic description of PBs using a continuum atomic density field. Each side of the boundary is referenced to its own homogeneous bulk phase, and the PB is treated as a localized departure from these bulk references. This leads to a Gibbs free energy functional with three contributions: a bulk referenced local term that penalizes density deficits relative to bulk, a phase-specific gradient term that penalizes sharp spatial variations and curvature of the density profile through second- and fourth-order regularization, and a PB-specific traction term that represents cross-boundary interactions. For numerical calculations we implement a minimal version of the functional using gradient regularization up to fourth order and a first-order (linear) traction approximation.

To connect the model to atomistics, we coarse-grain an atomistic BCC–FCC Fe PB at 1000 K and extract three key density descriptors: the two bulk densities and the PB plane density. A base-case parameter set is then calibrated so that the continuum equilibrium profile reproduces the atomistic-derived half-widths and yields a consistent interfacial excess free energy. We also perform parametric studies to clarify how each term in the functional controls PB structure and energetics: *κ*_A,*i*_ and 
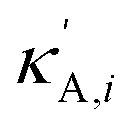
 primarily set the spatial extent of the transition on side *i*, *E*_A,*i*_ controls how strongly the profile is pulled back to bulk on side *i*, the PB plane density *ρ*^PB^ acts as the structural descriptor of the PB, and the two traction contrasts Δ*Ψ*_0_ and Δ*Ψ*_1_ control PB-localized energetic contributions in a physically interpretable way. Together, these results show how a small set of density descriptors extracted from atomistic information, combined with bulk referenced continuum coefficients, can provide a practical and transferable thermodynamic description of PBs.

## Author contributions

Jegatheesan Murugan: conceptualization; methodology; formal analysis; software; validation; visualization; writing – original draft, review & editing. Theophilus Wallis: data curation; investigation; writing – original draft, review & editing. Reza Darvishi Kamachali: conceptualization; methodology; supervision; funding acquisition; writing – review & editing.

## Conflicts of interest

The authors declare no competing financial interests.

## A Appendixes

### A1 Heaviside rewriting and local Taylor expansions

This appendix records the algebra used to rewrite the force densities so that all *q*-integrals run over a fixed domain *q* ∈ [0, ∞) and the PB geometry enters only through simple indicator functions. This is the form used in the main text to motivate the bulk–plus–traction decomposition eqn (2.12) and (2.13).

#### A.1.1 Step-function identities and tail moments on the *α* side (*r*_p_ < 0)

We use the Heaviside functionA.1.1
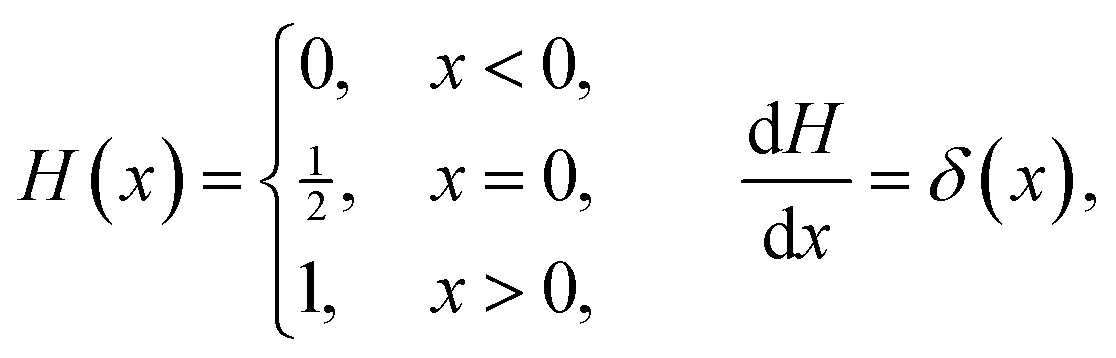
in the sense of distributions.

For a field point *r*_p_ < 0 on the *α* side, the interface-crossing condition in the *α* → *γ* channel is *r*_p_ + *q* > 0, *i.e. q* > |*r*_p_| = −*r*_p_. Hence *H*(*r*_p_ + *q*) = 0 for 0 ≤ *q* < |*r*_p_| and *H*(*r*_p_ + *q*) = 1 for *q* > |*r*_p_|, and we can rewriteA.1.2
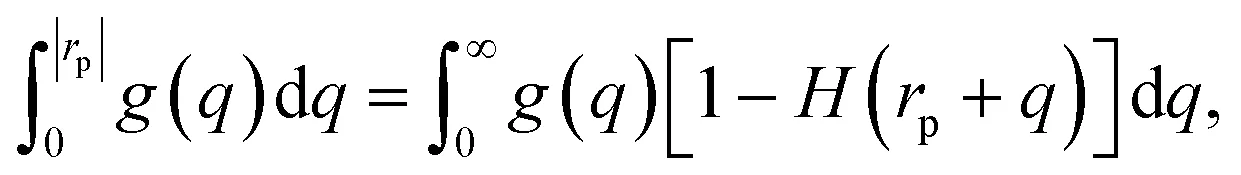
A.1.3
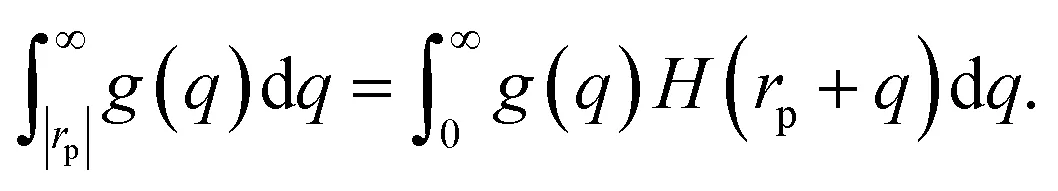


We define the *r*_p_-independent kernel momentsA.1.4
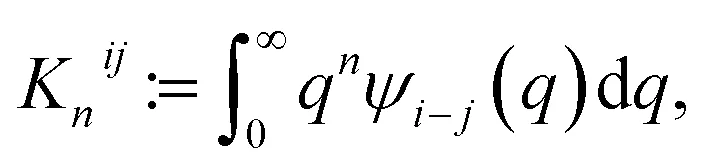
and the corresponding tail moments on the *α* side,A.1.5
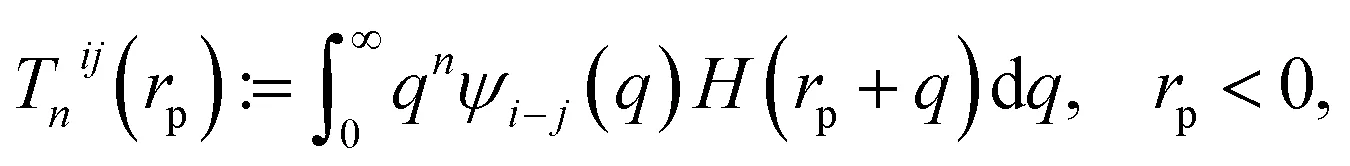
which measure the portion of the *q*^*n*^-weighted interaction range that lies beyond the point *r*_p_ toward the PB (*i.e.* the part activated once *q* > |*r*|).

We also introduce the truncated momentA.1.6
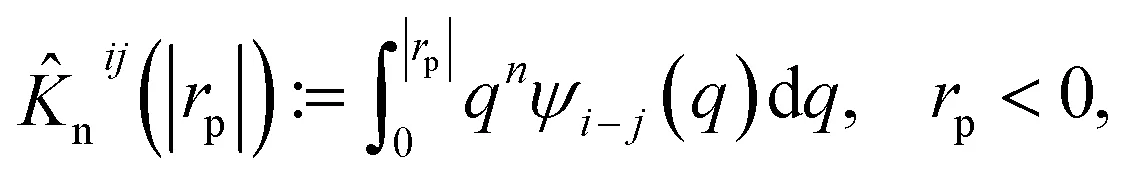
so that, by splitting the full moment at |*r*_p_|,A.1.7
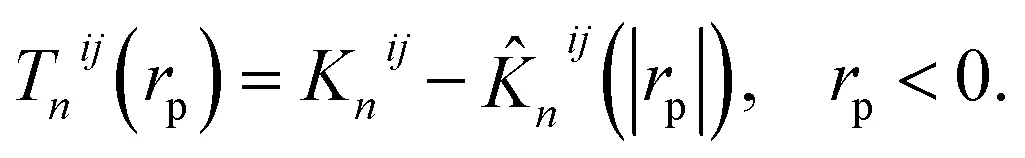


Differentiating with respect to *r*_p_ (for *r*_p_ < 0 so that |*r*_p_| = −*r*_p_) gives the useful identitiesA.1.8
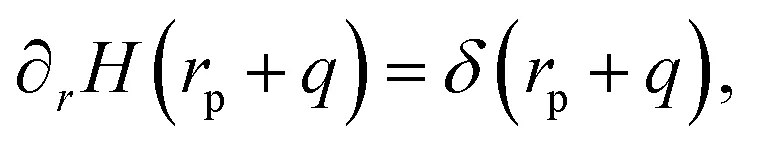
A.1.9
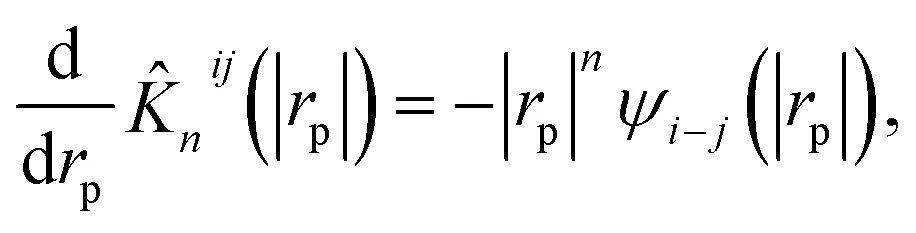
A.1.10
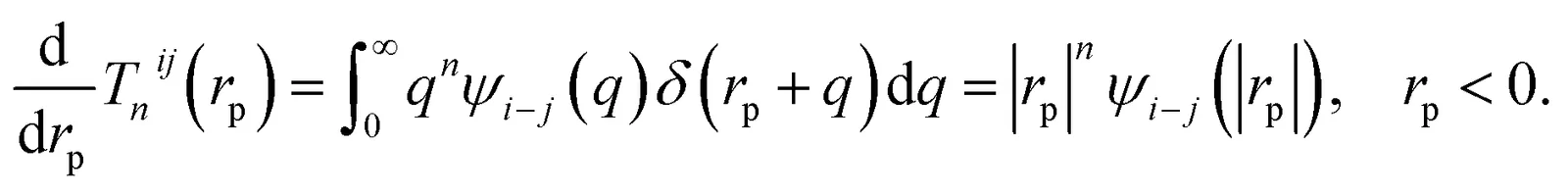


#### A.1.2 Fixed-domain form of *f*_*α*_ and local Taylor expansions

Starting from the *α*-side force density, eqn (2.7), in the main text and using the identities eqn (A.1.2) and (A.1.3), we obtain the fixed-domain formA.1.11
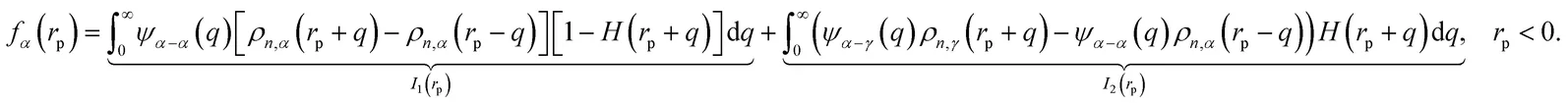
All integrals are now over *q* ∈ [0, ∞). The PB affects the force only through the switching factors *H*(*r*_p_ + *q*) and 1 − *H*(*r*_p_ + *q*).

For a smooth density field, the local Taylor expansions about the field point *r* areA.1.12
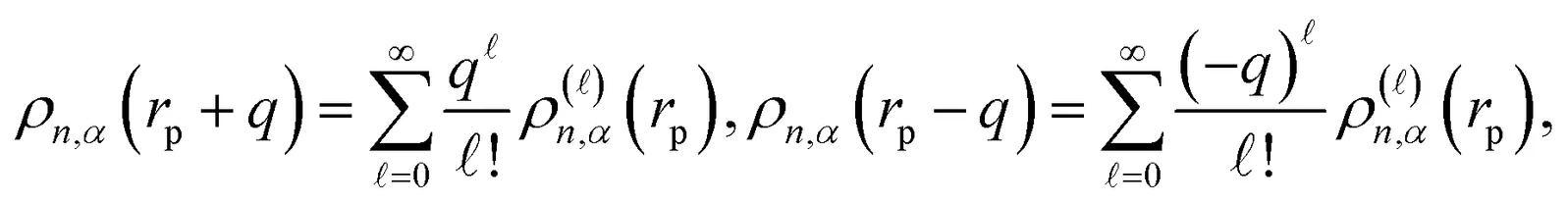
so that the symmetric difference isA.1.13
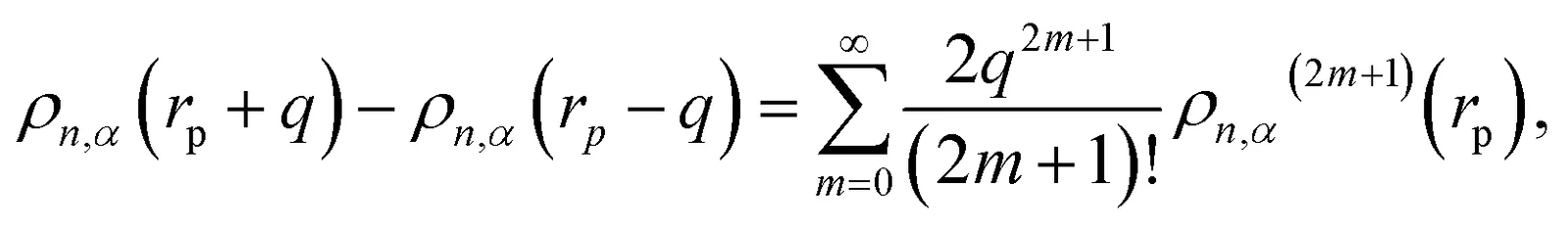
*i.e.* only odd derivatives contribute.

#### A.1.3 Bulk operator and interface channel on the *α* side

Inserting eqn (A.1.13) into *I*_1_(*r*_p_) yieldsA.1.14
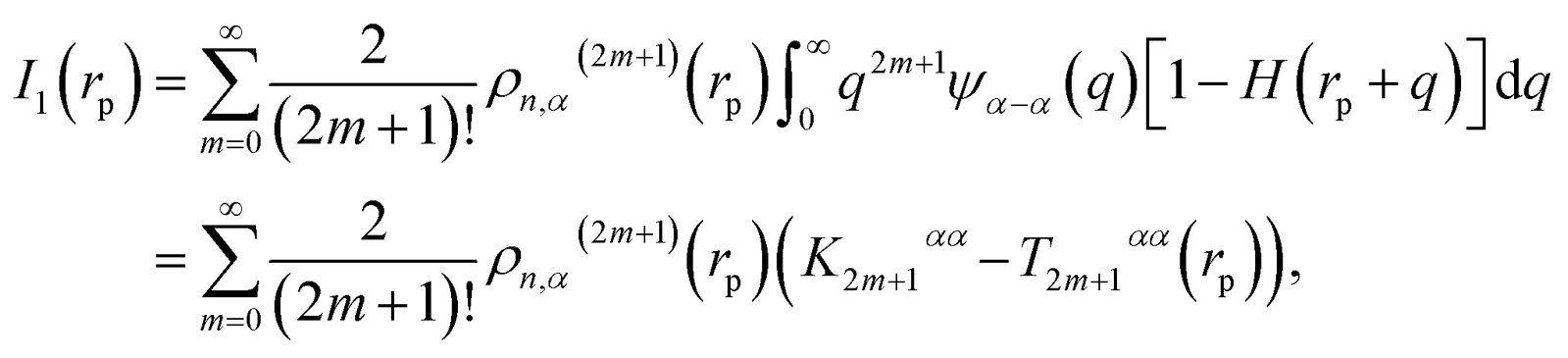
where we used the eqn (A.1.7) with *ij* = *αα*.

Next, split *I*_2_(*r*_p_) into an *αγ* part and an *αα* part:A.1.15
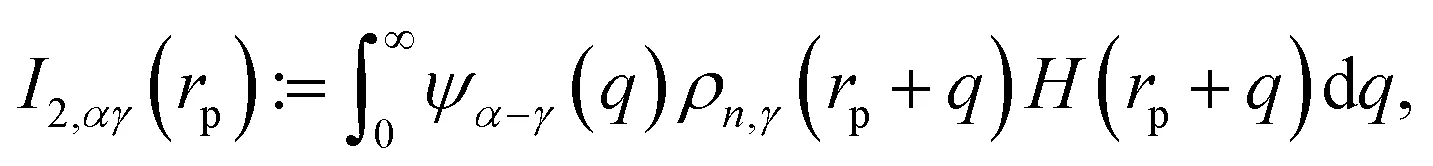
A.1.16
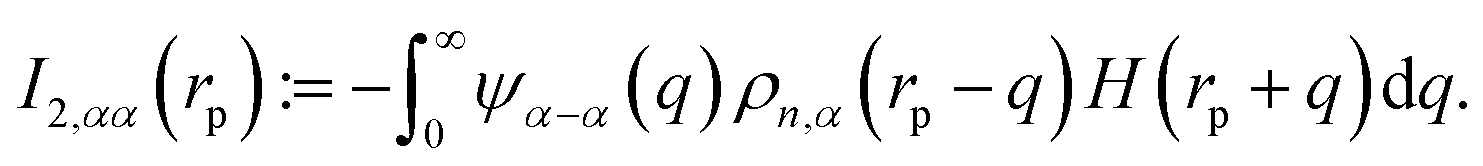
Expanding *ρ*_*n*,*α*_(*r*_p_ − *q*) in *I*_2,*αα*_ givesA.1.17
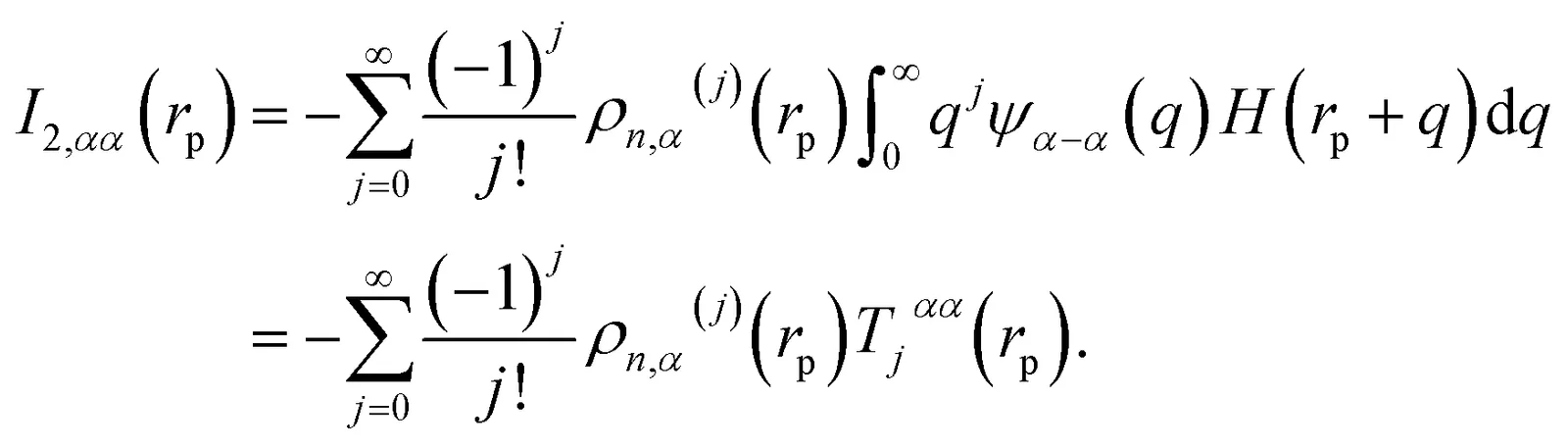


At this point the separation used in the main text is immediate:

• The bulk operator collects the constant-coefficient terms proportional to the moments *K*_2*m*+1_^*αα*^;

• All terms carrying tail moments *T*_*n*_^*ij*^(*r*_p_) belong to the interface channel, because they are switched on only when the interaction reaches across *r*_p_ = 0.

Accordingly, the constant-coefficient bulk operator on the *α* side isA.1.18
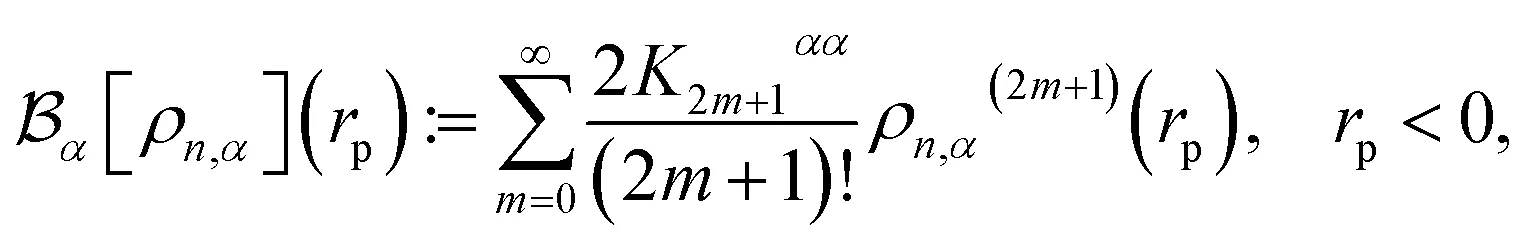
and we writeA.1.19
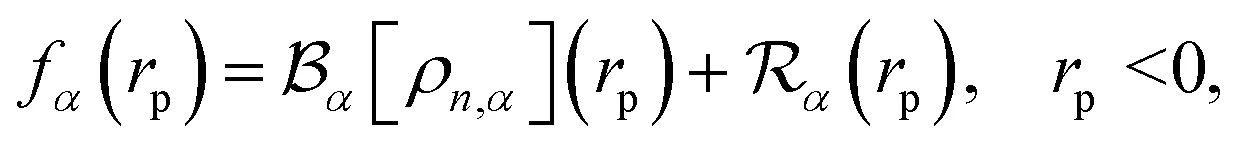
where 
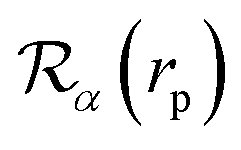
 is the remainder containing all tail-moment (Heaviside-channel) terms, including the *αγ* cross-phase contribution.

#### A.1.4 Interface channel as a boundary traction distribution

The interface remainder can be written compactly asA.1.20
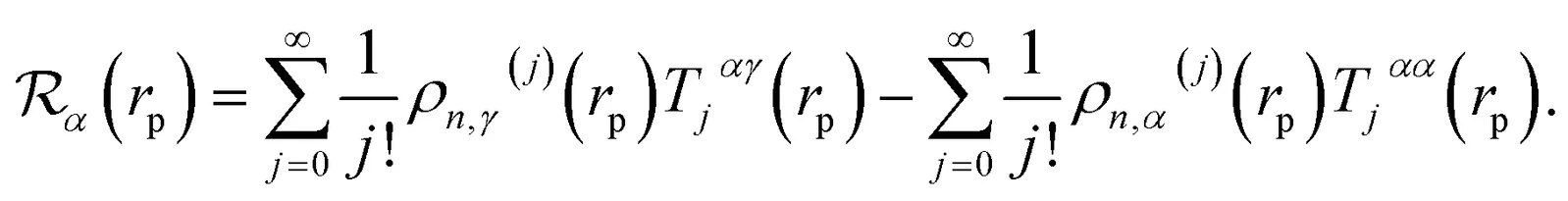


To identify the distribution supported at *r*_p_ = 0, let *φ* ∈ *C*^∞^_c_((−∞,0]) be a test function. Consider the pairing for *r*_p_ < 0:A.1.21
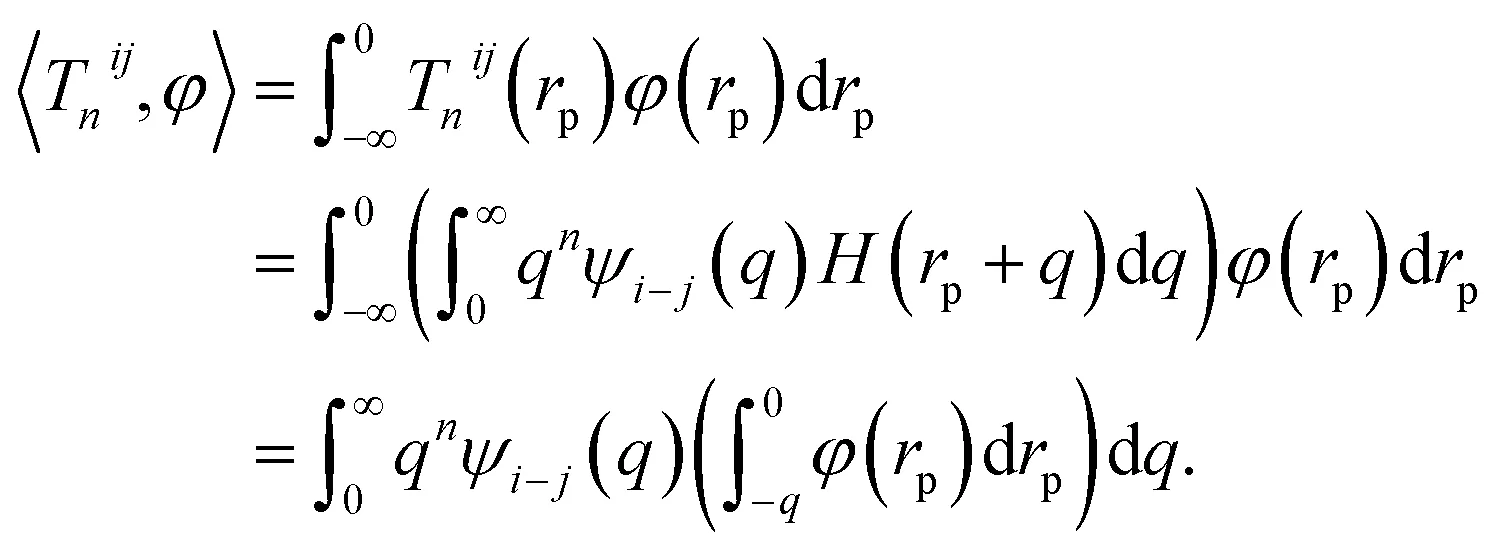
Expanding *φ* about *r*_p_ = 0 and integrating term-by-term yieldsA.1.22
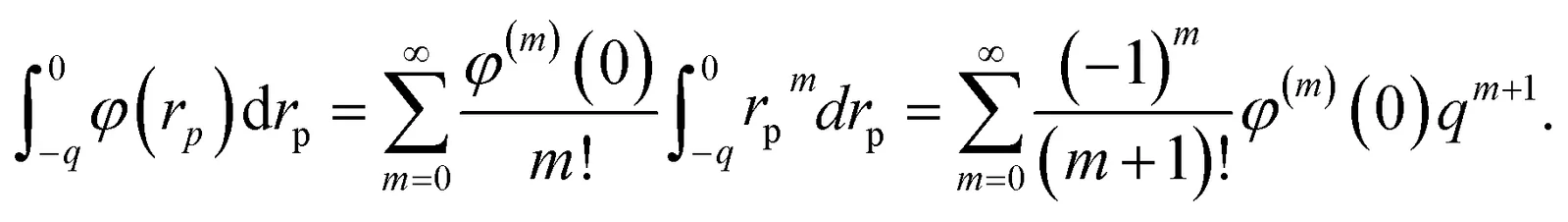
Substituting into eqn (A.1.21) givesA.1.23
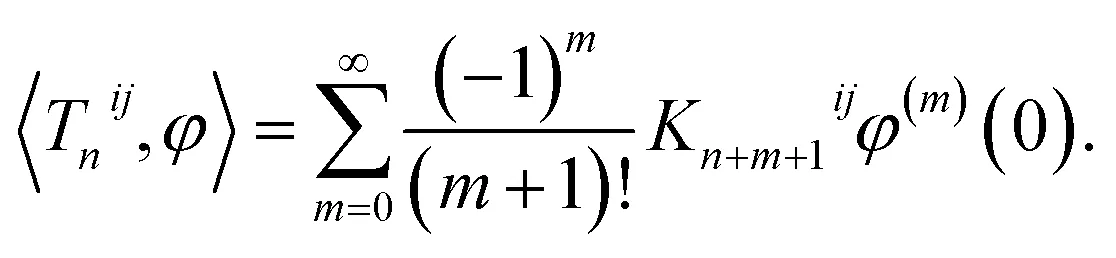
Using 〈*δ*^(*m*)^, *φ*〉 = (−1)^*m*^*φ*^(*m*)^(0), we identifyA.1.24
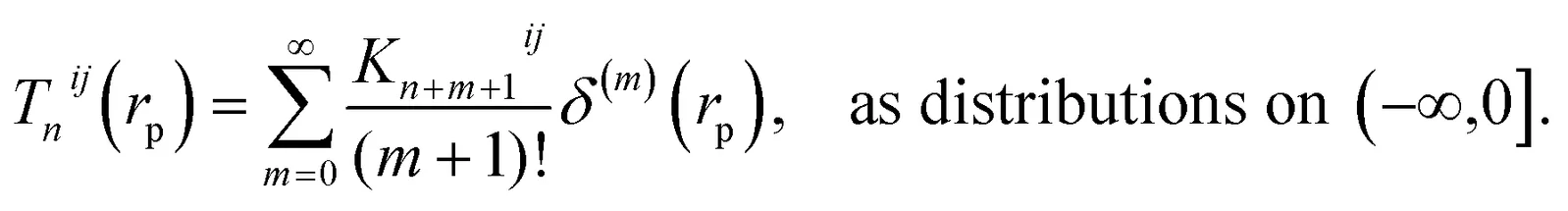


Substituting eqn (A.1.24) into eqn (A.1.20) and interpreting the density factors as one-sided smooth coefficients evaluated at the boundary (consistent with the one-sided PB slopes) yields the traction formA.1.25
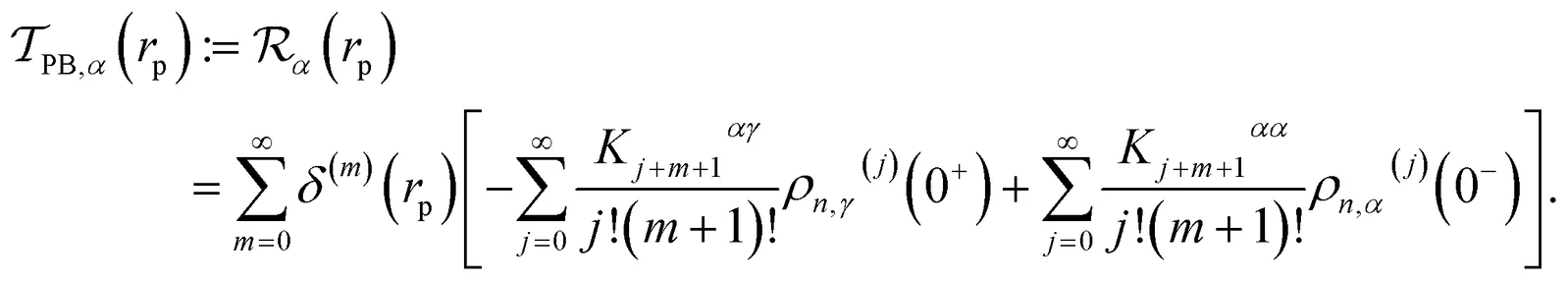


Combining eqn (A.1.18) and (A.1.25) givesA.1.26
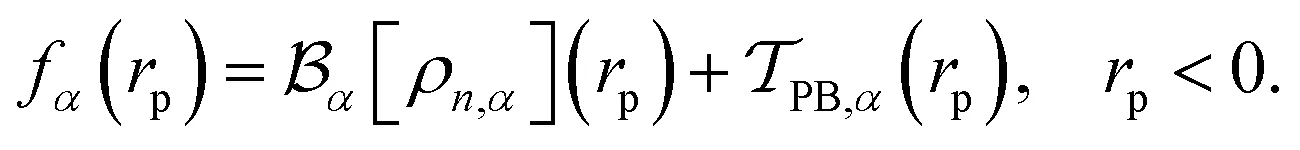


#### A.1.5 Corresponding fixed-domain form on the *γ* side (*r*_p_ > 0)

For *r*_p_ > 0 the interface-crossing condition is *r*_p_ − *q* < 0, *i.e. q* > *r*_p_. Accordingly, the appropriate indicator is *H*(*q* − *r*_p_) (not *H*(*r*_p_ + *q*), which would be identically one for *r*_p_ > 0 and *q* ≥ 0). UsingA.1.27
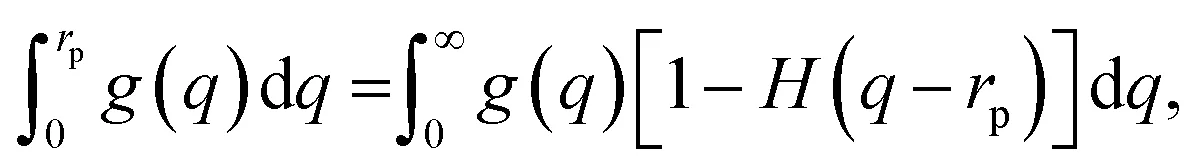
A.1.28
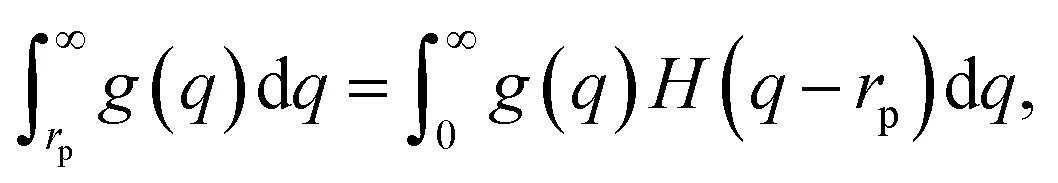
the *γ*-side force density, given in (2.11), becomesA.1.29
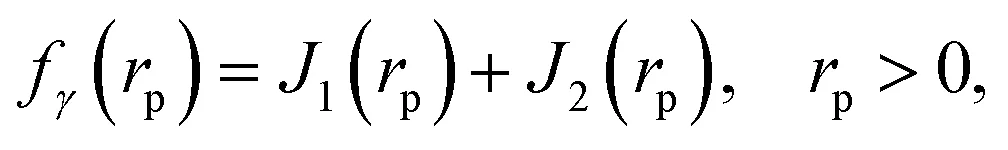
where
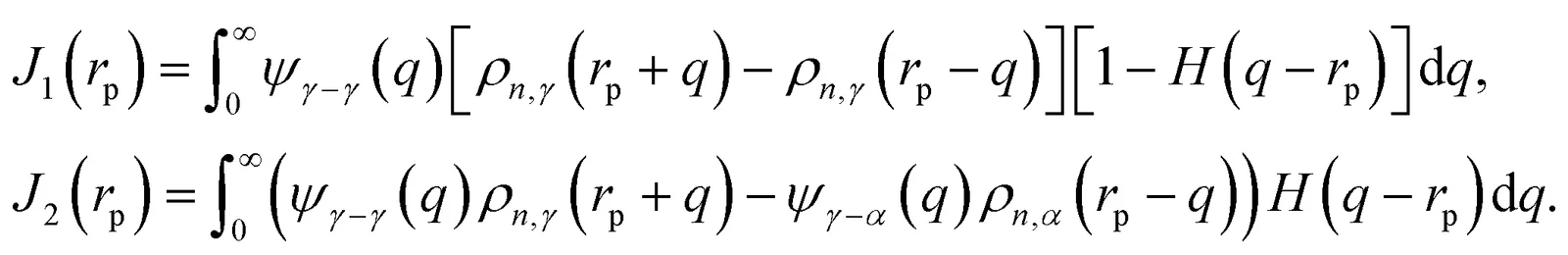


Proceeding as on the *α* side, *J*_1_(*r*_p_) produces the constant-coefficient bulk operatorA.1.30
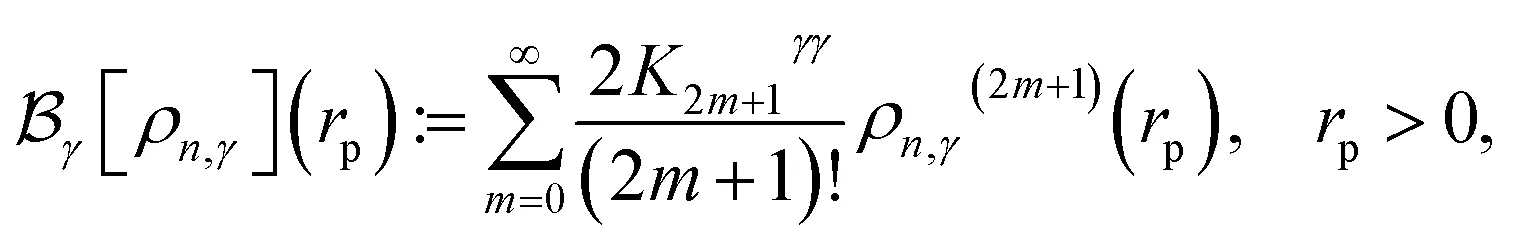
and we writeA.1.31
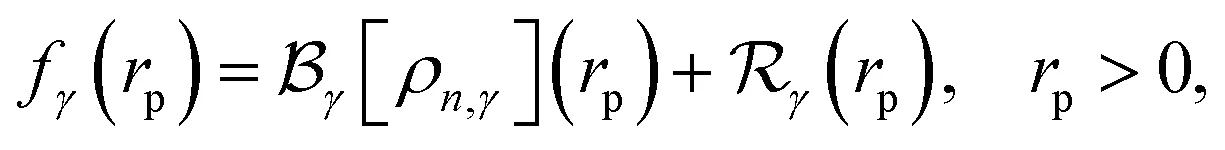
where 
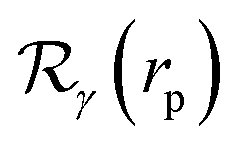
 is the remainder containing all tail-moment (Heaviside-channel) terms, including the *γα* cross-phase contribution.

For the *γ*-side, the analogue of eqn (A.1.5) isA.1.32
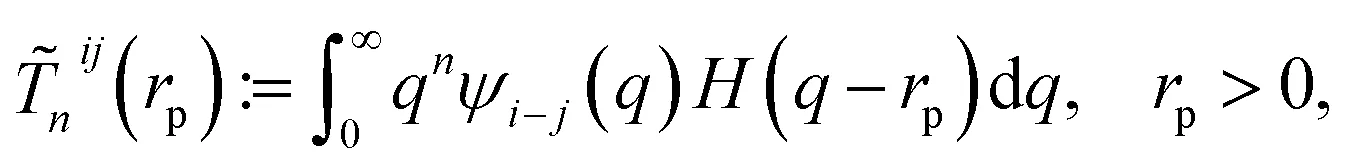
together with the elementary splittingA.1.33
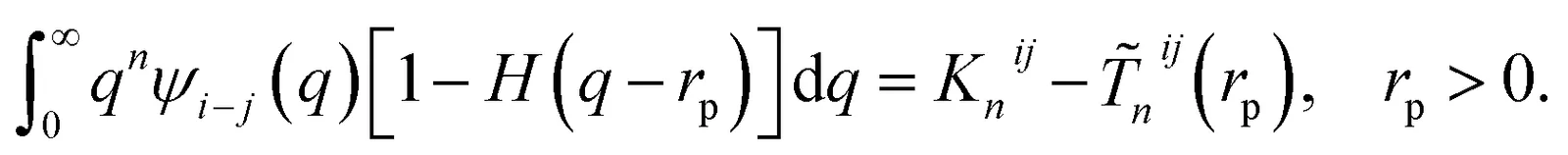


##### A.1.5.1 Bulk operator and interface channel on the *γ* side

Split *J*_2_(*r*_p_) into a *γγ* part and a *γα* part, mirroring (A.15)–(A.16):A.1.34
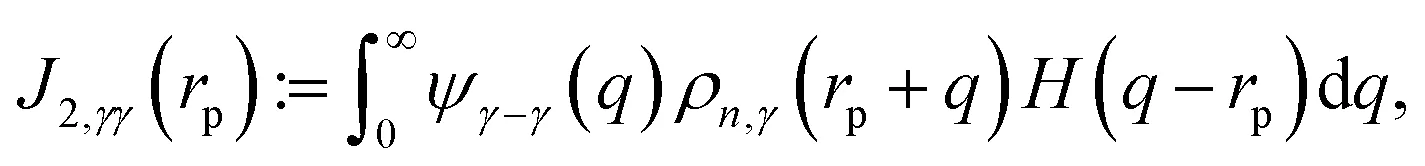
A.1.35
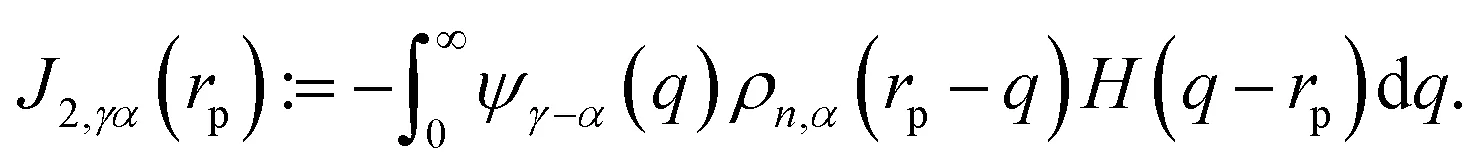
Expanding *ρ*_*n*,*γ*_(*r*_p_ + *q*) in *J*_2,*γγ*_ (ordinary Taylor series, no sign alternation since the argument is *r*_p_ + *q*) givesA.1.36
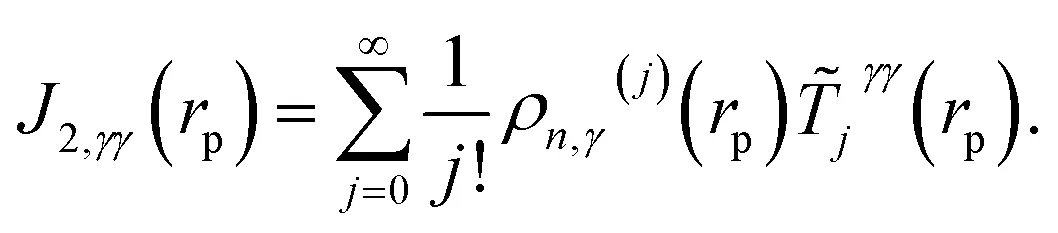
Expanding *ρ*_*n*,*α*_(*r*_p_ − *q*) in *J*_2,*γα*_ (as in eqn (A.1.17)) givesA.1.37
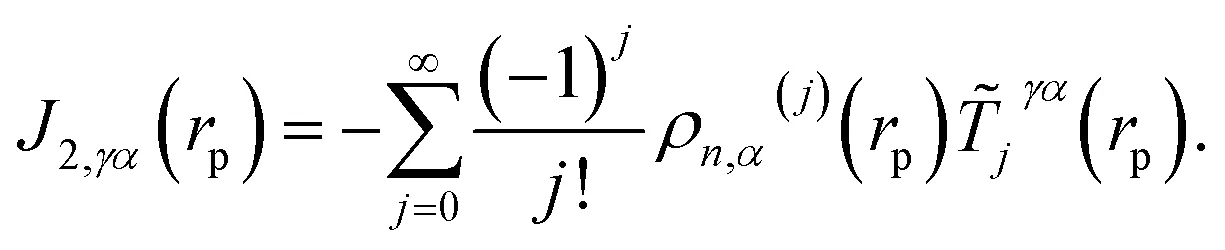
Using eqn (A.1.33), *J*_1_(*r*_p_) splits asA.1.38
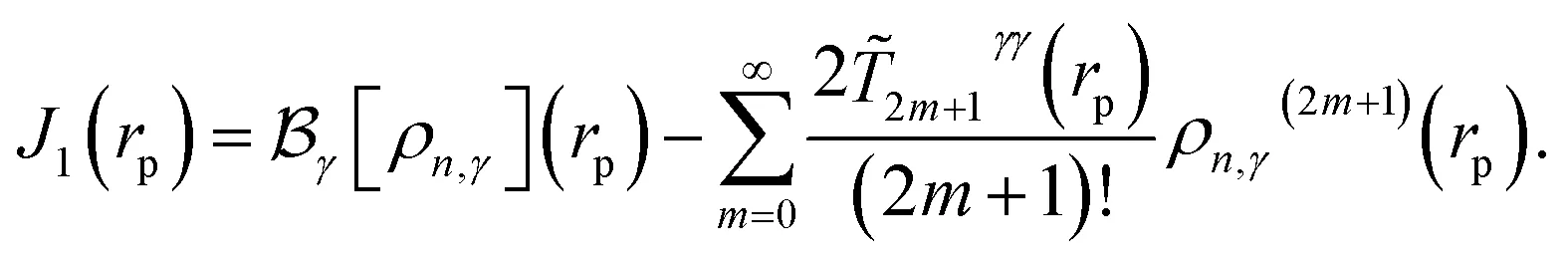
Collecting the tail part of eqn (A.1.38) with eqn (A.1.36) and (A.1.37), the *γγ* contributions combine (odd *j* from *J*_1_ plus all *j* from *J*_2,*γγ*_) into a single sum carrying alternating sign (−1)^*j*^—unlike the *α* side, where the analogous combination in eqn (A.1.20) is sign-uniform. This gives the remainderA.1.39
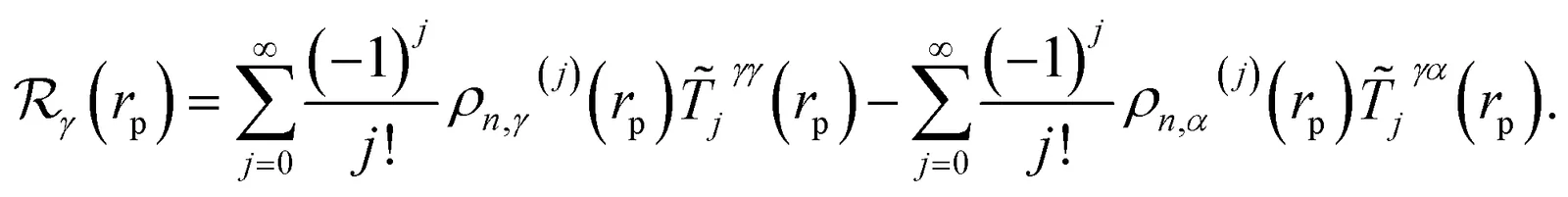


##### A.1.5.2 Interface channel as a boundary traction distribution (*γ* side)

Let *φ* ∈ *C*^∞^_c_([0,∞)) be a test function. The pairing for *r*_p_ > 0 isA.1.40
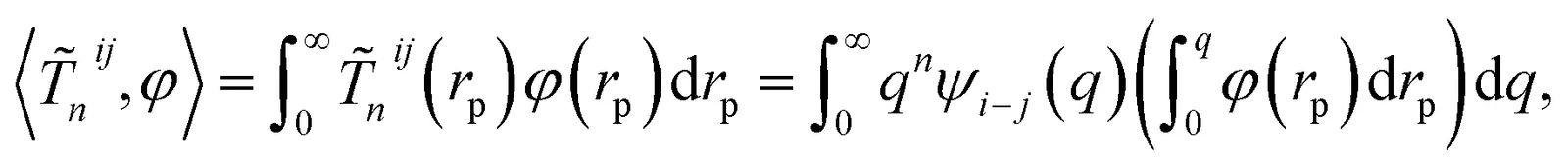
using *H*(*q* − *r*_p_) = 1 for 0 ≤ *r*_p_ < *q*. Expanding *φ* about *r*_p_ = 0,A.1.41
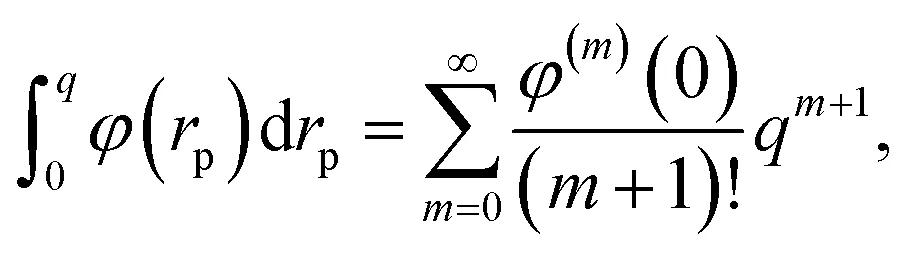
so thatA.1.42
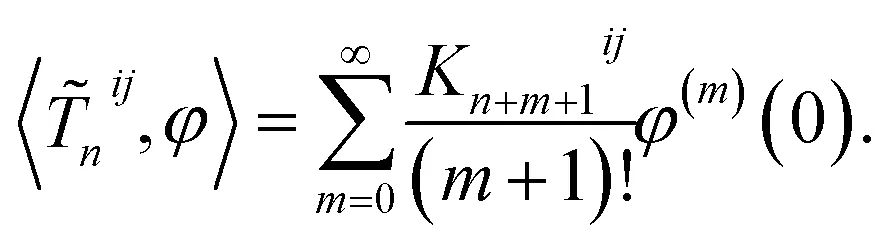
Using 〈*δ*^(*m*)^, *φ*〉 = (−1)^*m*^*φ*^(*m*)^(0), we identifyA.1.43
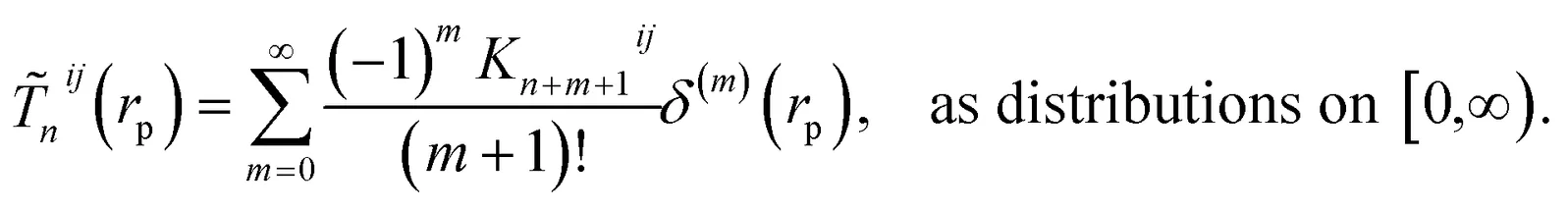
This alternating sign contrasts with the *α*-side identity eqn (A.1.24), where the equivalent expansion is sign-uniform; the difference traces to the orientation of the bounding integral ([−*q*, 0] on the *α* side *versus* [0, *q*] here).

Substituting eqn (A.1.43) into eqn (A.1.39) and evaluating the density factors at the one-sided boundary values *ρ*_*n*,*γ*_^(*j*)^ (0^+^), *ρ*_*n*,*α*_^(*j*)^ (0^−^) (consistent with eqn (A.1.25)) gives the *γ*-side traction:A.1.44
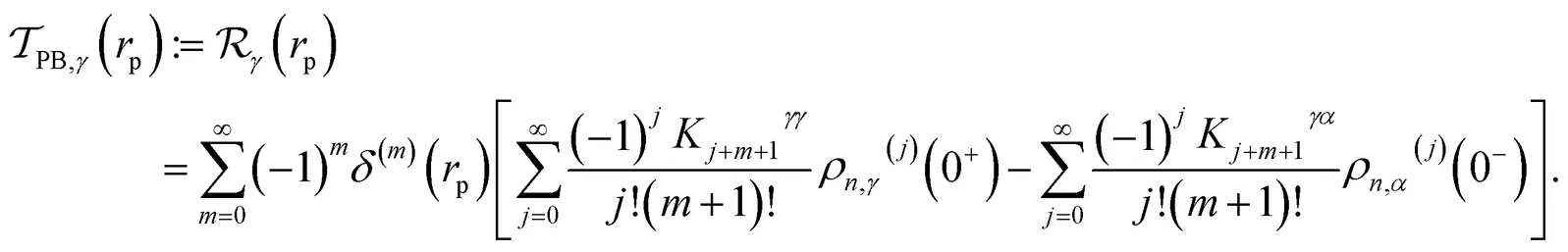


Combining eqn (A.1.30) and (A.1.44) gives the *γ*-side decomposition used in the main text:A.1.45
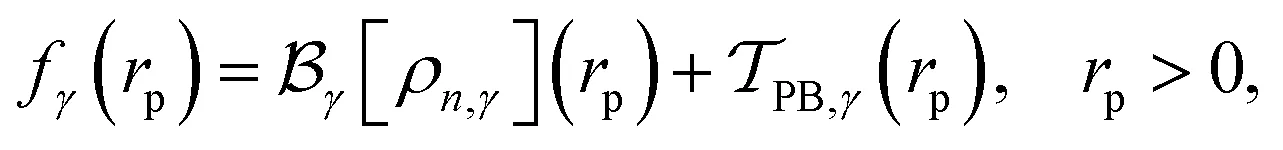
where 
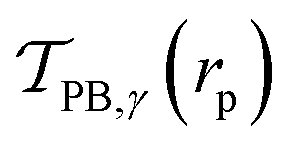
 is supported at *r*_p_ = 0 and depends only on the one-sided PB slopes *ρ*_*n*,*α*_^(*j*)^ (0^−^) and 
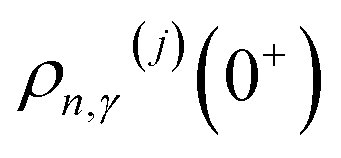
 together with kernel moments.

In the main text, this sharp (distributional) traction is replaced by the finite-width (windowed) regularization described in Section 2.2.

### A.2 Cap moments used in the finite-width PB approximation

This appendix defines the window-averaged cap moments 
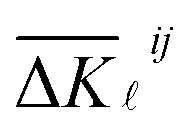
 used in Section 2.2. These quantities measure the truncated part of the *q*^*ℓ*^-weighted interaction kernel over the portion of the interaction range that reaches across the PB.

#### A.2.1 Cap integrals and window averages

For a field point *r*_p_ < 0 on the *α* side, the cross-boundary interaction is activated for *q* ≥ |*r*_p_|. In the finite-width approximation we retain only a cap of length *η*_*α*_ and defineA.2.1
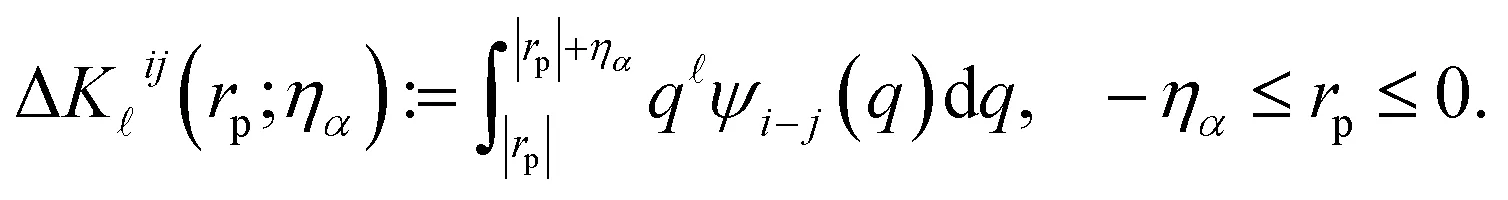
To avoid carrying explicit *r*_p_-dependence, we replace Δ*K*_*ℓ*_^*ij*^ by a window average. With *s*≔ |*r*_p_| = −*r*_p_ ∈ [0, *η*_*α*_] we setA.2.2
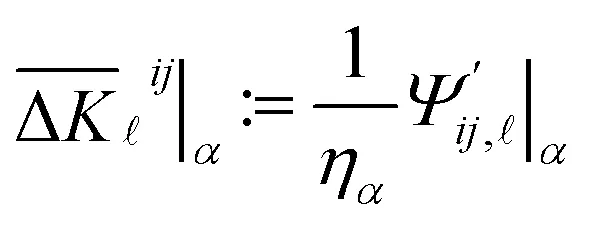
where,
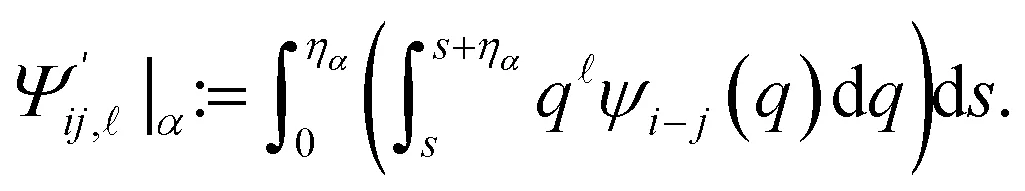


For a field point *r*_p_ > 0 on the *γ* side, the cross-boundary channel is activated when *q* ≥ *r*_p_. We defineA.2.3
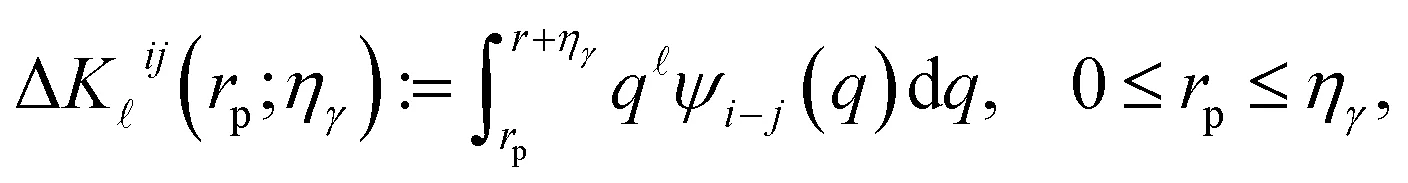
and its window averageA.2.4
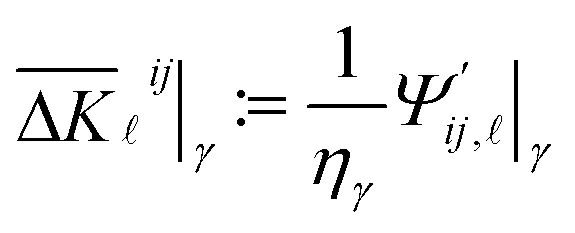
where,
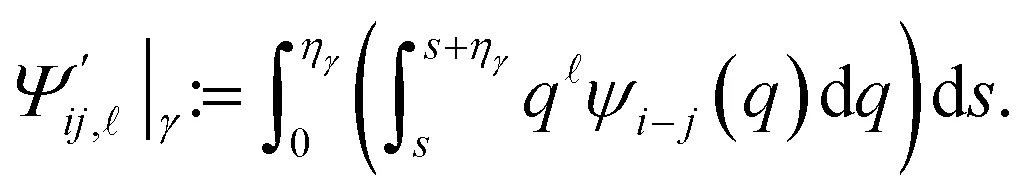


#### A.2.2 Localization replacement

In the finite-width approximation we represent the residual *r*-dependence of the cap integrals *via* localization functions *ϕ*_*i*_(*r*_p_) defined in eqn (2.16) and (2.17). The localization functions are introduced only to confine the PB correction corresponding to the finite-width PB layers and does not reconstruct the exact spatial variation of the window cap moments. So, the effective traction coefficients implicitly absorb the normalization associated with the considered localization shape.A.2.5
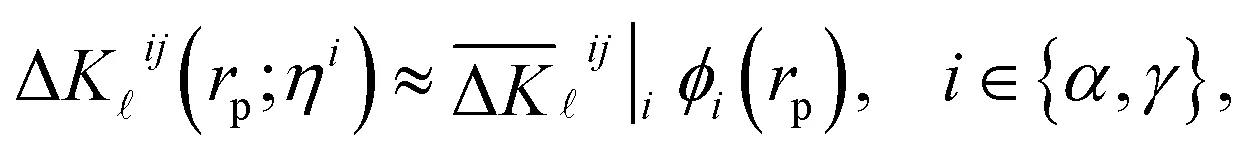
which ensures that the PB correction vanishes smoothly at the edges of the window and that the force reduces to the bulk operator in the far-field.

### A.3 Finite-difference treatment of the half-domains

At interior nodes, *j*, the derivatives are evaluated using centered differences,A.3.1
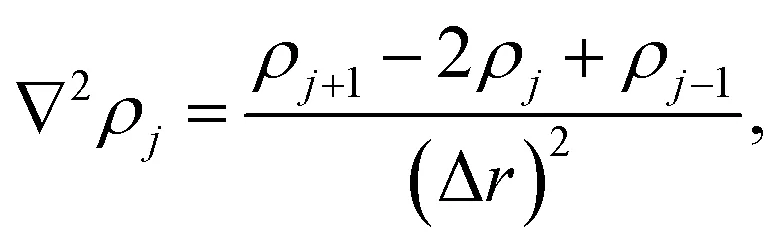
A.3.2
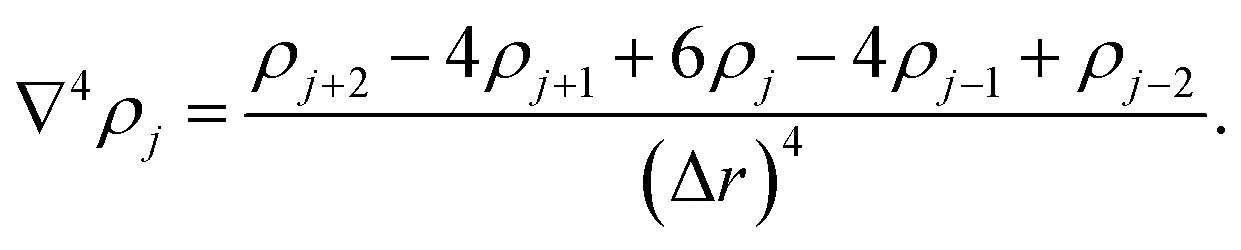
The one-sided PB slopes entering the traction term are evaluated to second order asA.3.3
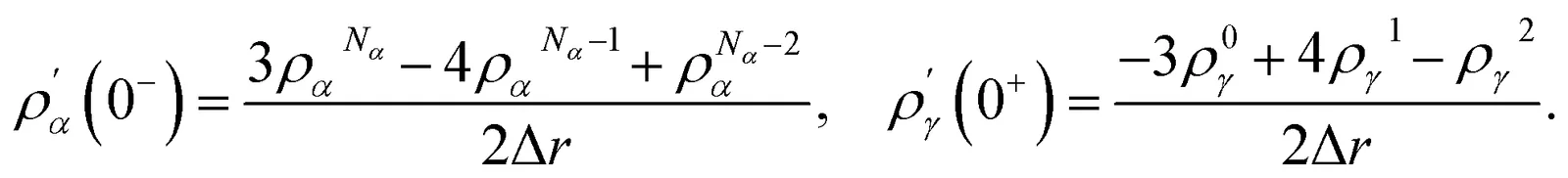
At the far-field boundaries the density is held at the bulk value *ρ*^B^_*i*_ and flat curvature is applied, *i.e.*, ∇^2^*ρ*_*i*_ = ∇^4^*ρ*_*i*_ = 0. The density at the PB node is held at *ρ*^PB^. The two nodes adjacent to each of the far-field boundaries and to the PB node are excluded from the relaxation, as they are required by the five-point finite difference stencils and by the one-sided slope expressions in eqn (A.3.3) and all remaining interior nodes are updated according to eqn (A.3.2).

At the PB node, reflected ghost-node values are used so that the curvature and higher-order derivatives on each side depend only on the density values of the corresponding phase. For the *α* side, the second derivative at the PB node isA.3.4
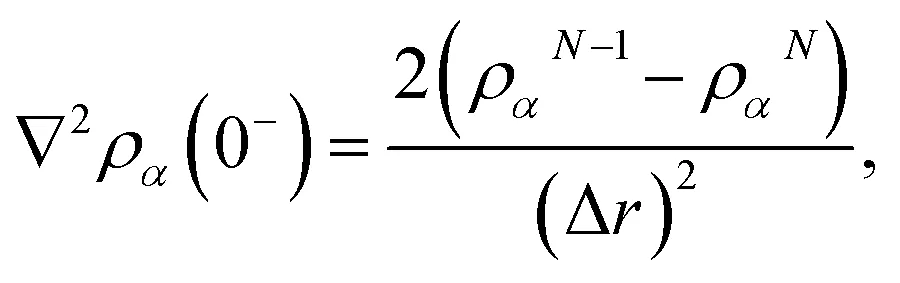
while on the *γ* side it isA.3.5
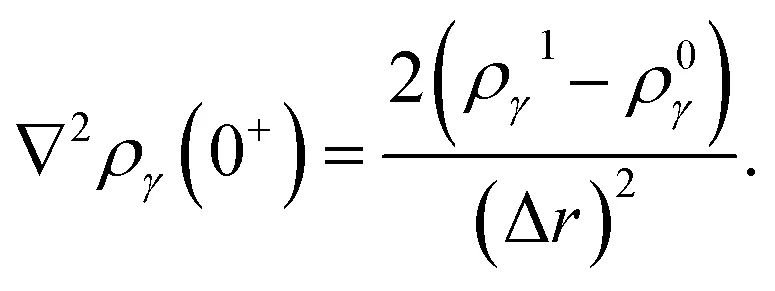
The fourth-order derivatives are evaluated using reflected-node approximations consistent with the interior five-point stencil,A.3.6
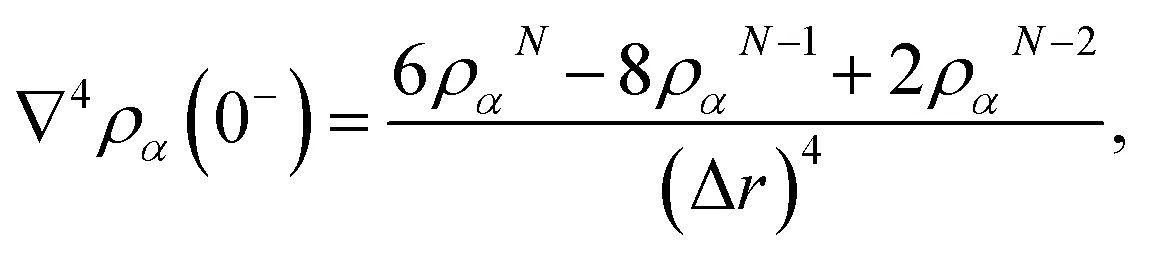
A.3.7
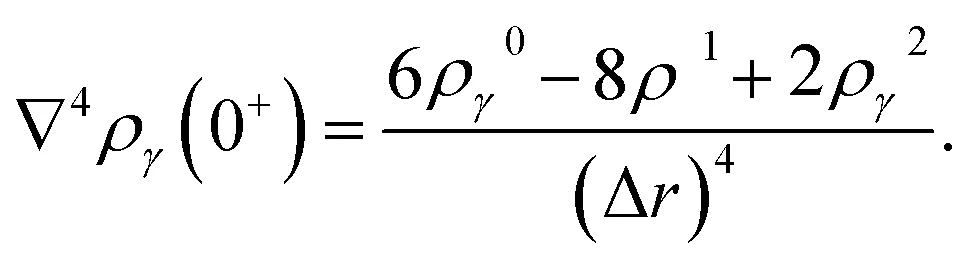
These expressions are equivalent to applying the standard finite difference stencils together with reflected ghost-node values across the PB plane.

To capture the thermodynamics about a spherical PB in 3D, the model has to be extended to 3D. While the PB free energy functional is frame invariant, its numerical extension to 3D is not straight forward due to the traction term. In the present 1D formulation, *τ*_*i*_ is constructed from the one-sided density slopes 
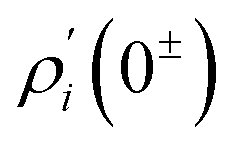
 and the half-widths *η*_*i*_ evaluated at a shared PB node, which presumes a planar PB. For a general 3D configuration, these quantities will have to be defined locally, with respect to the local normal at each node of the PB surface, and the cap moments of Appendix A.2 re-derived accordingly.

**Fig. 11:**
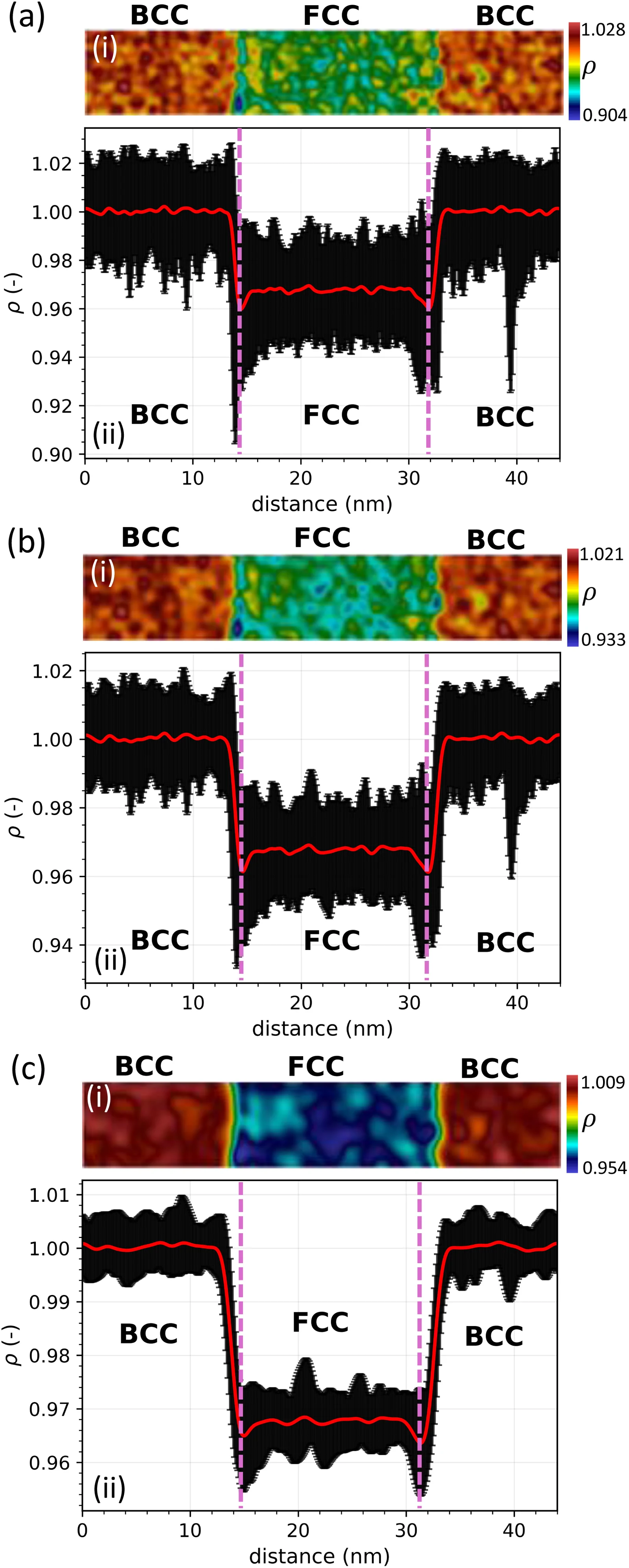
PB atomic density profiles across BCC–FCC phases for 1000 K obtained using a coarse-graining scheme. The coarse-graining of atomistic PB structure is illustrated for three different smearing radii (a) *β* = 2.4*r*^Fe^_atom_, (b) *β* = 3.0*r*^Fe^_atom_, and (c) *β* = 5.0*r*^Fe^_atom_, where *r*^Fe^_atom_ denotes the atomic radius Fe. The cross-sectional view of the underlying atomistic PB structure is shown in Fig. 3(a) of the main text. In each panel, (i) shows the atomic density field across the PB regions, while (ii) shows a continuous atomic density profile (red curve), with the range of density fluctuations in each plane, depicted by the error bars. The minima of the profiles correspond to the average atomic densities at the respective PB planes.

### A.4 Effects of coarse-graining length on PB atomic density profile

Fig. 11 shows PB atomic density profiles across BCC–FCC phases for 1000 K obtained using a coarse-graining scheme.

### A.5 Variational derivative of the density potential

Writing *C*_*i*_≔*K*^B^_A,*i*_ + *pV*^B^_A,*i*_ − *TS*^B^_A,*i*_, the local part of the excess free energy density in eqn (2.33) reduces toA.5.1
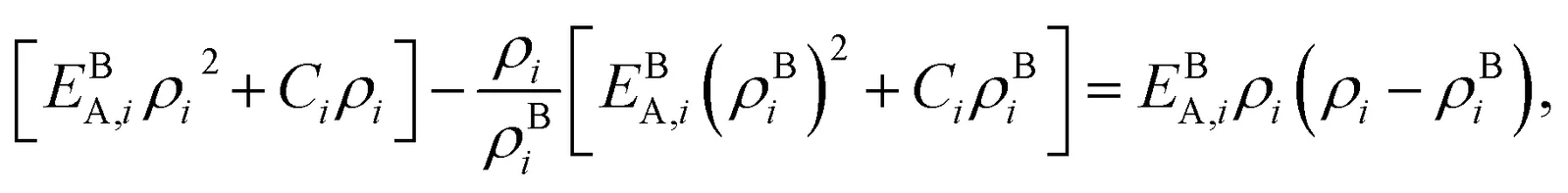
so that *C*_*i*_ cancels and does not enter the numerical implementation. Varying the excess free energy with respect to *ρ*_*i*_, while treating *τ*_*i*_ as a frozen field, givesA.5.2
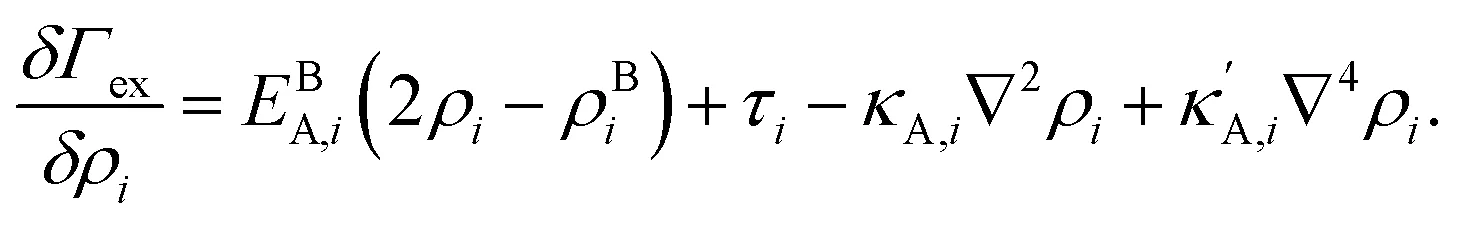
At the homogeneous bulk state eqn (A.5.2) does not vanish, but leaves *E*^B^_A,*i*_*ρ*^B^_*i*_. Without a constant offset, the equation solved in the interior nodes will not admit *ρ*_*i*_ = *ρ*^B^_*i*_ as a solution, which would be inconsistent with the bulk density prescribed in the far-field. This constant offset is the bulk potentialA.5.3
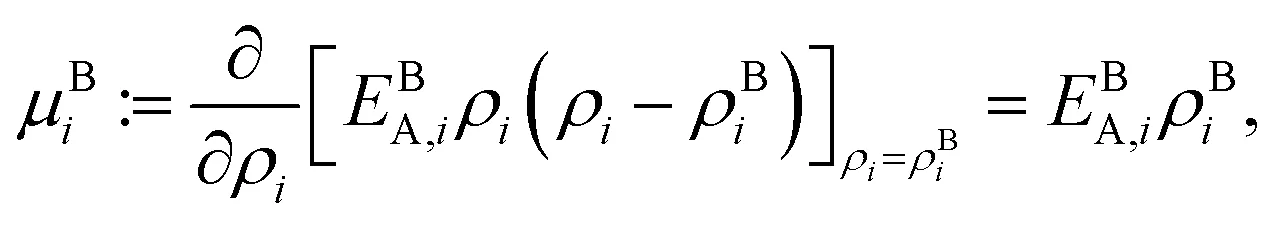
and the density potential is defined relative to it,A.5.4
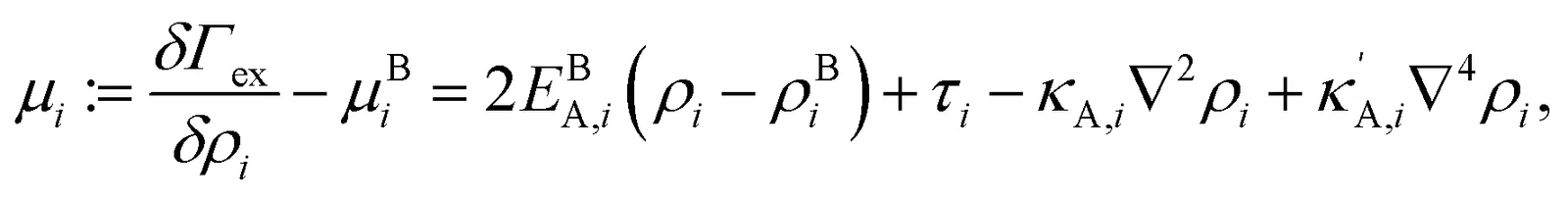
which is eqn (2.36). With this form *µ*_*i*_ vanishes at *ρ*_*i*_ = *ρ*^B^_*i*_ for any values of the coefficients, so the homogeneous bulk state is a stationary solution of the update scheme in eqn (2.37), independent of the sign of *E*^B^_A,*i*_.

The sign of *E*^B^_A,*i*_ does enter the stability of this state. Taking the local term alone, ∂*µ*_*i*_/∂*ρ*_*i*_ = 2 *E*^B^_A,*i*_ < 0, so this term by itself works against the bulk state, while the gradient terms act in the opposite sense. The second variation at *ρ*_*i*_ = *ρ*^B^_*i*_ isA.5.5
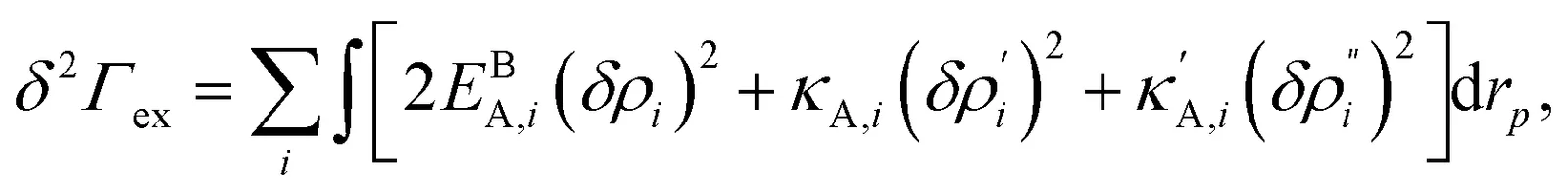
with no contribution from the constant offset. The perturbation vanishes at the PB node and at the far-field node, where the density is held fixed, and can therefore be written as *δρ*_*i*_ = ∑_*k*_*c*_*k*_ sin(*q*_*k*_*r*_p_) with *q*_*k*_ = *k*π/*L*_*i*_. These modes are orthogonal, so the second variation reduces to a sum of independent contributions with coefficientsA.5.6
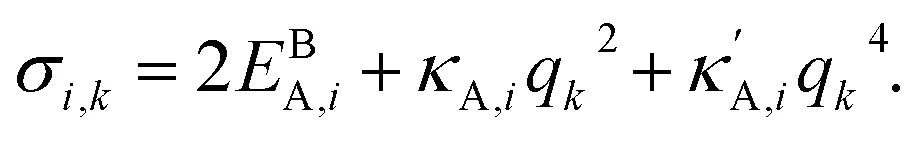
Since *κ*_A,*i*_ and 
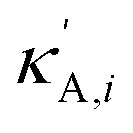
 are positive, *σ*_*i*,*k*_ increases with *k*, and it is sufficient to check the first mode. For the base case parameters of Table 1, with *L*_*α*_ = *L*_*γ*_ = 2.5 nm, eqn (A.5.6) giving positive values to *σ*_*α*,1_ J mol^−1^ and *σ*_*γ*,1_ J mol^−1^. Moreover, since the update in eqn (2.37) moves along −*µ*_*i*_, the homogeneous bulk state is a stable stationary solution for the parameter set used in the present study.

## Data Availability

The current work mainly involves theoretical developments. Further data on this study are available per reasonable request. We release the codes for this study in a follow-up investigation currently developing for binary alloy systems.
